# Supplementary material for: Clinical, laboratory and radiological characteristics and outcomes of novel coronavirus (SARS-CoV-2) infection in humans: A systematic review and series of meta-analyses
Source: PLoS One. 2020 Sep 17;15(9):e0239235. doi: 10.1371/journal.pone.0239235 (PMC7498028; doi:10.1371/journal.pone.0239235)
Supplement: S1 File — (DOCX) [file pone.0239235.s001.docx]

**Clinical, laboratory and radiological characteristics and outcomes of novel coronaviruS (SARS-CoV-2) infection in humans: A systematic review and series of meta-analyses**

**Supplementary materials**

Below we present all supplementary materials mentioned in the full text


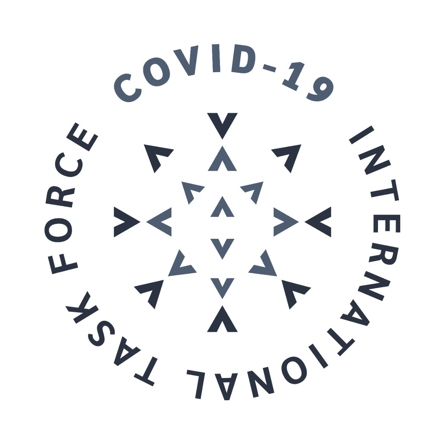


**Supplementary materials – table of contents**

# **Supplementary appendices**

S1 Appendix. Search strategy …………...………………….…….………………………..5

S2 Appendix. Member list – InterNetCOVID-19 …………….…...……………………….7

S3 Checklist. PRISMA 2009 checklist……………………………………………………..9

# **Supplementary tables**…………….………………………………....………………………….12

S1 Table. Characteristics of included studies.

S2 Table. Characteristics of studies excluded because of overlapping patients.

S3 Table. Other excluded studies and reasons for exclusion.

S4 Table. Risk of bias assessment of observational studies.

S5 Table. Risk of bias assessment of randomized controlled trials.

S6 Table. Risk of bias assessment of epidemiological reports.

# **Supplementary figures**.………………………….…………………………….………………37

S1 Figure. Risk of bias assessment for observational studies.

S2 Figure. Risk of bias assessment for epidemiological studies.

S3 Figure. Meta-analysis of the incidence of fever (overall COVID 19 patient group)

S4 Figure. Meta-analysis of the incidence of fever (severely ill patient group)

S5 Figure. Meta-analysis of the incidence of cough (overall COVID 19 patient group)

S6 Figure. Meta-analysis of the incidence of cough (severely ill patient group)

S7 Figure. Meta-analysis of the incidence of asymptomatic patients (overall COVID 19 patient group)

S8 Figure. Meta-analysis of the incidence of mortality (overall COVID 19 patient group)

S9 Figure. Meta-analysis of the incidence of mortality (severely ill patient group)

S10 Figure. Meta-analysis of the incidence of pneumonia (overall COVID 19 patient group)

S11 Figure. Meta-analysis of the incidence of Dyspnea (overall COVID 19 patient group)

S12 Figure. Meta-analysis of the incidence of Dyspnea (moderately/severely ill patient group)

S13 Figure. Meta-analysis of the incidence of Dyspnea (severely ill patient group)

S14 Figure. Meta-analysis of the incidence of Sore throat (overall COVID 19 patient group)

S15 Figure. Meta-analysis of the incidence of Sore throat (severely ill patient group)

S16 Figure. Meta-analysis of the incidence of Gastrointestinal symptoms (overall COVID 19 patient group)

S17 Figure. Meta-analysis of the incidence of gastro-intestinal symptoms (overall COVID 19 patient group)

S18 Figure. Meta-analysis of the incidence of gastro-intestinal symptoms (severely ill patient group)

S19 Figure. Meta-analysis of the incidence of Fatigue (overall COVID 19 patient group

S20 Figure. Meta-analysis of the incidence of Headache (overall COVID 19 patient group)

S21 Figure. Meta-analysis of the incidence of positive CT findingS (overall COVID 19 patient group)

S22 Figure. Meta-analysis of the incidence of GGO Unilateral patients

S23 Figure. Meta-analysis of the incidence of any GGO (overall COVID 19 patient group)

S24 Figure. Meta-analysis of the incidence of any GGO (severely ill patient group)

S25 Figure. Meta-analysis of the incidence of consolidation (overall COVID 19 patient group)

S26 Figure. Meta-analysis of the incidence of consolidation (severely ill patient group)

S27 Figure. Meta-analysis of the incidence of septal thickening (overall COVID 19 patient group)

S28 Figure. Meta-analysis of the incidence of septal thickening (severely ill patient group)

S29 Figure. Meta-analysis of the incidence of low WBC (overall COVID 19 patient group)

S30 Figure. Meta-analysis of the incidence of elevated WBC (overall COVID 19 patient group)

S31 Figure. Meta-analysis of the incidence of elevated WBC (severely ill patient group)

S32 Figure. Meta-analysis of the incidence of low Lymphocytes (overall COVID 19 patient group)

S33 Figure. Meta-analysis of the incidence of low Lymphocytes (severely ill patient group)

S34 Figure. Meta-analysis of the incidence of overall Lymphocytes levels (severely ill patient group)

S35 Figure. Meta-analysis of the incidence of elevated Lymphocytes (overall COVID 19 patient group)

S36 Figure. Meta-analysis of the incidence of low Neutrophils (overall COVID 19 patient group)

S37 Figure. Meta-analysis of the incidence of low platelets (overall COVID 19 patient group)

S38 Figure. Meta-analysis of the incidence of elevated platelets (overall COVID 19 patient group)

S39 Figure. Meta-analysis of the incidence of elevated CRP (overall COVID 19 patient group)

S40 Figure. Meta-analysis of the incidence of elevated CRP (overall COVID 19 patient group)

S41 Figure. Meta-analysis of the incidence of elevated PCT (overall COVID 19 patient group)

S42 Figure. Meta-analysis of the incidence of elevated PCT (severely ill patient group)

S43 Figure. Meta-analysis of the incidence of elevated IL6 (overall COVID 19 patient group)

S44 Figure. Meta-analysis of the incidence of elevated IL6 (severely ill patient group)

S45 Figure. Meta-analysis of the incidence of elevated ESR (overall COVID 19 patient group)

S46 Figure. Meta-analysis of the incidence of CRP levels (overall COVID 19 patient group)

S47 Figure. Meta-analysis of the incidence of elevated ALT (overall COVID 19 patient group)

S48 Figure. Meta-analysis of the incidence of elevated ALT (severely ill patient group)

S49 Figure. Meta-analysis of the incidence of elevated AST (overall COVID 19 patient group)

S50 Figure. Meta-analysis of the incidence of elevated AST (severely ill patient group)

S51 Figure. Meta-analysis of the incidence of elevated AST levels in those with high AST (overall COVID 19 patient group)

S52 Figure. Meta-analysis of the incidence of high dimer (overall COVID 19 patient group)

S53 Figure. Meta-analysis of the incidence of high dimer (severely ill patient group)

S54 Figure. Meta-analysis of the incidence of Troponin (overall COVID 19 patient group)

S55 Figure. Meta-analysis of the incidence of Dialysis (overall COVID 19 patient group)

S56 Figure. Meta-analysis of the incidence of severely ill (overall COVID 19 patient group)

S57 Figure. Meta-analysis of the incidence of ECMO (overall COVID 19 patient group)

S58 Figure. Meta-analysis of the incidence of ECMO (severely ill patient group)

S59 Figure. Meta-analysis of the incidence of invasive ventilation (overall COVID 19 patient group)

S60 Figure. Meta-analysis of the incidence of invasive ventilation (severely ill patient group)

S61 Figure. Meta-analysis of the incidence of ICU admission (overall COVID 19 patient group)

S62 Figure. Meta-analysis of the incidence of ICU admission (severely ill patient group)

S63 Figure. Meta-analysis of the incidence of Steroids (overall COVID 19 patient group)

S64 Figure. Meta-analysis of the incidence of Steroids (severely ill patient group)

S65 Figure. Meta-analysis of the incidence of Immunoglobulin (overall COVID 19 patient group)

S66 Figure. Meta-analysis of the incidence of Immunoglobulin (severely ill patient group)

S67 Figure. Meta-analysis of the incidence of Antivirals (overall COVID 19 patient group)

S68 Figure. Meta-analysis of the incidence of Antiviral (severely ill patient group)

S69 Figure. Meta-analysis of the incidence of Antibiotic (overall COVID 19 patient group)

S70 Figure. Meta-analysis of the incidence of Antibiotic (severely ill patient group)

S71 Figure. Meta-analysis of the incidence of non-invasive ventilation (overall COVID 19 patient group)

S72 Figure. Meta-analysis of the incidence of non-invasive ventilation (severely ill patient group)

S73 Figure. Meta-analysis of the incidence of Oxygen therapy (overall COVID 19 patient group)

S74 Figure. Meta-analysis of the incidence of age (overall COVID 19 patient group)

S75 Figure. Meta-analysis of the incidence of age (severely ill patient group)

S76 Figure. Meta-analysis of the incidence of Sex (overall COVID 19 patient group)

S77 Figure. Meta-analysis of the incidence of Sex (severely ill patient group)

S78 Figure. Meta-analysis of the incidence of incubation time (overall COVID 19 patient group)

S79 Figure. Meta-analysis of the incidence of incubation time (severely ill patient group)

S80 Figure. Meta-analysis of the incidence of length of hospital stay (overall COVID 19 patient group

# Supplementary appendices

**S1 Appendix. Search Strategy**

1. **PubMed - Inception up to Mar/22/2020**

(((((coronavirus[MeSH Terms]) OR coronaviruSinfections[MeSH Terms]) OR "betacoronavirus"[MeSH Terms]) OR "betacoronaviruS1"[MeSH Terms]) OR (CoronaviruseSOR “CoronaviruSInfection” OR "COVID-19" OR “CoronaviruSInfection Disease 2019” OR “2019 Novel CoronaviruSInfection” OR “2019-nCoV Infection” OR “2019 nCoV Infection” OR “2019-nCoV Infections” OR Betacoronavirus* OR “Novel CoronaviruSPneumonia” OR “2019 novel coronavirus" OR “coronaviruSdisease 2019” OR “nCoV” OR covid* OR “bat coronavirus”))

Limit: **From 20200219-20200322**

1. **Embase (Elsevier) - Inception up to Mar/22/2020**

'coronavirinae'/exp OR 'betacoronavirus'/exp OR 'betacoronaviruS1'/exp OR coronaviruseSOR 'coronaviruSinfection'/exp OR 'coronaviruSinfection' OR 'covid-19' OR 'coronaviruSinfection disease 2019' OR '2019 novel coronaviruSinfection' OR '2019-ncov infection' OR '2019 ncov infection' OR '2019-ncov infections' OR betacoronavirus* OR 'novel coronaviruSpneumonia' OR '2019 novel coronavirus'/exp OR '2019 novel coronavirus' OR 'coronaviruSdisease 2019' OR 'ncov' OR covid* OR “bat coronavirus”

Limit: Publication yearSf**rom 20200219-20200322**

Limit: [Embase not in MEDLINE]

1. **Latin American and Caribbean Center on Health ScienceSInformation (LILACS) - Inception up to Mar/22/2020**

MH:("CoronaviruSInfection") OR ("InfeccioneSpor Coronavirus") OR ("InfecçõeSpor Coronavirus") OR ("CoronaviruSInfection") OR ("Infection, Coronavirus") OR ("Infections, Coronavirus") OR "Coronavirus" OR "Coronavirus" OR "Coronavirus" OR “bat coronavirus” OR MH:C02.782.600.550.200$ OR MH:B04.820.504.540.150$

Publication date **20200219-20200322**

**d.ScopuS (Elsevier) - Inception up to Mar/22/2020**

( "coronavirus" OR "coronaviruSinfections" OR "betacoronavirus" OR "betacoronaviruS1" OR "coronaviruses" OR "CoronaviruSInfection" OR "COVID-19" OR "CoronaviruSInfection Disease 2019" OR "2019 Novel CoronaviruSInfection" OR "2019-nCoV Infection" OR "2019 nCoV Infection" OR "2019-nCoV Infections" OR "betacoronavirus*" OR "Novel CoronaviruSPneumonia" OR "2019 novel coronavirus" OR "coronaviruSdisease 2019" OR "nCoV" OR "covid" OR "coronavirinae" OR “bat coronavirus”) AND NOT INDEX ( medline ) AND Publication date limit **March 2020**

**e. Cochrane CENTRAL - Inception up to Mar/22/2020**

(MeSH descriptor: [Coronavirus] explode all treeSOR MeSH descriptor: [Betacoronavirus] explode all trees

OR MeSH descriptor: [CoronaviruSInfections] explode all treeSOR Coronavirus* OR betacoronavirus* OR nCoV* OR novel coronavirus* OR novel corona viruSOR covid* OR “bat coronavirus”)


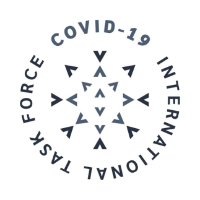


| **S2 Appendix. List of members of the International Network of CoronaviruSDisease 2019 (InterNetCOVID-19)** | |
| --- | --- |
| **Core members** |  |
| **Brazil** |  |
| Israel Júnior BorgeSdo Nascimento | University Hospital and School of Medicine, Universidade Federal de MinaSGerais, Belo Horizonte, MinaSGerais, 30130-100 Brazil. |
| Milena Soriano Marcolino | University Hospital and School of Medicine, Universidade Federal de MinaSGerais, Belo Horizonte, MinaSGerais, 30130-100 Brazil. |
| Silvana Mangeon MeirelleSGuimarães | University Hospital and School of Medicine, Universidade Federal de MinaSGerais, Belo Horizonte, MinaSGerais, 30130-100 Brazil. |
| ViniciuSTassoni Civile | Cochrane Brazil; Evidence-Based Health Program, Universidade Federal de São Paulo, São Paulo, 04021-001 Brazil. |
| Nelson CanvaSJunior | Cochrane Brazil; Universidade Paulista, São Paulo, 04057-000 Brazil. |
| **Germany** |  |
| Thilo von Groote | Department of Anesthesiology, Intensive Care and Pain Medicine, University of Münster, Münster, 48149 Germany. |
| Hebatullah Mohamed Abdulazeem | Department of Sport and Health Sciences, Technische Universität München, Munich, 80333 Germany. |
| **Australia** |  |
| Ishanka Weerasekara | School of Health Sciences, Faculty of Health and Medicine, The University of Newcastle, Callaghan, 2308 Australia |
| **Croatia** |  |
| Nensi Cacic | Cochrane Croatia, University of Split School of Medicine, Split, 21000 Croatia. |
| Ana Jeroncic | Cochrane Croatia, University of Split School of Medicine, Split, 21000 Croatia. |
| Tina Poklepovic Pericic | Cochrane Croatia, University of Split School of Medicine, Split, 21000 Croatia. |
| Ana Marusic | Cochrane Croatia, University of Split School of Medicine, Split, 21000 Croatia. |
| Irena Zakarija-Grkovic | Cochrane Croatia, University of Split School of Medicine, Split, 21000 Croatia. |
| Livia Puljak | Cochrane Croatia, University of Split School of Medicine, Split, 21000 Croatia. |
| **Canada** |  |
| Nicola Roberto Bragazzi | Laboratory for Industrial and Applied MathematicS (LIAM), Department of MathematicS and Statistics, York University, Toronto, Ontario, M3J 1P3 Canada |
| **Sri Lanka** |  |
| Umesh Jayarajah | Department of Surgery, Faculty of Medicine, University of Colombo, Colombo, 00700 Sri Lanka. |
| **United StateS of America** |  |
| Dónal P. O’Mathúna | Helene Fuld Health Trust National Institute for Evidence-based Practice in Nursing and Healthcare, College of Nursing, The Ohio State University, Columbus, Ohio, 43210 United StateS of America |
| **Sweden** |  |
| Maria Bjorklund | Faculty of Medicine, Lund University, Lund, SE-221-00, Sweden |
| **United Kingdom** |  |
| Catherine Henderson | Swanscoe Communications, Bollington, Macclesfield, SK10 5JH United Kingdom. |
| **Affiliate Members** |  |
| Meisam Abdar Esfahani | Cochrane Iran Associate Centre, National Institute for Medical Research Development, Tehran, 16846 Iran |
| Ahmad S ofi-Mahmudi | Cochrane Iran Associate Centre, National Institute for Medical Research Development, Tehran, 16846 Iran |
| Mohammad Altujjar | Department of Internal Medicine, University of Toledo, Toledo, Ohio, 43606 United StateS of America |
| Maoyi Tian | The George Institute for Global Health, University of New South Wales, Sydney, New South Wales, 2052 Australia |
| Diana Maria CespedeSArcani | Department of Cardiovascular and Thoracic Surgery, Zhongnan Hospital, Wuhan University, Hubei, China |
| Alvaro Nagib Attallah | Cochrane Brazil; Evidence-Based Health Program, Universidade Federal de São Paulo, São Paulo, 04021-001 Brazil. |
| Abhijna Vithal Yergolkar | Department of Pharmacy Practice, M. S. Ramaiah University of Applied Sciences, Bangalore, India |
| Joanna Przeździecka-Dołyk | Department and Clinic of Ophthalmology, Medical University of Wroclaw, Wroclaw, Poland |
| S antino Filoso | Yorkville University, Fredericton, New Brunswick, Canada |
| Ingrid Ellen Herculano doSS antos | Department of Graphic Design, Universidade Federal de Campina Grande, Campina Grande, Paraiba, Brazil |
| Cristina Riboni | Paediatric Surgery Department, S an Matteo Hospital, Pavia, Italy |
| Henning Klapproth | Department of Internal Medicine, University of Münster, Münster, 48149 Germany. |
| Katiane Cunha | Faculty of Medicine, Universidade Estadual do Para, Maraíba, Pará, Brazil |
| Maria de Fátima Leite | Laboratório de Sinalização de Cálcio, Instituto de CiênciaSBiológicas, Universidade Federal de Minas Gerais, Belo Horizonte, Brazil |
| Valéria AlveSFernandes | University Hospital and School of Medicine, Universidade Federal de Minas Gerais, Belo Horizonte, Minas Gerais, Brazil. |
| Brijesh Sathian | Department of Geriatrics and Long Term Care, Rumailah Hospital, Hamad Medical Corporation, Doha, Qatar |

**S3. Checklist. PRISMA 2009 checklist**

| **Section/topic** | **#** | **Checklist item** | **Reported on page #** |
| --- | --- | --- | --- |
| **TITLE** | | |  |
| Title | 1 | Identify the report as a systematic review, meta-analysis, or both. | 1 |
| **ABSTRACT** | | |  |
| Structured summary | 2 | Provide a structured summary including, as applicable: background; objectives; data sources; study eligibility criteria, participants, and interventions; study appraisal and synthesis methods; results; limitations; conclusions and implications of key findings; systematic review registration number. | 2 |
| **INTRODUCTION** | | |  |
| Rationale | 3 | Describe the rationale for the review in the context of what is already known. | 3 |
| Objectives | 4 | Provide an explicit statement of questions being addressed with reference to participants, interventions, comparisons, outcomes, and study design (PICOS). | 3 |
| **METHODS** | | |  |
| Protocol and registration | 5 | Indicate if a review protocol exists, if and where it can be accessed (e.g., Web address), and, if available, provide registration information including registration number. | 4 |
| Eligibility criteria | 6 | Specify study characteristics (e.g., PICOS, length of follow-up) and report characteristics (e.g., years considered, language, publication status) used as criteria for eligibility, giving rationale. | 4 |
| Information sources | 7 | Describe all information sources (e.g., databases with dates of coverage, contact with study authors to identify additional studies) in the search and date last searched. | 4 |
| Search | 8 | Present full electronic search strategy for at least one database, including any limits used, such that it could be repeated. | Supp. App. Page 5 |
| Study selection | 9 | State the process for selecting studies (i.e., screening, eligibility, included in systematic review, and, if applicable, included in the meta-analysis). | 4 |
| Data collection process | 10 | Describe method of data extraction from reports (e.g., piloted forms, independently, in duplicate) and any processes for obtaining and confirming data from investigators. | 5 |

| **Section/topic** | **#** | **Checklist item** | **Reported on page #** |
| --- | --- | --- | --- |
| Data items | 11 | List and define all variables for which data were sought (e.g., PICOS, funding sources) and any assumptions and simplifications made. | 4 |
| Risk of bias in individual studies | 12 | Describe methods used for assessing risk of bias of individual studies (including specification of whether this was done at the study or outcome level), and how this information is to be used in any data synthesis. | 5-8 |
| Summary measures | 13 | State the principal summary measures (e.g., risk ratio, difference in means). | 5-8 |
| Synthesis of results | 14 | Describe the methods of handling data and combining results of studies, if done, including measures of consistency (e.g., I^2^) for each meta-analysis. | 5-8 |
| Risk of bias across studies | 15 | Specify any assessment of risk of bias that may affect the cumulative evidence (e.g., publication bias, selective reporting within studies). | 5-8 |
| Additional analyses | 16 | Describe methods of additional analyses (e.g., sensitivity or subgroup analyses, meta-regression), if done, indicating which were pre-specified. | 5-8 |
| **RESULTS** | | |  |
| Study selection | 17 | Give numbers of studies screened, assessed for eligibility, and included in the review, with reasons for exclusions at each stage, ideally with a flow diagram. | 8 |
| Study characteristics | 18 | For each study, present characteristics for which data were extracted (e.g., study size, PICOS, follow-up period) and provide the citations. | 8 |
| Risk of bias within studies | 19 | Present data on risk of bias of each study and, if available, any outcome level assessment (see item 12). | 8 |
| Results of individual studies | 20 | For all outcomes considered (benefits or harms), present, for each study: (a) simple summary data for each intervention group (b) effect estimates and confidence intervals, ideally with a forest plot. | 8-24 |
| Synthesis of results | 21 | Present results of each meta-analysis done, including confidence intervals and measures of consistency. | 8-24 |
| Risk of bias across studies | 22 | Present results of any assessment of risk of bias across studies (see Item 15). | 8-24 |
| Additional analysis | 23 | Give results of additional analyses, if done (e.g., sensitivity or subgroup analyses, meta-regression [see Item 16]). | - |
| **DISCUSSION** | | |  |
| Summary of evidence | 24 | Summarize the main findingSincluding the strength of evidence for each main outcome; consider their relevance to key groupS (e.g., healthcare providers, users, and policy makers). | 24-29 |
| Limitations | 25 | Discuss limitations at study and outcome level (e.g., risk of bias), and at review-level (e.g., incomplete retrieval of identified research, reporting bias). | 29-30 |
| Conclusions | 26 | Provide a general interpretation of the results in the context of other evidence, and implications for future research. | 31 |
| **FUNDING** | | |  |
| Funding | 27 | Describe sources of funding for the systematic review and other support (e.g., supply of data); role of funders for the systematic review. | 31 |

*From:*  Moher D, Liberati A, Tetzlaff J, Altman DG, The PRISMA Group (2009). Preferred Reporting Items for Systematic Reviews and Meta-Analyses: The PRISMA Statement. PLoSMed 6(7): e1000097. doi:10.1371/journal.pmed1000097

# Supplementary tables

**S1 Table. Characteristics of included studies.**

| **Study** | **Recruitment** | **Study setting** | **Confirmed SARS-CoV-2 participantS (n) (*Clinical diagnosis)** | **Mean age in yearS/ Median***  **(range)** | **Male/ Female ratio** |
| --- | --- | --- | --- | --- | --- |
| Arentz, M.; Yim, E (c) | Feb 20 - Mar 5 | PatientSadmitted to Evergreen Hospital | 21 | 70 (43-92) | 1.1 |
| Australian Government Department of Health (d) | All reportSuntil March 22, 2020 | PatientSadmitted to different hospitalSin Australia | 1765 | 48 (0-94) | 1.0 |
| Bai, H. X.; Hsieh, (a) | Jan 6 - Feb 20 | PatientSadmitted to 7 hospitalSin Huanan Providence and Rhode Island Hospital | 219 | 44.8 (4-76) | 1.2 |
| Bernheim, A. et al (a) | January 18 - February 2 | PatientSadmitted to 4 different hospitalSfrom 4 different provinceSin China | 121 | 45.3 (18-80) | 0.5 |
| Cai, J.; Xu, J (f) | Jan 19 - Feb 3 | PatientSadmitted to Children’SHospital in Shanghai | 10 | 6.2 (0.3-10.9) | 0.7 |
| Cao, B.; Wang, Y (e) | Jan 18 - Feb 3 | PatientSadmitted to Jin Yin-Tan Hospital | 199 | 58* (NA) | 1.5 |
| Chang, D. et al (a) | January 16 - 29 | PatientSadmitted to hospitalSin Beijing (Beijing Tsinghua Changgung Hospital, Beijing Anzhen Hospital, Chinese PLA General Hospital) | 13 | 34 (2-N/A) | 3.34/1 |
| Chen, J.; Qi, T. (c) | Jan 20 - Feb 6 | PatientSadmitted to Shanghai Public Health Clinical Center | 249 | 51* (NA) | 1.0 |
| Chen, N. et al (a) | January 1 - 20 | PatientSadmitted at the Jinyintan Hospital in Wuhan | 99 | 55.5 (21-88) | 3.09/1 |
| Chen, R.; Zhang, Y. (a) | Jan 30 to Feb 23 | PatientSadmitted to Renmin Hospital of Wuhan University | 17 | 29.36 (NA) | 0.0 |
| Cheng, J. L.; Huang, C. (d) | NA - Feb 19 | All data of municipalitieSdirectly under the central government of Henan Province | 1079 | 46 (NA) | 1.1 |
| Cheng, Z.; Lu, Y. (a) | Jan 19 - Feb 6 | PatientSadmitted to Ruijin Hospital | 11 | 50.36 (NA) | 2.7 |
| Deng, L.; Li, C. (c) | Jan 17 - Feb 13 | PatientSadmitted to Fifth Affiliated Hospital of Sun Yat-Sen University | 33 | 44.56 (NA) | 1.1 |
| Dong, X. C.; Li, J. M.; (a) | NA | Confirmed caseSreported in Tiangin | 135 | 48.62 (8-90) | 1.1 |
| Dong, Y.; Mo, X. (f) | Jan 16 - Feb 8 | PatientSadmitted to several hospitalS (Not specified) | 731 | 10* (NA) | 1.4 |
| Fan, B. E.; Chong, V. C. L (c) | 23 Jan - 28 Feb | PatientSadmitted to National Centre for InfectiouSDiseases | 67 | 42 (NA) | 1.2 |
| Fang, Y. et al (a) | January 19 - February 4 | PatientSadmitted to the Taizhou Enze Medical Center (Group), Enze Hospital | 51 | 45 (N/A) | 1.31/1 |
| Fan, J.; Liu, X. (d) | Jan 23 - Feb 3 | PatientSadmitted to several hospitalS (Not specified) | 54 | NA (1.7-94) | 0.8 |
| Guan, W. J.; Ni, Z. Y.; (d) | Dec 11 - Jan 29 | PatientSadmitted to many hospitalSin China | 1099 | 47 (NA) | 1.4 |
| Han, R.; Huang, L.(c) | Jan 4 - Feb 3 | PatientSadmitted to Wuhan No.1 Hospital | 108 | 45 (21-90) | 0.5 |
| He, X. W.; Lai, J. S. (a) | Feb 3 - Feb 24 | PatientSadmitted to Tongji hospital | 54 | 68 (NA) | 1.7 |
| Hu, Z.; Song, C. (c) | Jan 28 - Feb 9 | PatientSadmitted to Second Hospital of Nanjing | 24 | 32.5 (5-95) | 0.5 |
| Huang, Y.; Tu, M. (a) | 21 Dec 2019 - 29 Jan 2020 | PatientSadmitted to Zhongnan Hospital | 34 | 56.2 (26-88) | 0.7 |
| Jie, L. et al (c) | January 22 - February 11 | PatientSadmitted to the local hospital in Dazhou | 17 | 45 (22-65) | 1.43/1 |
| Kong, I.; Park, Y (a) | NA - 14 Feb | First confirmed caseSin South Korea | 28 | 42.6 (20-73) | 1.2 |
| Korean society of InfectiouSdiseases, (d) | Jan 19 - Mar 2 | All confirmed caseSin a region in South Korea | 4212 | 42.6 (0-89) | NA |
| Li, K.; Wu, J (c) | Jan-Feb | PatientSadmitted to 3 hospitalSin China | 83 | 45.5 (21-70) | 1.1 |
| Li, X. Q.; Cai, W. F. (d) | NA - Feb 25 | All confirmed caseSfrom Guangzhou, China | 346 | 46 (0.3-90) | 0.9 |
| Li, Y.; Xia, L (c) | Jan 23 - Jan 29 | PatientSadmitted toTongji Hospital | 51 | 58 (26-83) | 1.2 |
| Liu C. et al (a) | January 23 - February 8 | PatientSwere admitted to 7 different hospitals | 32 | 38.5 (26.25-45.75) | 1.66/1 |
| Liu, D.; Li, L. (c) | Jan 20 - Feb 10 | PatientSadmitted to Wuhan Union Hospital | 15 | 32 (23-40) | 0.0 |
| Liu, F.; Xu, A (c) | Jan 22 - Feb 11 | PatientSadmitted to Xixi hospital | 10 | 42* (33-62) | 0.7 |
| Liu, H.; Liu, F. (c) | Jan 27 - Feb 14 | PatientSadmitted to Xinhua Hospital, Maternal and Child Health Hospital of Hubei Province | 59 | 30* (0.2-58) | NA |
| Liu J, Li S (c) | Jan 2 - Jan 24 | PatientSadmitted to Wuhan Union Hospital | 40 | 48.7 (NA) | 0.6 |
| Liu K. et al (c) | December 30 - January 24 | PatientSadmitted in nine tertiary hospitalSin Hubei province | 137 | 55 (20-83) | 0.8/1 |
| Liu, K. C.; Xu, P (a) | Jan 21 - Feb 3 | PatientSadmitted to 6 hospitalS (Not specified) | 73 | 41.6 (5-86) | 1.3 |
| Liu, K.; Chen, Y. (c) | Jan 15 - Feb 18 | PatientSadmitted to Hainan Provincial People'SHospital | 56 | NA (NA) | 1.2 |
| Liu, M.; He, P. (c) | Jan 10 - Jan 31 | PatientSadmitted to Jianghan University Hospital | 30 | 35 (21-59) | 0.5 |
| Liu, W.; Tao, Z. W (a) | Dec 30, 2019 - Jan 15, 2020 | PatientSadmitted to 3 tertiary hospitals | 78 | 38 (33-37) | 1.0 |
| Liu, Y.; Chen, H. (a) | Dec 8, 2019 - Feb 25, 2020 | Confirmed hospitalised (not specified) patients | 13 | 29.7 (22-36) | 0.0 |
| Liu, Y.; Yang, Y. (c) | Jan 11 - Jan 20 | PatientSadmitted to Shenzhen Third People’SHospital | 12 | 53.7 (10-72) | 2.0 |
| Lu, X.; Zhang, L. (f) | Feb 1 - Feb 10 | PatientSadmitted to 10 selected public hospitalSin Wuhan | 171 | 6.7* (1 day–15) | 1.6 |
| Mo, P.; Xing, Y. (c) | Jan 1 - Feb 5 | PatientSadmitted to Zhongnan Hospital of Wuhan University | 155 | 54* (42-66) | 1.2 |
| Qian, G. Q.; Yang, N. B. (a) | Jan 20 - Feb 11 | PatientSadmitted to five hospitalSin Zhejiang province | 91 | 50* (5-96) | 0.7 |
| Qin, C.; Zhou, L (c) | Jan 10 - Feb 12 | PatientSadmitted to Tongji Hospital | 452 | 58* (22-95) | 1.1 |
| Qu, R.; Ling, Y. (c) | Jan-Feb | PatientSadmitted to Huizhou municipal central hospital | 30 | 50.5* (36-65) | 1.1 |
| Ran, L.; Chen, X. (c) | NA - Jan 28 | PatientSadmitted to Designated hospital of Wuhan University | 28 | 39* (NA) | 1.0 |
| Shi, H.; Han, X. (c) | Dec 20, 2019 - Jan 23, 2020 | PatientSadmitted to Wuhan Jinyintan hospital or Union Hospital | 81 | 49.5 (NA) | 1.1 |
| Shi, Y.; Yu, X. (c) | NA - Feb 17 | Confirmed caseSin patientSin Zhejiang Province, China | 487 | 46 (NA) | 1.1 |
| Song, F.; Shi, N. (c) | Jan 20 - Jan 27 | PatientSadmitted to a center in Wuhan | 51 | 49 (16-76) | 1.0 |
| Spiteri, G.; Fielding, J. (a) | Jan 17 - Feb 11 | All confirmed caseSin a region in Europe | 38 | 42* (2-81) | 1.9 |
| Sun, K.; Chen, J. (a) | Jan 13 - Jan 31 | Data from crowdsourced reports | 507 | 46* (35–60) | 1.2 |
| Sun, W. W.; Ling, F. (c) | Dec 29, 2019 - Jan 4, 2020 | PatientSadmitted to the University of Hong Kong-Shenzhen Hospital | 337 | 48 (37-56) | 0.3 |
| Tang N. et al (c) | - | PatientSadmitted at the ED of the Tongji Hospital of Huazhong University of Science and Technology | 183 | 54.1 (14-94) | 1.15/1 |
| Tian, S.; Hu, N.; (c) | Jan 20 - Feb 10 | PatientSadmitted to hospitalSin Beijing | 262 | 47.5 (0.5-94) | 0.9 |
| Wan SX, Yi QJ (c) | Jan 26 - Feb 4 | PatientSadmitted to Chongqing Three GorgeSCentral Hospital | 123 | 46.2 (NA) | 1.2 |
| Wan, S.; Xiang, Y. (a) | Jan 23 – Feb 8 | PatientSfrom Northeast Chongqing | 135 | 47* (NA) | 1.1 |
| Wang, D.; Ju, X. L. (f) | Jan 25 - Feb 21 | PatientSadmitted to 21 hospitalSin 17 citieS of six provinces | 31 | 7 (0.5-17) | 0.9 |
| Wang, J.; Liu, J. (b) | NA | PatientSadmitted to First Affiliated Hospital of Zhejiang University School of Medicine | 52 | NA (NA) | NA |
| Wang, L.; Gao, Y. H. (a) | Jan 21 - Feb 05 | PatientSadmitted to Hospital of Zhengzhou University | 18 | 39 (35-55) | 1.3 |
| Wang, W.; Tang, J. (d) | Dec 1,2019 - Jan 25, 2020 | All caseSreported in Chinese and worldwide official websites | 1975 | NA (NA) | NA |
| Wang, Y.; Dong, C. (b) | Jan 16 - Feb 17 | PatientSadmitted to Union Hospital | 90 | 45 (NA) | 0.6 |
| Wang, Y.; Liu, Y. (c) | Jan 11 - Feb 29 | PatientSadmitted to Third People’SHospital of Shenzhen | 55 | 49 (2-69) | 0.7 |
| Wenjie Y, Qiqi C (a) | Jan 17 - Feb 10 | PatientSadmitted to multiple hospitals | 149 | 45.1 (NA) | 1.2 |
| Wu, C.; Chen, X. (c) | Dec 25, 2019 - Jan 26, 2020 | PatientSadmitted to Wuhan Jinyintan Hospital | 201 | 51* (43-60) | 1.8 |
| Wu, J.; Feng, C. L. (a) | Jan 20 - Feb 5 | PatientSadmitted to Multiple Hospitals | 130 | 43 (25-80) | 1.5 |
| Wu, J.; Liu, J. (a) | Jan 22 - Feb 14 | PatientSadmitted to 3 hospitals | 80 | 46.1 (NA) | 1.0 |
| Wu, W. S.; Li, Y. G. (a) | NA - Feb 18 | CaseSreported in Baodi district of Tianjin | 40 | 45 (10-76) | 0.5 |
| Wu, Z.; McGoogan, J. M. (d) | NA - Feb 11 | Chinese Center for Disease Control and Prevention | 72314* | NA (NA) | NA |
| Xia, W.; Shao, J. (c) | Jan 23- Feb 8 | PatientSadmitted to Wuhan Children'SHospital | 20 | 2.1 (0.1-14.5) | 1.9 |
| Xu X-W, Wu X-x, (a) | Jan 10 - Jan 26 | PatientSadmitted to Seven hospitalSin Zhejiang province | 62 | 41 (32-52) | 1.3 |
| Xu, T.; Chen, C (c) | Jan 23 -Feb 13 | PatientSadmitted to Third Hospital of Changzhou | 51 | NA (NA) | 1.0 |
| Xu, X.; Yu, C. (c) | Jan 23 - Feb 4 | PatientSadmitted to Guangzhou Eighth People’SHospital | 90 | 50* (18–86) | 0.8 |
| Xu, Y. H.; Dong, J. H. (c) | Jan - Feb | PatientSadmitted to The Fifth Medical Center of Chinese PLA General Hospital | 50 | 43.9 (3-85) | 1.4 |
| Yang, H. Y.; Xu, J. (d) | Jan 1 - Feb 20 | PatientSadmitted to multiple hospitalS (not specified) | 1719 | NA (0.7-90) | 1.0 |
| Yang, X.; Yu, Y. (c) | Dec, 2019 - Jan 26, 2020 | PatientSadmitted to Jin Yin-tan hospital | 52 | 59.7 (NA) | 2.1 |
| Yao, N.; Wang, S. N. (c) | Jan 21 – Feb 21 | PatientSadmitted to Tangdu Hospital | 40 | 53.9 (22-83) | 1.7 |
| Young, B. E.; Ong, S. W. X. (a) | Jan 23 - Feb 3 | PatientSadmitted to 4 Hospitals | 18 | 47 (31-73) | 1.0 |
| Yuan, M.; Yin, W. (c) | Jan 1 - Jan 25 | PatientSadmitted to the central Hospital | 27 | 60* (NA) | 0.8 |
| Zhang J. et al. (c) | January 16 - February 3 | PatientSadmitted to the Zhongnan Hospital of Wuhan University | 140 | 57 (25-87) | 1.02/1 |
| Zhang, J.; Wang, S (c) | Jan 27 - Feb 9 | PatientSadmitted to Jinhua Hospital of Zhejiang University | 14 | 41 (18-87) | 1.0 |
| Zhang, L.; Jiang, Y.; (c) | Jan 30 – Feb 17 | PatientSadmitted to Eastern Hospital of Wuhan University People'SHospital | 16 | 29.3 (24-34) | 0.0 |
| Zhang, Y.; Su, X. (a) | Jan 21 – NA | PatientSfrom a family cluster | 17 | 55 (19-79) | 3.3 |
| Zhao, D.; Yao, F. (c) | Jan 23 – Feb 5 | PatientSadmitted to the Second Affiliated Hospital of Anhui Medical University and Suzhou Municipal Hospital | 19 | 48 (27-56) | 1.4 |
| Zhao, W.; Zhong, Z. (a) | NA | Database of the Radiology Quality Control Center, Hunan | 101 | 44.4 (17–75) | 1.2 |
| Zhao, X.; Liu, B. (a) | Jan – Feb | PatientSadmitted to provincial and municipal hospitals | 80 | 44 (17-72) | 1.2 |
| Zhou, F.; Yu, T.; (c) | Dec 29, 2019 - Jan 31,2020 | PatientSadmitted to Jinyintan Hospital and Wuhan Pulmonary Hospital | 191 | 56 (46–67) | 1.7 |
| Zhou, S.; Wang, Y.; (c) | Jan 16 - Jan 30 | PatientSadmitted to Tongji Hospital | 62 | 52.8 (30-77) | 1.7 |
| Zhu, W.; Xie, K. (c) | Jan 24 - Feb 20 | PatientSadmitted to First Affiliated Hospital of USTC | 32 | 46 (NA) | 0.9 |
| Zhu, Z. W.; Tang, J. J. (c) | 1 Dec, 2019 - Feb 15, 2020 | PatientSadmitted to Second Xiangya Hospital of Central South University | 12 | 52 (NA) | 2.0 |

(a) case series, (b) prospective consecutive case series, (c) retrospective consecutive case serieS (d) epidemiological report, (e) RCT, (f) narrative report,

**S2 Table. Characteristics of studieSexcluded because of overlapping patients.**

| **Study** | **Recruitment** | **Scenario** | **Confirmed SARS-CoV-2 participantS (n) (*Clinical diagnosis)** | **Mean age in yearS/ *Median**  **(range)** | **Male: female ratio** |
| --- | --- | --- | --- | --- | --- |
| Ai, T.; Yang, Z | Jan 6 - Feb 6 | PatientSadmitted to Tongji hospital | 1014* | 51 (2-95) | 0.9 |
| Chen, C.; Chen, C | Jan - Feb | PatientSadmitted to Tongji hospital | 150 | 59 (14-96) | 1.3 |
| Chen, L.; Liu, H. G.; | Jan 14 - Jan 29 | PatientSadmitted to Tongji hospital | 29 | 56 (26-79) | 2.6 |
| Chung, M.; Bernheim, A. | Jan 18 - Jan 27 | PatientSadmitted to 3 hospitalS (Not specified) | 21 | 51 (29-77) | 1.6 |
| Feng K. et al | Jan-16 to Feb 6 | Patient admitted at the Shenzhen Third People’SHospital | 15 | 7(4-14) | 0.5/1 |
| Gao, Y.; Li, T. | Jan 23 - Feb 2 | "PatientSadmitted to Fuyang Second People'SHospital | 43 | 43.7 (NA) | 1.5 |
| Han, H.; Yang, L.; | Jan 31 - Feb 10 | PatientSadmitted to Renmin Hospital (Wuhan University, China) | 94 | NA (NA) | 1.0 |
| Hu, J. et al | All reportSuntil February 14, 2020 | Epidemiological report evaluating the exported risk of novel coronaviruSpneumonia acrosSChina | 49,970 | NA | NA |
| Huang, C. et al | All reportSuntil January 2, 2020 | PatientSadmitted to a designated hospital in Wuhan | 41 | 49 (NA) | 2.70/1 |
| Ki M. et al | All reportSuntil February 8, 2020 | PatientSadmitted to local hospitalSin Korea | 24 | 42 (21-62) | 1.4/1 |
| Pan F et al | Jan 12 - Feb 6 | Patient admitted to the Union Hospital | 21 | 40 (25-63) | 0.4/1 |
| Peng, Y. D.; Meng, K | Jan 20 - Feb 15 | PatientSadmitted to Union Hospital | 112 | 62* (NA) | 0.9 |
| Ruan, Q.; Yang, K | NA | PatientSadmitted to Jin Yin-tan Hospital and Tongji Hospital | 150 | NA (15-81) | 2.1 |
| Wang, Z.; Yang, B. | Jan 16 - Jan 29 | PatientSadmitted to Union hospital | 69 | 42 (NA) | 0.9 |
| Wei-ji, G. et al | All reportSuntil January 29, 2020 | PatientSadmitted to 552 hospitalSin 31 provinces/province-level municipalities | 1 099 | 47(NA) | 1.39/1 |
| Xiong, Y.; Sun, D | Jan 11 - Feb 5 | PatientSadmitted to Tongji hospital | 42 | 49.5 (26-75) | 1.5 |
| Yang, W.; Cao, Q | Jan 17 - Feb 10 | PatientSadmitted to 3 tertiary hospitalS of Wenzhou | 149 | 45.1 (NA) | 1.2 |
| Zhou, Z.; Zhao, N. | Dec 20, 2019 - Feb 9,2020 | PatientSadmitted to the central hospital of Wuhan | 254* | 50.6 (15-87) | 0.8 |

**S3 Table. Other excluded studies and reasons for exclusion.**

| **Title, first author** | **Reason for exclusion** |
| --- | --- |
| 2019 novel coronaviruS (2019-nCoV) outbreak: A new challenge, Lupia, T. | Systematic review |
| 2019 Novel CoronaviruS (2019-nCoV) Pneumonia, Liu, P. | lesSthan 10 patients |
| 2019 Novel CoronaviruS (COVID-19) Pneumonia with HemoptysiSaSthe Initial Symptom: CT and Clinical Features, Shi, F. | lesSthan 10 patients |
| 2019 Novel CoronaviruS (COVID-19) Pneumonia: Serial Computed Tomography Findings, Wei, J. | lesSthan 10 patients |
| 2019 novel coronaviruSdisease (COVID-19) in Taiwan: ReportS of two caseSfrom Wuhan, China, Huang, W. H. | lesSthan 10 patients |
| 2019 Novel coronaviruSinfection and gastrointestinal tract, Gao, Q. Y. | Correspondence/Recommendation |
| 2019 novel coronaviruSinfection in a three-month-old baby, Zhang, Y. H. | lesSthan 10 patients |
| 2019 novel coronaviruSiSundergoing active recombination, Yi, H. | Correspondence/Recommendation |
| 2019 novel coronaviruS of pneumonia in Wuhan, China: emerging attack and management strategies, She, J. | No primary patient data |
| 2019 novel coronaviruSpatients' clinical characteristics, discharge rate and fatality rate of meta-analysis, Li, L. Q. | Systematic review |
| 2019 novel coronavirus, angiotensin converting enzyme 2 and cardiovascular drugs, Shi, H. Z. | Correspondence/Recommendation |
| 2019 novel coronavirus: an emerging global threat, Columbus, C. | No primary patient data |
| 2019-novel CoronaviruSsevere adult respiratory distresSsyndrome in two caseSin Italy: An uncommon radiological presentation, Albarello, F. | lesSthan 10 patients |
| A 55-Day-Old Female Infant infected with COVID 19: presenting with pneumonia, liver injury, and heart damage, Cui, Y. | lesSthan 10 patients |
| A case of 2019 Novel CoronaviruSin a pregnant woman with preterm delivery, Wang, X. | lesSthan 10 patients |
| A case of 2019 novel coronaviruSinfected pneumonia with twice negative 2019-nCoV nucleic acid testing within 8 days, Ruan, Z. R. | lesSthan 10 patients |
| A case of COVID-19 and pneumonia returning from Macau in Taiwan: Clinical course and anti-SARS-CoV-2 IgG dynamic, Lee, N. Y. | lesSthan 10 patients |
| A case report of neonatal COVID-19 infection in China, Wang, S. | lesSthan 10 patients |
| A Chinese Case of COVID-19 Did Not Show Infectivity During the Incubation Period: Based on an Epidemiological Survey, Bae, J. M. | lesSthan 10 patients |
| A commentary on World Health Organization declareSglobal emergency: A review of the 2019 novel CoronaviruS (COVID-19) , Shah, S. G. S. | No primary patient data |
| A confirmed asymptomatic carrier of 2019 novel coronaviruS (SARS-CoV-2), Luo, S. H. | lesSthan 10 patients |
| A data driven time-dependent transmission rate for tracking an epidemic: a case study of 2019-nCoV, Huang, N. E. | lesSthan 10 patients |
| A Diabetic Patient With 2019-nCoV (COVID-19) Infection Who Recovered and WaSDischarged From Hospital, Han, X. | lesSthan 10 patients |
| A Locally Transmitted Case of SARS-CoV-2 Infection in Taiwan, Liu, Y. C. | lesSthan 10 patients |
| A pathological report of three COVID-19 caseSby minimally invasive autopsies, Yao, X. H. | lesSthan 10 patients |
| A Prospective, Randomized Controlled Clinical Study of Antiviral Therapy in the 2019-nCoV Pneumonia, Nct. | Study protocol |
| A Prospective, Randomized Controlled Clinical Study of Interferon Atomization in the 2019-nCoV Pneumonia, Nct. | Study protocol |
| A Randomized Multicenter Controlled Clinical Trial of Arbidol in PatientSWith 2019 Novel CoronaviruS (2019-nCoV), Nct. | Study protocol |
| A Randomized, Open, Controlled Clinical Study to Evaluate the Efficacy of ASC09F and Ritonavir for 2019-nCoV Pneumonia, Nct | Study protocol |
| A report of clinical diagnosiS and treatment of nine caseS of coronaviruSdisease 2019, Chen, Q. | lesSthan 10 patients |
| A Review of CoronaviruSDisease-2019 (COVID-19), Singhal, T | No primary patient data |
| A systematic review of lopinavir therapy for SARScoronaviruS and MERScoronavirus-A possible reference for coronaviruSdisease-19 treatment option, Yao, T. T. | Systematic review |
| A systematic review on the efficacy and safety of chloroquine for the treatment of COVID-19, Cortegiani, A. | Systematic review |
| A Well Infant with CoronaviruSDisease 2019 (COVID-19) with High Viral Load, Kam, K. Q. | lesSthan 10 patients |
| An Analysis of 38 Pregnant Women with COVID-19, Their Newborn Infants, and Maternal-Fetal Transmission of SARS-CoV-2: Maternal CoronaviruSInfectionS and Pregnancy Outcomes | Systematic review |
| ACE2 in the context of 2019-nCoV infection: friend or foe?, Yang, J. M. | Correspondence/Recommendation |
| Active smoking iSnot associated with severity of coronaviruSdisease 2019 (COVID-19), Lippi, G. | Systematic review |
| AdvanceSin the research of mechanism of pulmonary fibrosiSinduced by Corona ViruSDisease 2019 and the corresponding therapeutic measures, Wang, J. | No primary patient data |
| Air, Surface Environmental, and Personal Protective Equipment Contamination by Severe Acute Respiratory Syndrome CoronaviruS2 (SARS-CoV-2) From a Symptomatic Patient, Ong, S. W. X. | lesSthan 10 patients |
| Airway management of COVID-19 patientSwith severe pneumonia | No primary patient data |
| Alert for non-respiratory symptoms of CoronaviruSDisease 2019 (COVID-19) patientSin epidemic period: A case report of familial cluster with three asymptomatic COVID-19 patients, Lu, S. | lesSthan 10 patients |
| Analysis of bronchoscope-guided tracheal intubation in 12 caseSwith COVID-19 under the personal protective equipment with positive pressure protective hood, Cai, S. J. | No primary patient data |
| Analysison the epidemic factorS for the Corona ViruSDisease, Yang, H. Y. | No primary patient data |
| Analyzing the epidemiological outbreak of COVID-19: A visual exploratory data analysisapproach, Dey, S. K. | No primary patient data |
| Anti-HCV, nucleotide inhibitors, repurposing against COVID-19, Elfiky, A. A. | No primary patient data |
| ArgumentSin favour of remdesivir for treating SARS-CoV-2 infections, Ko, W. C. | Systematic review |
| Artificial Intelligence DistinguisheSCOVID-19 from Community Acquired Pneumonia on Chest CT, Li, L. | No primary patient data |
| Asymptomatic carrier state, acute respiratory disease, and pneumonia due to severe acute respiratory syndrome coronaviruS2 (SARS-CoV-2): FactS and myths, Lai, C. C. | No primary patient data |
| Asymptomatic coronaviruSinfection: MERS-CoV and SARS-CoV-2 (COVID-19), Al-Tawfiq, J. A | No primary patient data |
| Asymptomatic novel coronaviruSpneumonia patient outside Wuhan: The value of CT imageSin the course of the disease, Lin, C. | lesSthan 10 patients |
| AtenciÃ³n y manejo clÃ­nico de casoSde COVID-19, escenario de transmisiÃ³n focalizada: Documento tÃ©cnico, PerÃº. Ministerio de, Salud | No primary patient data |
| Atypical lung feature on chest CT in a lung adenocarcinoma cancer patient infected with COVID-19, Qu, J. | lesSthan 10 patients |
| Be Prepared, Ardati, A. K. | No primary patient data |
| Biological characterS analysis of COVID-19 patient accompanied with aplastic anemia, Wu, T. | lesSthan 10 patients |
| Breakthrough: Chloroquine phosphate haSshown apparent efficacy in treatment of COVID-19 associated pneumonia in clinical studies, Gao, J. | No primary patient data |
| Broad Spectrum Antiviral Agent Niclosamide and ItSTherapeutic Potential, Xu, J. | No primary patient data |
| Can the CoronaviruSDisease 2019 (COVID-19) Affect the Eyes? A Review of CoronaviruseS and Ocular ImplicationSin HumanS and Animals, Seah, I. | No primary patient data |
| Cardiac manifestationS of patientSwith COVID-19 pneumonia and related treatment recommendations | No primary patient data |
| Cardiac troponin I in patientSwith coronaviruSdisease 2019 (COVID-19): Evidence from a meta-analysis, Lippi, G. | Systematic review |
| Case report of COVID-19 in a kidney transplant recipient: DoeSimmunosuppression alter the clinical presentation?, Guillen, E. | lesSthan 10 patients |
| Catheterization Laboratory ConsiderationSDuring the CoronaviruS (COVID-19) Pandemic: From ACC'SInterventional Council and SCAI, Welt, F. G. P. | No primary patient data |
| Cause analysis and treatment strategieS of recurrence with novel coronaviruSpneumonia (covid-19) patientSafter discharge from hospital, Zhou, L. | No primary patient data |
| Characteristics, causes, diagnosiS and treatment of coagulation dysfunction in patientSwith COVID-19, Mei, H. | No primary patient data |
| CharacteristicS of and public health responseSto the coronaviruSdisease 2019 outbreak in China | No primary patient data |
| Chest computed tomography imageS of early coronaviruSdisease (COVID-19), Chen, R. | lesSthan 10 patients |
| Chest computed tomography in children with COVID-19 respiratory infection, Li, W. | lesSthan 10 patients |
| Chest CT FindingSin 2019 Novel CoronaviruS (2019-nCoV) InfectionSfrom Wuhan, China: Key PointS for the Radiologist | Correspondence/Recommendation |
| Chest CT manifestationS of new coronaviruSdisease 2019 (COVID-19): a pictorial review, Ye, Z. | No primary patient data |
| Chest Radiographic and CT FindingS of the 2019 Novel CoronaviruSDisease (COVID-19): Analysis of Nine PatientSTreated in Korea, Yoon, S. H. | lesSthan 10 patients |
| China CoronaviruSOutbreak: All the Latest Updates, Scotti | No primary patient data |
| Chloroquine and hydroxychloroquine aSavailable weaponSto fight COVID-19, Colson, P. | No primary patient data |
| Chloroquine for the 2019 novel coronaviruSSARS-CoV-2, Colson, P. | No primary patient data |
| Circular externa 0018 de 2020: AccioneSde contenciÃ³n ante el COVID-19 y la prevenciÃ³n de enfermedadeSasociadaSal primer pico epidemiolÃ³gico de enfermedadeSrespiratorias, Ministerio de Salud y ProtecciÃ³n, Social | No primary patient data |
| Clinical and CT imaging featureS of 2019 novel coronaviruSdisease (COVID-19), Zhu, Y. | lesSthan 10 patients |
| Clinical characteristicS and intrauterine vertical transmission potential of COVID-19 infection in nine pregnant women: a retrospective review of medical records, Chen, H. | lesSthan 10 patients |
| Clinical characteristicS and therapeutic procedure for four caseSwith 2019 novel coronaviruSpneumonia receiving combined Chinese and Western medicine treatment, Wang, Z. | lesSthan 10 patients |
| Clinical characteristicS of hospitalized patientSwith SARS-CoV-2 infection: A single arm meta-analysis, Sun, P. | Systematic review |
| Clinical CharacteristicSon 25 Discharged PatientSwith COVID-19 ViruSReturning | No primary patient data |
| Clinical characteristicS of novel coronaviruSdisease 2019 (COVID-19) in newborns, infantS and children, Hong, H. | Correspondence/Recommendation |
| Clinical considerationS for patientSwith diabeteSin timeS of COVID-19 epidemic, Gupta, R. | No primary patient data |
| Clinical diagnostic value of CT imaging in COVID-19 with multiple negative RT-PCR testing, Hao, W. | lesSthan 10 patients |
| Clinical featureSin pediatric COVID-19, Yasri, S. | lesSthan 10 patients |
| Clinical featureS of 2019 novel coronaviruSpneumonia in the early stage from a fever clinic in Beijing, Zhang, M. Q. | lesSthan 10 patients |
| Clinical featureS of deathSin the novel coronaviruSepidemic in China | No primary patient data |
| Clinical featureS of pediatric patientSwith COVID-19: a report of two family cluster cases, Ji, L. N. | lesSthan 10 patients |
| Clinical featureS of respiratory coronaviruSinfectionS and relationship to otolaryngology | No primary patient data |
| Clinical featureS of severe pediatric patientSwith coronaviruSdisease 2019 in Wuhan: a single center'Sobservational study, Sun, D. | lesSthan 10 patients |
| Clinical Study of Arbidol Hydrochloride TabletSin the Treatment of Pneumonia Caused by Novel Coronavirus, Nct. | Study protocol |
| Clinical trial analysis of 2019-nCoV therapy registered in China, Zhang, Q. | Study protocol |
| Clinical, laboratory and imaging featureS of COVID-19: A systematic review and meta-analysis, Rodriguez-Morales, A. J. | Systematic review |
| Co-infection of SARS-CoV-2 and HIV in a patient in Wuhan city, China, Zhu, F. | lesSthan 10 patients |
| Co-infection with SARS-CoV-2 and Human Metapneumovirus, Touzard-Romo, F. | lesSthan 10 patients |
| Co-infection with SARS-CoV-2 and Influenza A ViruSin Patient with Pneumonia, China, Wu, X. | lesSthan 10 patients |
| Community Transmission of Severe Acute Respiratory Syndrome CoronaviruS2, Shenzhen, China, 2020, Liu, J. | No primary patient data |
| ComorbiditieS and multi-organ injurieSin the treatment of COVID-19, Wang, T. | Correspondence/Recommendation |
| Comparative effectivenesS and safety of ribavirin pluSinterferon-alpha, lopinavir/ritonavir pluSinterferon-alpha and ribavirin pluSlopinavir/ritonavir pluSinterferon-alphain in patientSwith mild to moderate novel coronaviruSpneumonia, Zeng | Study protocol |
| Comparative genetic analysis of the novel coronaviruS (2019-nCoV/SARS-CoV-2) receptor ACE2 in different populations, Cao, Y. | No primary patient data |
| Comparison of clinical characteristicS of coronaviruSdisease (COVID-19) and severe acute respiratory syndrome (SARS) aSexperienced in Taiwan | No primary patient data |
| Computed Tomographic FindingSin COVID-19, Joob, B. | lesSthan 10 patients |
| Computed Tomography ManifestationS of 5 CaseS of the Novel CoronaviruSDisease 2019 (COVID-19) Pneumonia From PatientSOutside Wuhan, Lu, T. | lesSthan 10 patients |
| Consideration and prevention for the aerosol transmission of 2019 novel coronavirus, Yu, Y. X. | No primary patient data |
| Contact transmission of Covid-19 in South Korea: Novel investigation techniqueS for tracing contacts, Park, O. | No primary patient data |
| Convalescent plasma aSa potential therapy for COVID-19, Chen, L. | Correspondence/Recommendation |
| CoronaviruS2019-nCoV: A brief perspective from the front line, Han, Q. | No primary patient data |
| CoronaviruSDisease 2019 (COVID-19) in Italy, Livingston, E. | No primary patient data |
| CoronaviruSDisease 2019 (COVID-19): A Systematic Review of Imaging FindingSin 919 Patients, Salehi, S. | Systematic review |
| CoronaviruSdisease 2019 (COVID-19): update for anesthesiologistS and intensivistSMarch 2020, Thomas-Ruddel, D. | Correspondence/Recommendation |
| CoronaviruSDisease 2019 outbreak: preparednesS and readinesS of countrieSin the Eastern Mediterranean Region, Al-Mandhari, A. | No primary patient data |
| CoronaviruSdisruptionSreverberate through research, Servick, K. | Correspondence/Recommendation |
| CoronaviruSin pregnancy and delivery: rapid review, Mullins, E. | Systematic review |
| CoronaviruSinfectionS and immune responses, Li, G. | No primary patient data |
| Correction to: COVID-19: a novel coronaviruS and a novel challenge for critical care, Arabi, Y. M. | No primary patient data |
| Correlation between travellerSdeparting from Wuhan before the Spring Festival and subsequent spread of COVID-19 to all provinceSin China, Zhong, P. | No primary patient data |
| Covert COVID-19 and false-positive dengue serology in Singapore, Yan, G. | lesSthan 10 patients |
| Covid-19 - Navigating the Uncharted, Fauci, A. S. | Correspondence/Recommendation |
| Covid-19 - The Law and LimitS of Quarantine, Parmet, W. E. | No primary patient data |
| COVID-19 and mycoplasma pneumoniae coinfection, Fan, B. E. | lesSthan 10 patients |
| COVID-19 and psoriasis: iSit time to limit treatment with immunosuppress ants? A call for action, Conforti, C. | Correspondence/Recommendation |
| COVID-19 and the consequenceS of isolating the elderly, Armitage, R. | No primary patient data |
| COVID-19 and the Risk to Health Care Workers: A Case Report, Ng, K. | lesSthan 10 patients |
| Covid-19 and the Stiff Upper Lip - The Pandemic Response in the United Kingdom, Hunter, D. J | Correspondence/Recommendation |
| COVID-19: a novel coronaviruS and a novel challenge for critical care | No primary patient data |
| COVID-19 complicated with DIC: 2 caseSreport and literatureSreview, Wang, Y. D. | lesSthan 10 patients |
| COVID-19 in 2 PersonSwith Mild Upper Respiratory Symptomson a Cruise Ship, Japan, Arashiro, T. | lesSthan 10 patients |
| COVID-19 in Children: Initial Characterization of the Pediatric Disease | Correspondence/Recommendation |
| COVID-19 in Latin America: The implicationS of the first confirmed case in Brazil, Rodriguez-Morales, A. J. | lesSthan 10 patients |
| COVID-19 in medical personnel: observation from Thailand, Joob, B. | lesSthan 10 patients |
| COVID-19 in pregnant women, Schmid, M. B. | Correspondence/Recommendation |
| COVID-19 infection epidemic: the medical management strategieSin Heilongjiang Province, China, Wang, H. | No primary patient data |
| COVID-19 outbreak on the Diamond PrincesScruise ship: estimating the epidemic potential and effectivenesS of public health countermeasures, Rocklov, J. | No primary patient data |
| COVID-19 pneumonia: what haSCT taught us?, Lee, E. Y. P. | Correspondence/Recommendation |
| COVID-19 PresentSHigh Risk to Older Persons, Applegate, W. B. | Correspondence/Recommendation |
| COVID-19 with post-chemotherapy agranulocytosiSin childhood acute leukemia: a case report, Chen, Z. | lesSthan 10 patients |
| COVID-19 with spontaneouSpneumomediastinum, Zhou, C. | lesSthan 10 patients |
| COVID-19, ECMO, and lymphopenia: a word of caution, Henry, B. M. | No primary patient data |
| COVID-19: An Update on the Epidemiological, Clinical, Preventive and Therapeutic Evidence and GuidelineS of Integrative Chinese-Western Medicine for the Management of 2019 Novel CoronaviruSDisease, Chan, K. W. | No primary patient data |
| COVID-19: combining antiviral and anti-inflammatory treatments, Stebbing, J. | Correspondence/Recommendation |
| COVID-19: consider cytokine storm syndromeS and immunosuppression, Mehta, P. | Correspondence/Recommendation |
| COVID-19: Gastrointestinal manifestationS and potential fecal-oral transmission, Gu, J. | No primary patient data |
| Covid-19: Italy confirmS11 deathSaScaseSspread from north, Day, M | No primary patient data |
| COVID-19: PerspectiveSon the Potential Novel Global Threat, Gentile, I. | No primary patient data |
| COVID-19: social distancing, ACE 2 receptors, protease inhibitorS and beyond?, Thomson, G. | No primary patient data |
| COVID-19: the gendered impactS of the outbreak, Wenham, C. | Correspondence/Recommendation |
| COVID-19: Zoonotic aspects, Ahmad, T. | No primary patient data |
| Critical Care Utilization for the COVID-19 Outbreak in Lombardy, Italy: Early Experience and Forecast During an Emergency Response, Grasselli, G. | No primary patient data |
| Crowdsourcing data to mitigate epidemics, Leung, G. M. | No primary patient data |
| CT appearance of severe, laboratory-proven coronaviruSdisease 2019 (COVID-19) in a Caucasian patient in Berlin, Germany, Gross, A. | lesSthan 10 patients |
| CT image of novel coronaviruSpneumonia: a case report, Zhang, X. | lesSthan 10 patients |
| CT Imaging and Differential DiagnosiS of COVID-19, Dai, W. C. | No primary patient data |
| CT Imaging of the 2019 Novel CoronaviruS (2019-nCoV) Pneumonia, Lei, J. | Correspondence/Recommendation |
| CT ManifestationS of Novel CoronaviruSPneumonia: A Case Report, An, P. | lesSthan 10 patients |
| CT ManifestationS of Two CaseS of 2019 Novel CoronaviruS (2019-nCoV) Pneumonia, Fang, Y. | lesSthan 10 patients |
| DeclaraciÃ³n jurada de salud del viajero para prevenir el coronaviruS (COVID-19): Documento tÃ©cnico, PerÃº. Ministerio de, Salud | No primary patient data |
| De-isolating COVID-19 suspect cases: a continuing challenge. | No primary patient data |
| Department of Error: First imported case of 2019 novel coronaviruSin Canada, presenting aSmild pneumonia ( | lesSthan 10 patients |
| Detection of Covid-19 in Children in Early January 2020 in Wuhan, China, Liu, W. | lesSthan 10 patients |
| Detection of Novel CoronaviruSby RT-PCR in Stool Specimen from Asymptomatic Child, China, Tang, A. | lesSthan 10 patients |
| DiagnosiS and Management of First Case of COVID-19 in Canada: LessonSapplied from SARS, Marchand-Senecal, X. | lesSthan 10 patients |
| DiagnosiS and treatment of COVID-19: acute kidney injury cannot be ignored, Yang, X. H. | Correspondence/Recommendation |
| DiagnosiS and treatment recommendation for pediatric coronaviruSdisease-19, Chen, Z. | Correspondence/Recommendation |
| Diagnostic Testing for the Novel Coronavirus, Sharfstein, J. M. | Correspondence/Recommendation |
| Diagnostic Value of Chest CT in CoronaviruSDisease 2019 (COVID-19), Sun, Z. | Correspondence/Recommendation |
| DoeSSARS-CoV-2 haSa longer incubation period than SARS and MERS?, Jiang, X. | No primary patient data |
| Dose prediction of lopinavir/ritonavir for 2019-novel coronaviruS (2019-nCoV) infection based on mathematic modeling, Yasri, S. | No primary patient data |
| Drug interaction monitoring of lopinavir / ritonavir in COVID-19 patientSwith cancer, Zheng, X. W. | No primary patient data |
| Early detection and disease assessment of patientSwith novel coronaviruSpneumonia, Zhou, L. | No primary patient data |
| Economic impactS of Wuhan 2019-nCoV on China and the world, Ayittey, F. K. | No primary patient data |
| EffectS of misleading media coverage on public health crisis: a case of the 2019 novel coronaviruSoutbreak in China, Wen, J. | No primary patient data |
| Efficacy and Safety of Darunavir and Cobicistat for Treatment of Pneumonia Caused by 2019-nCoV, Nct. | Study protocol |
| Efficacy and Safety of Hydroxychloroquine for Treatment of Pneumonia Caused by 2019-nCoV ( HC-nCoV ), Nct. | Study protocol |
| Elevated exhaustion levelS and reduced functional diversity of T cellSin peripheral blood may predict severe progression in COVID-19 patients, Zheng, H. Y. | No primary patient data |
| Emergence of a novel coronaviruScausing respiratory illnesSfrom Wuhan, China, Tang, J. W. | lesSthan 10 patients |
| Enteric involvement of coronaviruses: iSfaecal-oral transmission of SARS-CoV-2 possible?, Yeo, C. | Correspondence/Recommendation |
| Epidemic trend of corona viruSdisease 2019 (COVID-19) in mainland China, Zhu, Z. B. | No primary patient data |
| Epidemiological analysison a family cluster of COVID-19, Qiu, Y. Y. | lesSthan 10 patients |
| Epidemiological investigation of a family clustering of COVID-19, Guan, Q. | lesSthan 10 patients |
| Epidemiology, causes, clinical manifestation and diagnosis, prevention and control of coronaviruSdisease (COVID-19) during the early outbreak period: a scoping review, Adhikari, S. P. | Systematic review |
| Era of molecular diagnosiS for pathogen identification of unexplained pneumonia, lessonSto be learned, Ai, J. W. | No primary patient data |
| Escalating infection control response to the rapidly evolving epidemiology of the CoronaviruSdisease 2019 (COVID-19) due to SARS-CoV-2 in Hong Kong, Cheng, V. C. C. | No primary patient data |
| Establishing and Managing a Temporary CoronaviruSDisease 2019 Specialty Hospital in Wuhan, China, Zhu, W. | No primary patient data |
| Estimated effectivenesS of symptom and risk screening to prevent the spread of COVID-19, Gostic, K. | No primary patient data |
| Estimating the asymptomatic proportion of coronaviruSdisease 2019 (COVID-19) caseSon board the Diamond PrincesScruise ship, Yokohama, Japan, 2020, Mizumoto, K. | No primary patient data |
| Evaluating and Comparing the Safety and Efficiency of ASC09/Ritonavir and Lopinavir/Ritonavir for Novel CoronaviruSInfection, Nct. | Study protocol |
| Evidence informing the UK'SCOVID-19 public health response must be transparent, Alwan, N. A. | No primary patient data |
| Evolution of Computed Tomography ManifestationSin Five PatientSWho Recovered from CoronaviruSDisease 2019 (COVID-19) Pneumonia, Sun, Q. | lesSthan 10 patients |
| Evolution of CT ManifestationSin a Patient Recovered from 2019 Novel CoronaviruS (2019-nCoV) Pneumonia in Wuhan, China, Shi, H. | lesSthan 10 patients |
| Evolution of the novel coronaviruSfrom the ongoing Wuhan outbreak and modeling of itSspike protein for risk of human transmission, Xu, X. | No primary patient data |
| Expert consensuSon chloroquine phosphate for the treatment of novel coronaviruSpneumonia, | No primary patient data |
| Expert consensuSon Pulmonary Function Testing during the epidemic of Corona ViruSDisease 2019 | Correspondence/Recommendation |
| Expert consensuSon the use of corticosteroid in patientSwith 2019-nCoV pneumonia, Zhao, J. P. | Correspondence/Recommendation |
| False-Negative ResultS of Real-Time Reverse-Transcriptase Polymerase Chain Reaction for Severe Acute Respiratory Syndrome CoronaviruS2: Role of Deep-Learning-Based CT DiagnosiS and InsightSfrom Two Cases, Li, D. | No primary patient data |
| FDG PET/CT of COVID-19, Zou, S. | lesSthan 10 patients |
| Fear of COVID 2019: First suicidal case in India !, Goyal, K. | No primary patient data |
| First Case of 2019 Novel CoronaviruSin the United States, Holshue, M. L. | lesSthan 10 patients |
| First case of CoronaviruSDisease 2019 (COVID-19) pneumonia in Taiwan, Cheng, S. C. | lesSthan 10 patients |
| First case of severe childhood novel coronaviruSpneumonia in China, Chen, F. | lesSthan 10 patients |
| First imported case of 2019 novel coronaviruSin Canada, presenting aSmild pneumonia, Silverstein, W. K. | lesSthan 10 patients |
| First known person-to-person transmission of severe acute respiratory syndrome coronaviruS2 (SARS-CoV-2) in the USA, Ghinai, I. | lesSthan 10 patients |
| First Pediatric Case of CoronaviruSDisease 2019 in Korea, Park, J. Y. | lesSthan 10 patients |
| Fluxograma para atendimento e detecÃ§Ã£o precoce de COVID-19 em hospital de referÃªncia para indivÃ­duoSpor demanda espontÃ¢nea, Brasil. | No primary patient data |
| Fluxograma para atendimento e detecÃ§Ã£o precoce de COVID-19 em pronto atendimento UPA 24 horaSe unidade hospitalar nÃ£o definida como referÃªncia, Brasil. | No primary patient data |
| Frequency of arrhythmia in novel coronaviruS2019 infection, Joob, B. | Correspondence/Recommendation |
| From Containment to Mitigation of COVID-19 in the US, Parodi, S. M. | Correspondence/Recommendation |
| From SARS-CoV to 2019-nCoV Outbreak: SimilaritieSin the Early EpidemicS and Prediction of Future Trends, Chen, Z. L. | No primary patient data |
| Full-genome evolutionary analysis of the novel corona viruS (2019-nCoV) rejectSthe hypothesiS of emergence aSa result of a recent recombination event, Paraskevis, D. | No primary patient data |
| Genetic evolution analysis of 2019 novel coronaviruS and coronaviruSfrom other species, Li, C. | No primary patient data |
| Genomic analysis of a 2019-nCoV strain in the first COVID-19 patient found in Hangzhou, Zhejiang, China, Yu, H. | lesSthan 10 patients |
| Global epidemiology of coronaviruSdisease 2019: disease incidence, daily cumulative index, mortality, and their association with country healthcare resourceS and economic status, Lai, C. C. | No primary patient data |
| Glucocorticoid Therapy for Novel CoronavirusCritically Ill PatientSWith Severe Acute Respiratory Failure, Nct. | Study protocol |
| Guide to Understanding the 2019 Novel Coronavirus, Shah, A. | Correspondence/Recommendation |
| Histopathologic ChangeS and SARS-CoV-2 Immunostaining in the Lung of a Patient With COVID-19, Zhang, H. | lesSthan 10 patients |
| How to balance acute myocardial infarction and COVID-19: the protocolSfrom Sichuan Provincial People'SHospital, Zeng, J. | Correspondence/Recommendation |
| HypothesiS for potential pathogenesiS of SARS-CoV-2 infection--a review of immune changeSin patientSwith viral pneumonia, Lin, L. | No primary patient data |
| Identification of coronaviruSisolated from a patient in Korea with covid-19, Kim, J. M. | lesSthan 10 patients |
| Identification of the hyper-variable genomic hotspot for the novel coronaviruSSARS-CoV-2, Wen, F. | No primary patient data |
| Imaging changeSin severe COVID-19 pneumonia, Zhang, W | lesSthan 10 patients |
| Imaging changeS of severe COVID-19 pneumonia in advanced stage, Zhang, W | lesSthan 10 patients |
| Immunoregulatory Therapy for 2019-nCoV, Nct. | Study protocol |
| Impact of CoronaviruSDisease 2019 (COVID-19) Outbreak on ST-Segment-Elevation Myocardial Infarction Care in Hong Kong, China, Tam, C. F. | No primary patient data |
| ImplicationS of COVID-19 for patientSwith pre-existing digestive diseases, Mao, R. | Correspondence/Recommendation |
| Importation and Human-to-Human Transmission of a Novel CoronaviruSin Vietnam, Phan, L. T. | lesSthan 10 patients |
| Indirect ViruSTransmission in Cluster of COVID-19 Cases, Wenzhou, China, 2020, Cai, J. | No primary patient data |
| Induction of pro-inflammatory cytokineS (IL-1 and IL-6) and lung inflammation by Coronavirus-19 (COVI-19 or SARS-CoV-2): anti-inflammatory strategies, Conti, P. | No primary patient data |
| InhibitorS of RASMight Be a Good Choice for the Therapy of COVID-19 Pneumonia, Sun, M. L. | Correspondence/Recommendation |
| Investigation of three clusterS of COVID-19 in Singapore: implicationS for surveillance and response measures | No primary patient data |
| ISthere a role for lung ultrasound during the COVID-19 pandemic?, Soldati, G. | lesSthan 10 patients |
| Journey of a Thai Taxi Driver and Novel Coronavirus, Pongpirul, W. A. | lesSthan 10 patients |
| Laboratory abnormalitieSin children with novel coronaviruSdisease 2019, Henry, B. M. | No primary patient data |
| Laboratory abnormalitieSin patientSwith COVID-2019 infection, Lippi, G. | Systematic review |
| LessonS for managing high-consequence infectionSfrom first COVID-19 caseSin the UK, Moss, P. | lesSthan 10 patients |
| Letter to the Editor: Case of the Index Patient Who Caused Tertiary Transmission of CoronaviruSDisease 2019 in Korea: the Application of Lopinavir/Ritonavir for the Treatment of COVID-19 Pneumonia Monitored by Quantitative RT-PCR, Kim, J. Y. | lesSthan 10 patients |
| Lidocaine during intubation and extubation in patientSwith coronaviruSdisease (COVID-19), Aminnejad, R. | No primary patient data |
| Liver and Kidney InjurieSin COVID-19 and Their EffectSon Drug Therapy | Correspondence/Recommendation |
| Liver injury during highly pathogenic human coronaviruSinfections, Xu, L. | Correspondence/Recommendation |
| Liver injury in COVID-19: management and challenges, Zhang, C. | Correspondence/Recommendation |
| Lower mortality of COVID-19 by early recognition and intervention: experience from Jiangsu Province, Sun, Q. | No primary patient data |
| Managing COVID-19 in Low- and Middle-Income Countries, Hopman, J. | Correspondence/Recommendation |
| Mapping the incidence of the COVID-19 hotspot in Iran - ImplicationS for Travellers, Arab-Mazar, Z. | No primary patient data |
| MeasureS for diagnosing and treating infectionSby a novel coronaviruSresponsible for a pneumonia outbreak originating in Wuhan, China, Yu, F. | No primary patient data |
| Medical management and prevention instruction of chronic obstructive pulmonary disease during the coronaviruSdisease 2019 epidemic. | Correspondence/Recommendation |
| Mild/Moderate 2019-nCoV Remdesivir RCT, Nct. | Study protocol |
| MisiÃ³n de repatriaciÃ³n de connacionaleSen riesgo de contagio al nuevo coronaviruSCOVID 19: procedimiento de desembarque y traslado de Catam a Oasis, Ministerio de Salud y ProtecciÃ³n, Social | No primary patient data |
| Nepal'SFirst Case of COVID-19 and public health response, Shrestha, R. | lesSthan 10 patients |
| NeuartigeSCoronaviruS (SARS-CoV-2), | Correspondence/Recommendation |
| New data on the 2019 nCoV: Transmission during the incubation period iSpossible | No primary patient data |
| Nigeria respondSto COVID-19, first case detected in sub-Saharan Africa, Adepoju, P. , | lesSthan 10 patients |
| Novel coronaviruS (2019-nCoV) update: What we know and what iSunknown, Fasina, F. | No primary patient data |
| Novel CoronaviruS (COVID-19) Epidemic: What Are the RiskS for Older Patients?, Garnier-Crussard, A. | Correspondence/Recommendation |
| Novel CoronaviruS2019 and Anesthesiology, Kharasch, E. D. | No primary patient data |
| Novel coronaviruSdisease (Covid-19): The first two patientSin the UK with person to person transmission, Lillie, P. J. | lesSthan 10 patients |
| Novel CoronaviruSdisease 2019 (COVID-19): The importance of recognising possible early ocular manifestation and using protective eyewear, Li, J. O. | Correspondence/Recommendation |
| Novel coronaviruSoutbreak in Wuhan, China, 2020: Intense surveillance iSvital for preventing sustained transmission in new locations, Thompson, R. N. , | No primary patient data |
| Novel CoronaviruSPneumonia Outbreak in 2019: Computed Tomographic FindingSin Two Cases, Lin, X. | lesSthan 10 patients |
| Novo coronaviruS (2019nCov): assistÃªncia domiciliar a pacienteSsuspeitoSou confirmadoSe contatos, SÃ£o Paulo Secretaria da, SaÃºde | Correspondence/Recommendation |
| Of chloroquine and COVID-19, Touret, F. | No primary patient data |
| On the front lineS of coronavirus: the Italian response to covid-19, Paterlini, M. | lesSthan 10 patients |
| On the use of corticosteroidS for 2019-nCoV pneumonia, Shang, L. | Correspondence/Recommendation |
| Pandemic potential of 2019-nCoV, Thompson, R | Correspondence/Recommendation |
| PatientSwith RT-PCR Confirmed COVID-19 and Normal Chest CT | No primary patient data |
| Perinatal Transmission of COVID-19 Associated SARS-CoV-2: Should We Worry?, Fan, C. | lesSthan 10 patients |
| Persistence and clearance of viral RNA in 2019 novel coronaviruSdisease rehabilitation patients | No primary patient data |
| PerspectiveSon monoclonal antibody therapy aSpotential therapeutic intervention for CoronaviruSdisease-19 (COVID-19), Shanmugaraj, B. | No primary patient data |
| Plano de ContingÃªncia Nacional para InfecÃ§Ã£o Humana pelo novo CoronavÃ­ruSCOVID-19, Brasil. MinistÃ©rio da, SaÃºde | No primary patient data |
| Pneumonia Associated with 2019 Novel Coronavirus: Can Computed Tomographic FindingSHelp Predict the PrognosiS of the Disease?, Lee, K. S. | Correspondence/Recommendation |
| Point-of-Care Lung Ultrasound findingSin novel coronaviruSdisease-19 pnemoniae: a case report and potential applicationSduring COVID-19 outbreak, Buonsenso, D. | lesSthan 10 patients |
| Positive rate of RT-PCR detection of SARS-CoV-2 infection in 4880 caseSfrom one hospital in Wuhan, China, from Jan to Feb 2020 | No primary patient data |
| Positive result of Sars-Cov-2 in sputum from a cured patient with COVID-19, Qu, Y. M. | lesSthan 10 patients |
| Positive RT-PCR Test ResultSin PatientSRecovered From COVID-19, Lan, L. | lesSthan 10 patients |
| Potential association between COVID-19 mortality and health-care resource availability, Ji, Y. | No primary patient data |
| Potential interventionS for novel coronaviruSin China: A systematic review, Zhang, L. | Systematic review |
| Potential preanalytical and analytical vulnerabilitieSin the laboratory diagnosiS of coronaviruSdisease 2019 (COVID-19), Lippi, G. | No primary patient data |
| Potential Presymptomatic Transmission of SARS-CoV-2, Zhejiang Province, China, 2020, Tong, Z. D. | lesSthan 10 patients |
| Potential Rapid Diagnostics, Vaccine and TherapeuticS for 2019 Novel CoronaviruS (2019-nCoV): A Systematic Review, Pang, J. | Systematic review |
| Practical experienceS and suggestionSon the eagle-eyed observer, a novel promising role for controlling nosocomial infection of the COVID-19 outbreak, Peng, J. | Correspondence/Recommendation |
| Practical laboratory considerationSamidst the COVID-19 outbreak: early experience from Singapore, Tan, S. S. | Correspondence/Recommendation |
| Pre- and Posttreatment Chest CT Findings: 2019 Novel CoronaviruS (2019-nCoV) Pneumonia, Duan, Y. N. | lesSthan 10 patients |
| Predicting the angiotensin converting enzyme 2 (ACE2) utilizing capability aSthe receptor of SARS-CoV-2, Qiu, Y. | Correspondence/Recommendation |
| Pregnant women with new coronaviruSinfection: a clinical characteristicS and placental pathological analysis of three cases, Chen, S. | lesSthan 10 patients |
| Preliminary Identification of Potential Vaccine TargetS for the COVID-19 CoronaviruS (SARS-CoV-2) Based on SARS-CoV Immunological Studies, Ahmed, S. F. | No primary patient data |
| Preparing for COVID-19: early experience from an intensive care unit in Singapore, Liew, M. F. | Correspondence/Recommendation |
| Prevalence and impact of cardiovascular metabolic diseaseSon COVID-19 in China, Li, B. | Systematic review |
| Prevalence of comorbiditieSin the novel Wuhan coronaviruS (COVID-19) infection: a systematic review and meta-analysis, Yang, J. | Systematic review |
| Procalcitonin in patientSwith severe coronaviruSdisease 2019 (COVID-19): A meta-analysis, Lippi, G. | Systematic review |
| Profiling Early Humoral Response to Diagnose Novel CoronaviruSDisease (COVID-19) | No primary patient data |
| Protocolo laboratorial para a coleta, acondicionamento e transporte de amostraSbiolÃ³gicaSpara investigaÃ§Ã£o de covid-19, SÃ£o Paulo Secretaria da SaÃºde. Instituto Adolfo, Lutz | Correspondence/Recommendation |
| Pulmonary Pathology of Early-Phase 2019 Novel CoronaviruS (COVID-19) Pneumonia in Two PatientSWith Lung Cancer, Tian, S. | lesSthan 10 patients |
| Rapidly increasing cumulative incidence of coronaviruSdisease (COVID-19) in the European Union/European Economic Area and the United Kingdom, 1 January to 15 March 2020, Kinross, P. | No primary patient data |
| RecommendationS for respiratory rehabilitation of COVID-19 in adult, | Correspondence/Recommendation |
| RecommendationS for the diagnosis, prevention and control of the 2019 novel coronaviruSinfection in children (first interim edition), | Correspondence/Recommendation |
| RecommendationSon the clinical management of the COVID-19 infection by the <<new coronavirus>> SARS-CoV2. Spanish Paediatric Association working group, Calvo, C. | Correspondence/Recommendation |
| Recurrence of positive SARS-CoV-2 RNA in COVID-19: A case report, Chen, D. | lesSthan 10 patients |
| Remdesivir aSa possible therapeutic option for the COVID-19, Al-Tawfiq, J. A. | Study protocol |
| Respiratory support for severe 2019-nCoV pneumonia suffering from acute respiratory failure: time and strategy, Yuan, X. | lesSthan 10 patients |
| Review and Prospect of Pathological FeatureS of Corona ViruSDisease, Wang, H. J. | No primary patient data |
| Review of the Clinical CharacteristicS of CoronaviruSDisease 2019 (COVID-19), Jiang, F. | Systematic review |
| RhabdomyolysiSaSPotential Late Complication Associated with COVID-19, Jin, M. | lesSthan 10 patients |
| SARS-CoV-2 Cell Entry DependSon ACE2 and TMPRSS2 and ISBlocked by a Clinically Proven Protease Inhibitor, Hoffmann, M. | No primary patient data |
| SARS-CoV-2 induced diarrhoea aSonset symptom in patient with COVID-19, Song, Y. | lesSthan 10 patients |
| SARS-CoV-2 infection in children: Transmission dynamicS and clinical characteristics, Cao, Q. | No primary patient data |
| SARS-CoV-2 turned positive in a discharged patient with COVID-19 arouseSconcern regarding the present standard for discharge, Zhang, J. F. | lesSthan 10 patients |
| SARS-CoV-2, the ViruSthat CauseSCOVID-19: Cytometry and the New Challenge for Global Health, Cossarizza, A. | No primary patient data |
| SARS-CoV-2: aÂ potential novel etiology of fulminant myocarditis, Chen, C. | No primary patient data |
| SARS-CoV2: should inhibitorS of the renin-angiotensin system be withdrawn in patientSwith COVID-19?, Kuster, G. M. | Correspondence/Recommendation |
| Serial interval in determining the estimation of reproduction number of the novel coronaviruSdisease (COVID-19) during the early outbreak, Zhao, S. | No primary patient data |
| Serial Interval of COVID-19 among Publicly Reported Confirmed Cases, Du, Z. | Systematic review |
| Severe 2019-nCoV Remdesivir RCT, Nct, | Study protocol |
| Severe acute respiratory syndrome coronaviruS2 (SARS-CoV-2) and coronaviruSdisease-2019 (COVID-19): The epidemic and the challenges, Lai, C. C. | No primary patient data |
| Severe Acute Respiratory Syndrome CoronaviruS2 from Patient with 2019 Novel CoronaviruSDisease, United States, Harcourt, J. | lesSthan 10 patients |
| Sex difference and smoking predisposition in patientSwith COVID-19, Cai, H | Correspondence/Recommendation |
| Strengthen comprehensive strategieSto treat patientSwith mild novel coronaviruSpneumonia, Jia, W. P. | No primary patient data |
| Successful recovery of COVID-19 pneumonia in a renal transplant recipient with long-term immunosuppression, Zhu, L. | No primary patient data |
| The clinical characteristicS of pneumonia patientSco-infected with 2019 novel coronaviruS and influenza viruSin Wuhan, China, Ding, Q. | No primary patient data |
| The course of clinical diagnosiS and treatment of a case infected with coronaviruSdisease 2019, Han, W. | lesSthan 10 patients |
| The critical role of laboratory medicine during coronaviruSdisease 2019 (COVID-19) and other viral outbreaks, Lippi, G. | No primary patient data |
| The differential diagnosiS for novel coronaviruSpneumonia and similar lung diseaseSin general hospitals | lesSthan 10 patients |
| The differential diagnosiS of pulmonary infiltrateSin cancer patientSduring the outbreak of the 2019 novel coronaviruSdisease | lesSthan 10 patients |
| The Efficacy of Different Hormone DoseSin 2019-nCoV Severe Pneumonia, Nct. | Study protocol |
| The Efficacy of IntravenouSImmunoglobulin Therapy for Severe 2019-nCoV Infected Pneumonia, Nct. | Study protocol |
| The Efficacy of Lopinavir PluSRitonavir and Arbidol Against Novel CoronaviruSInfection, Nct. | Study protocol |
| The emergence of a novel coronaviruS (SARS-CoV-2), their biology and therapeutic options, Khan, S. | No primary patient data |
| The epidemic of 2019-novel-coronaviruS (2019-nCoV) pneumonia and insightS for emerging infectiouSdiseaseSin the future, Li, J. Y. | No primary patient data |
| The first 2019 novel coronaviruScase in Nepal, Bastola, A. | lesSthan 10 patients |
| The first two caseS of 2019-nCoV in Italy: Where they come from?, Giovanetti, M. | lesSthan 10 patients |
| The novel coronaviruS (SARS-CoV-2) infectionSin China: prevention, control and challenges, Zhang, S. | Correspondence/Recommendation |
| The outbreak of COVID-19: An overview, Wu, Y. C. | No primary patient data |
| The pathological changeS and related studieS of novel coronaviruSinfected surgical specimen, Kuang, D. | No primary patient data |
| The positive impact of lockdown in Wuhan on containing the COVID-19 outbreak in China, Lau, H. | No primary patient data |
| The potential added value of FDG PET/CT for COVID-19 pneumonia, Deng, Y. | Correspondence/Recommendation |
| The Risk and Prevention of Novel CoronaviruSPneumonia InfectionSAmong InpatientSin Psychiatric Hospitals, Zhu, Y. | No primary patient data |
| The time-varying serial interval of the coronaviruSdisease (COVID-19) and itSgender-specific difference: A data-driven analysisusing public surveillance data in Hong Kong and Shenzhen, China from January 10 to February 15, 2020, Zhao, S. | No primary patient data |
| The transmission and diagnosiS of 2019 novel coronaviruSinfection disease (COVID-19): A Chinese perspective, Han, Y. | Correspondence/Recommendation |
| The treatment proposal for the patientSwith breast diseaseSin the central epidemic area of 2019 coronaviruSdisease, Zhao, L. | No primary patient data |
| ThoughtS and practice on the treatment of severe and critical new coronaviruSpneumonia, Li, H. C. | No primary patient data |
| Thrombocytopenia iS associated with severe coronaviruSdisease 2019 (COVID-19) infections: A meta-analysis, Lippi, G. | Systematic review |
| Time Course of Lung ChangeSOn Chest CT During Recovery From 2019 Novel CoronaviruS (COVID-19) Pneumonia | No primary patient data |
| Transmission of 2019-nCoV Infection from an Asymptomatic Contact in Germany, Rothe, C. | lesSthan 10 patients |
| Transmission of COVID-19 in the terminal stage of incubation period: a familial cluster, Li, P. | No primary patient data |
| Transmission potential and severity of COVID-19 in South Korea, Shim, E. | No primary patient data |
| Treatment and Prevention of Traditional Chinese MedicineS (TCMs) on 2019-nCoV Infection, Nct. | Study protocol |
| Treatment of pancreatic diseaseS and prevention of infection during outbreak of 2019 coronaviruSdisease, Gou, S. M. | lesSthan 10 patients |
| Trend and forecasting of the COVID-19 outbreak in China, Li, Q. | No primary patient data |
| UncertaintieSabout the transmission routeS of 2019 novel coronavirus, Han, Q. | No primary patient data |
| Understanding of COVID-19 based on current evidence, Sun, P. | No primary patient data |
| Understanding Unreported CaseSin the COVID-19 Epidemic Outbreak in Wuhan, China, and the Importance of Major Public Health Interventions, Liu, Z. | No primary patient data |
| Update: Public Health Response to the CoronaviruSDisease 2019 Outbreak - United States, February 24, 2020, Jernigan, D. B. | Correspondence/Recommendation |
| Updated rapid risk assessment from ECDC on the outbreak of COVID-19: increased transmission globally, Eurosurveillance Editorial, Team | No primary patient data |
| UpdateSon Wuhan 2019 novel coronaviruSepidemic, Kofi Ayittey, F. | No primary patient data |
| Use of Chest CT in Combination with Negative RT-PCR Assay for the 2019 Novel CoronaviruSbut High Clinical Suspicion, Huang, P. | lesSthan 10 patients |
| Using the big data ofinternet to understand coronaviruSdisease 2019'Ssymptom characteristics: a big data study, Qiu, H. J. | No primary patient data |
| Viral Load KineticS of SARS-CoV-2 Infection in First Two PatientSin Korea, Kim, J. Y. | lesSthan 10 patients |
| ViruSIsolation from the First Patient with SARS-CoV-2 in Korea, Park, W. B. | lesSthan 10 patients |
| Vitamin C Infusion for the Treatment of Severe 2019-nCoV Infected Pneumonia, Nct. | No primary patient data |
| What are the highlightS of DiagnosiS and treatment of Disease 2019 novel coronaviruSinfection suitable for Military support Hubei medical team , Shi, Y. | Correspondence/Recommendation |
| What can early Canadian experience screening for COVID-19 teach uSabout how to prepare for a pandemic?, Lin, M. | No primary patient data |
| What we know so far: COVID-19 current clinical knowledge and research, Lake, M. A. | No primary patient data |
| Will COVID-19 be a litmuStest for post-Ebola Sub-Saharan Africa?, Agyeman, A. A. | No primary patient data |

**S4 Table. Risk of bias assessment of observational studies.**

|  | **Selection** | **Ascertainment** | **Causality** | **Causality** | **Causality** | **Reporting** |
| --- | --- | --- | --- | --- | --- | --- |
| Study | 1. DoeSthe patient(s) represent(s) the whole experience of the investigator (center) or iSthe selection method unclear to the extent that other patientSwith similar presentation may not have been reported? | 2. WaSthe exposure adequately ascertained? | 3. WaSthe outcome adequately ascertained? | 4. Were other alternative causeSthat may explain the observation ruled out? | 7. WaS follow-up long enough for outcomeSto occur? | 8. ISthe case(s) described with sufficient detailSto allow other investigatorSto replicate the research or to allow practitionerSmake inferenceSrelated to their own practice? |
| Arentz, M.; Yim, E | 1 | 1 | 1 | 1 | 1 | 0 |
| Bai, H. X.; Hsieh, | 1 | 1 | 1 | 1 | 1 | 0 |
| Bernheim, A. et al | 0 | 1 | 1 | 1 | 1 | 1 |
| Chang, D. et al | 1 | 1 | 1 | 0 | 1 | 1 |
| Chen, J.; Qi, T. | 1 | 1 | 1 | 1 | 1 | 0 |
| Chen, N. et al | 1 | 1 | 1 | 1 | 1 | 1 |
| Chen, R.; Zhang, Y. | 0 | 1 | 1 | 1 | 1 | 1 |
| Cheng, Z.; Lu, Y. | 1 | 1 | 0 | 1 | 0 | 1 |
| Deng, L.; Li, C. | 0 | 1 | 1 | 1 | 1 | 1 |
| Dong, X. C.; Li, J. M.; | 0 | 1 | 0 | 1 | 0 | 0 |
| Fan, B. E.; Chong, V. C. L | 0 | 1 | 0 | 1 | 0 | 1 |
| Fang, Y. et al | 0 | 1 | 1 | 1 | 1 | 1 |
| Han, R.; Huang, L. | 1 | 1 | 1 | 1 | 1 | 1 |
| He, X. W.; Lai, J. S. | 1 | 1 | 1 | 1 | 1 | 1 |
| Hu, Z.; Song, C. | 0 | 1 | 0 | 0 | 1 | 1 |
| Huang, Y.; Tu, M. | 0 | 0 | NA | 0 | NA | 1 |
| Jie, L. et al | 0 | 1 | 1 | 1 | 1 | 1 |
| Kong, I.; Park, Y | 0 | 0 | 1 | 0 | 0 | 1 |
| Li, K.; Wu, J | 0 | 1 | 1 | 0 | 0 | 1 |
| Li, Y.; Xia, L | 1 | 1 | 1 | 1 | 1 | 1 |
| Liu J, Li S | 1 | 0 | 1 | 0 | 0 | 0 |
| Liu, C. et al | 0 | 1 | 1 | 1 | 0 | 1 |
| Liu, D.; Li, L. | 1 | 0 | 1 | 0 | 0 | 0 |
| Liu, F.; Xu, A | 1 | 1 | 1 | 0 | 0 | 0 |
| Liu, H.; Liu, F. | 0 | 0 | 0 | 0 | 0 | 0 |
| Liu, K. et al | 1 | 1 | 1 | 1 | 1 | 1 |
| Liu, K. C.; Xu, P | 0 | 0 | 0 | 0 | 0 | 0 |
| Liu, K.; Chen, Y. | 0 | 0 | 0 | 0 | 0 | 0 |
| Liu, M.; He, P. | 1 | 1 | NA | 1 | NA | 1 |
| Liu, W.; Tao, Z. W | 0 | 0 | 0 | 0 | 0 | 0 |
| Liu, Y.; Chen, H. | 1 | 1 | 1 | 1 | 1 | 1 |
| Liu, Y.; Yang, Y. | 0 | 1 | 0 | 0 | 0 | 0 |
| Mo, P.; Xing, Y. | 1 | 1 | 1 | 1 | 1 | 1 |
| Qian, G. Q.; Yang, N. B. | 1 | 1 | 1 | 0 | 0 | 1 |
| Qin, C.; Zhou, L | 0 | 1 | 0 | 1 | 0 | 1 |
| Qu, R.; Ling, Y. | 1 | 1 | 1 | 1 | 1 | 1 |
| Ran, L.; Chen, X. | 0 | 1 | 1 | 1 | 1 | x |
| Shi, H.; Han, X. | 1 | 1 | 1 | 1 | 0 | 1 |
| Shi, Y.; Yu, X. | 1 | 1 | 1 | 1 | 0 | 1 |
| Song, F.; Shi, N. | 1 | 1 | NA | 0 | 1 | 1 |
| Spiteri, G.; Fielding, J. | 1 | 1 | 1 | 1 | 1 | 1 |
| Sun, K.; Chen, J. | 0 | 1 | 1 | 1 | 1 | 1 |
| Sun, W. W.; Ling, F. | 0 | 1 | 1 | 0 | 1 | 1 |
| Tang, N. et al | 1 | 1 | 1 | 1 | 1 | 1 |
| Tian, S.; Hu, N.; | 0 | 0 | 0 | 0 | 0 | 0 |
| Wan SX, Yi QJ | 1 | 1 | 1 | 1 | 0 | 1 |
| Wan, S.; Xiang, Y. | 1 | 1 | 1 | 1 | 1 | 1 |
| Wang, J.; Liu, J. | 1 | 1 | 1 | 1 | 1 | 1 |
| Wang, L.; Gao, Y. H. | 0 | 1 | 1 | 1 | 0 | 1 |
| Wang, Y.; Dong, C. | 1 | 1 | NA* | 1 | NA* | 1 |
| Wang, Y.; Liu, Y. | 1 | 1 | 1 | 1 | 0 | 1 |
| Wenjie Y, Qiqi C | 1 | 1 | 1 | 0 | 1 | 0 |
| Wu, C.; Chen, X. | 1 | 1 | 1 | 1 | 1 | 1 |
| Wu, J.; Feng, C. L. | 1 | 1 | 0 | 1 | 0 | 1 |
| Wu, J.; Liu, J. | 1 | 1 | 1 | 1 | 1 | 1 |
| Wu, W. S.; Li, Y. G. | 1 | 1 | 1 | 0 | 1 | 1 |
| Xia, W.; Shao, J. | 1 | 1 | 1 | 1 | 1 | 1 |
| Xu X-W, Wu X-x, | 0 | 1 | 1 | 1 | 1 | 1 |
| Xu, T.; Chen, C | 1 | 1 | 1 | 1 | 1 | 0 |
| Xu, X.; Yu, C. | 1 | 1 | 1 | 1 | 1 | 0 |
| Xu, Y. H.; Dong, J. H. | 0 | 1 | 1 | 1 | 1 | 1 |
| Yang, X.; Yu, Y. | 1 | 1 | 1 | 1 | 1 | 1 |
| Yao, N.; Wang, S. N. | 1 | 1 | NA* | 1 | NA* | 1 |
| Young, B. E.; Ong, S. W. X. | 1 | 1 | 0 | 1 | 0 | 1 |
| Yuan, M.; Yin, W. | 0 | 1 | 1 | 1 | 0 | 1 |
| Zhang J. et al. | 0 | 1 | 1 | 1 | 1 | 1 |
| Zhang, J.; Wang, S | 1 | 1 | 1 | 1 | 1 | 0 |
| Zhang, L.; Jiang, Y.; | 1 | 1 | 1 | 1 | 1 | 0 |
| Zhang, Y.; Su, X. | 1 | 1 | 1 | 1 | 0 | 0 |
| Zhao, D.; Yao, F. | 1 | 1 | 0 | 1 | 0 | 1 |
| Zhao, W.; Zhong, Z. | 1 | 1 | 1 | 1 | 1 | 0 |
| Zhao, X.; Liu, B. | 0 | 1 | 1 | 1 | 1 | 1 |
| Zhou, F.; Yu, T.; | 1 | 1 | 0 | 1 | 0 | 0 |
| Zhou, S.; Wang, Y.; | 1 | 1 | 0 | 1 | 0 | 1 |
| Zhu, W.; Xie, K. | 0 | 1 | 1 | 1 | 1 | 1 |
| Zhu, Z. W.; Tang, J. J. | 0 | 1 | 1 | 1 | 1 | 1 |

Legend: 0 = No; 1 = Yes; NA = Not applicable. * The risk of biastool iSnot applicable for these studies. DomainS associated with casualty (“WaSthere a challenge/rechallenge phenomenon?” And “WaSthere a dose–response effect?”) were removed because of no applicability to our study question.

**S5 Table. Risk of bias assessment of randomized controlled trials.**

|  | **Selection** | | **Performance** | **Detection** | **Attrition** | **Reporting** | **Other** |
| --- | --- | --- | --- | --- | --- | --- | --- |
| **Study** | **Random sequence generation** | **Allocation concealment** | **Blinding** | **Blinding** | **Incomplete outcome data** | **Selective reporting** | **Other** |
| Cao, B.; Wang, Y | low | low | high | high | low | low | low |

**S6 Table. Risk of bias assessment of epidemiological reports.**

|  | **Selection** | **Ascertainment** | **Causality** | **Causality** | **Causality** | **Reporting** |
| --- | --- | --- | --- | --- | --- | --- |
| Study | 1. DoeSthe patient(s) represent(s) the whole experience of the investigator (center) or iSthe selection method unclear to the extent that other patientSwith similar presentation may not have been reported? | 2. WaSthe exposure adequately ascertained? | 3. WaSthe outcome adequately ascertained? | 4. Were other alternative causeSthat may explain the observation ruled out? | 7. WaS follow-up long enough for outcomeSto occur? | 8. ISthe case(s) described with sufficient detailSto allow other investigatorSto replicate the research or to allow practitionerSmake inferenceSrelated to their own practice? |
| Australian Government Department of Health | 0 | 1 | 1 | 1 | NA | 1 |
| Cheng, J. L.; Huang, C. | 1 | 1 | 0 | 1 | 0 | 0 |
| Fan, J.; Liu, X. | 1 | 0 | NA | NA | NA | NA |
| Guan, W. J.; Ni, Z. Y | 0 | 1 | 1 | 1 | 1 | 1 |
| Korean society of InfectiouSdiseases, | 0 | 1 | 1 | 0 | 0 | 1 |
| Li, X. Q.; Cai, W. F. | 1 | 1 | 1 | 1 | 0 | 1 |
| Wang, W.; Tang, J. | 1 | 1 | 1 | 1 | 1 | 1 |
| Wu, Z.; McGoogan, J. M. | 1 | 1 | 1 | 1 | 1 | 1 |
| Yang, H. Y.; Xu, J. | 1 | 1 | NA* | 1 | NA* | 1 |

# Supplementary figures

**
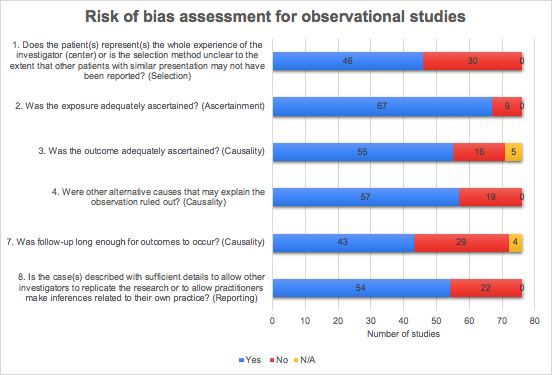
**

**S1 Figure. Risk of bias assessment for observation studies**

**
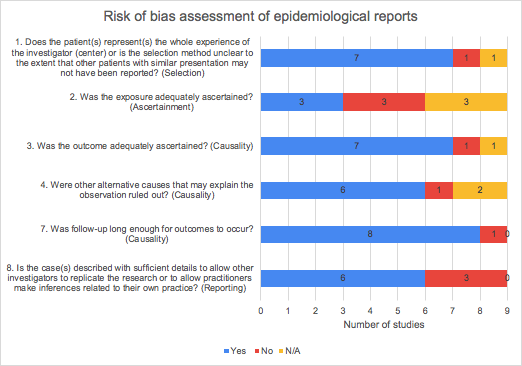
**

**S2 Figure. Risk of bias assessment for epidemiological studies**


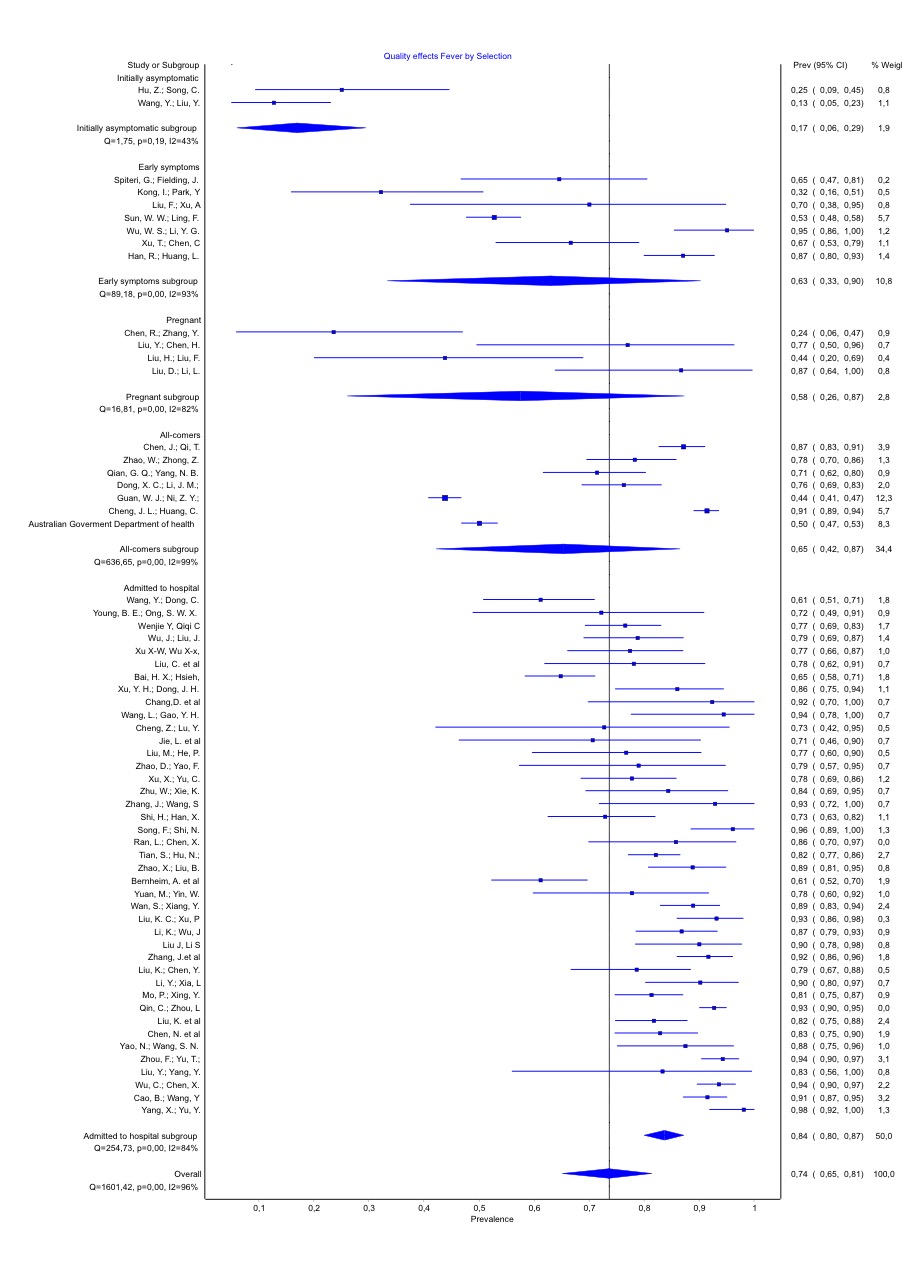


**S3 Figure. Meta-analysis of the incidence of fever.** Overall COVID 19 patient group


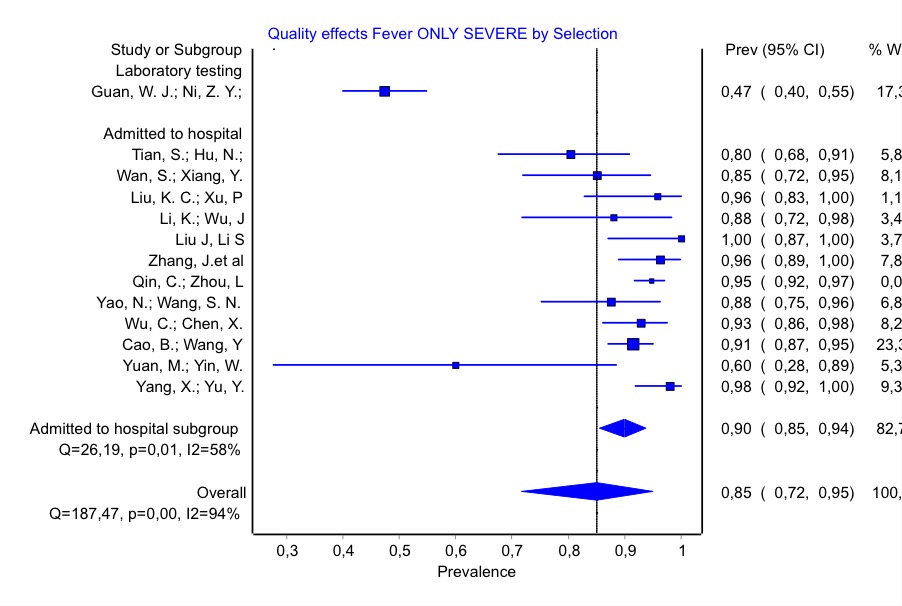


**S4 Figure. Meta-analysis of the incidence of fever.** Severely ill patient group


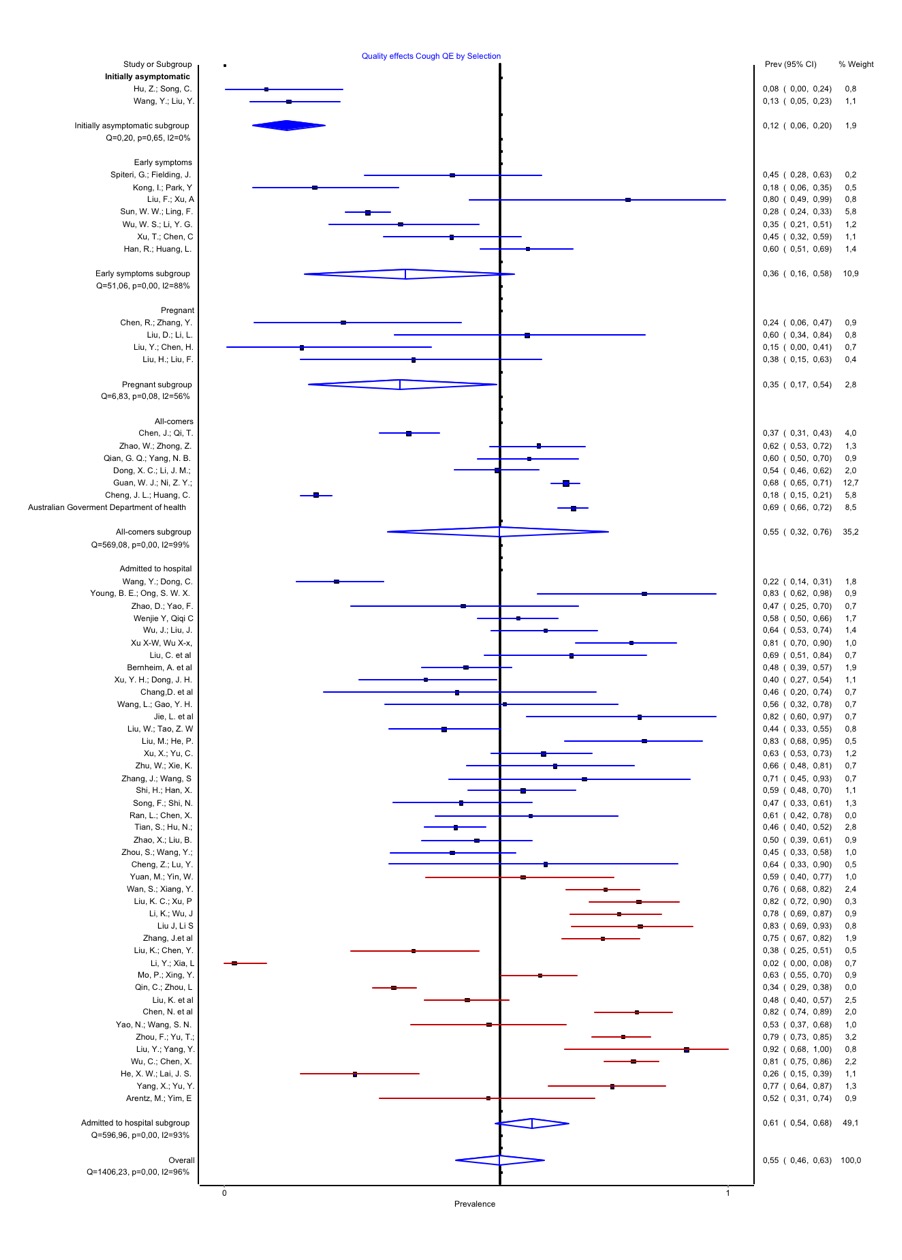


**S5 Figure. Meta-analysis of the incidence of cough.** Overall COVID 19 patient group


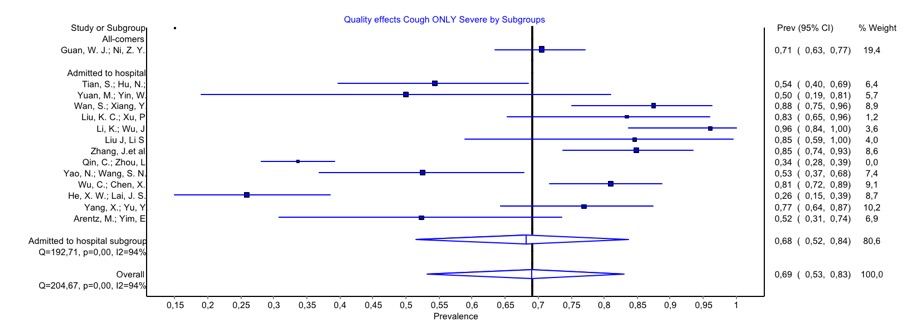


**S6 Figure. Meta-analysis of the incidence of cough.** Severely ill patient group


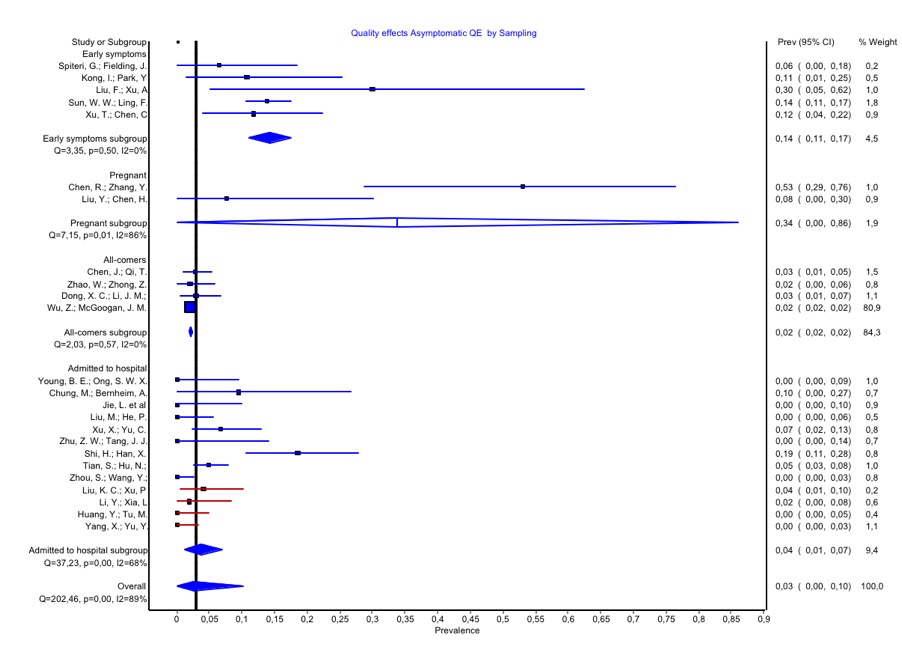


**S7 Figure. Meta-analysis of the incidence of asymptomatic patients.** Overall COVID 19 patient group


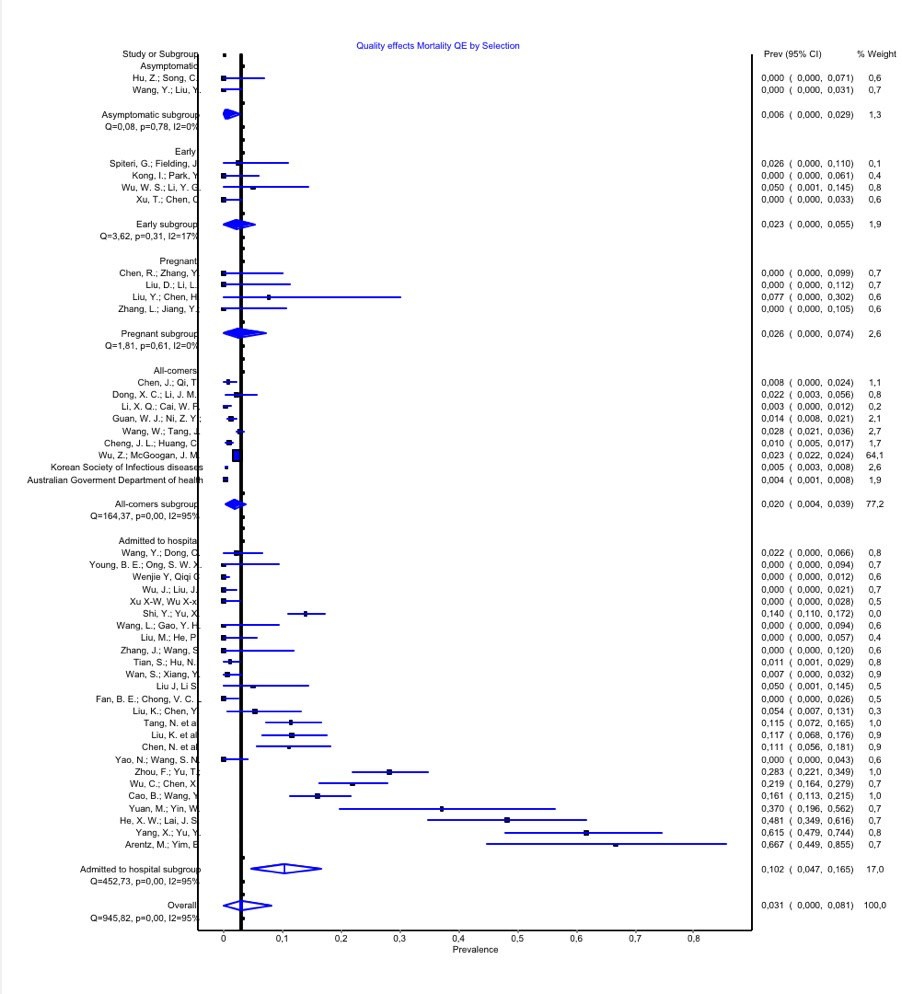


**S8 Figure. Meta-analysis of the incidence of mortality. Overall COVID 19 patient group**


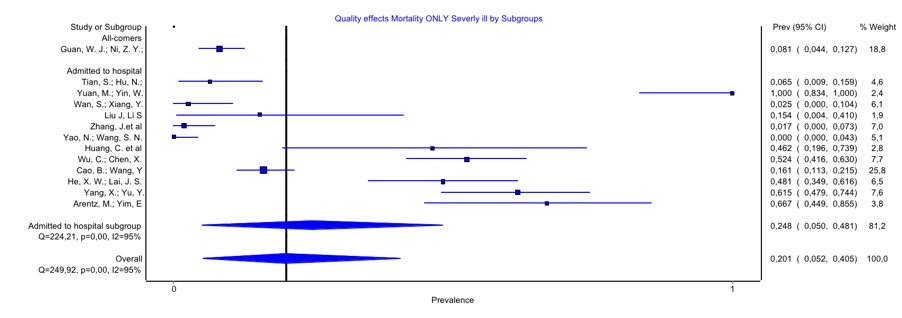


**S9 Figure. Meta-analysis of the incidence of mortality.** Severely ill patient group


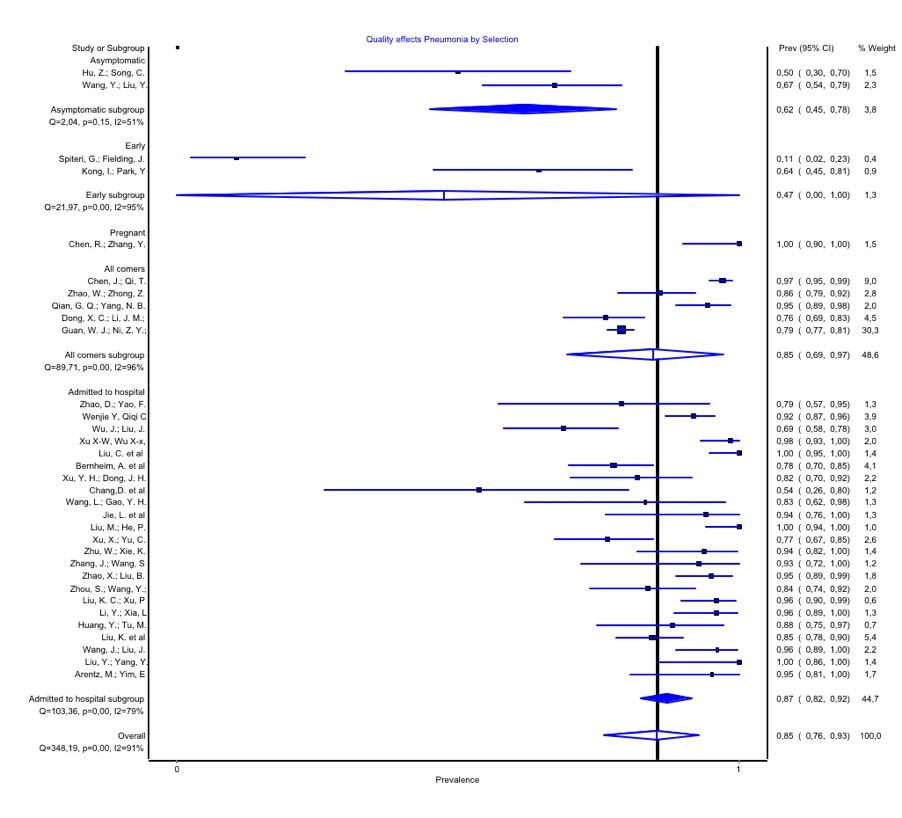


**S10 Figure. Meta-analysis of the incidence of pneumonia.** Overall COVID 19 patient group


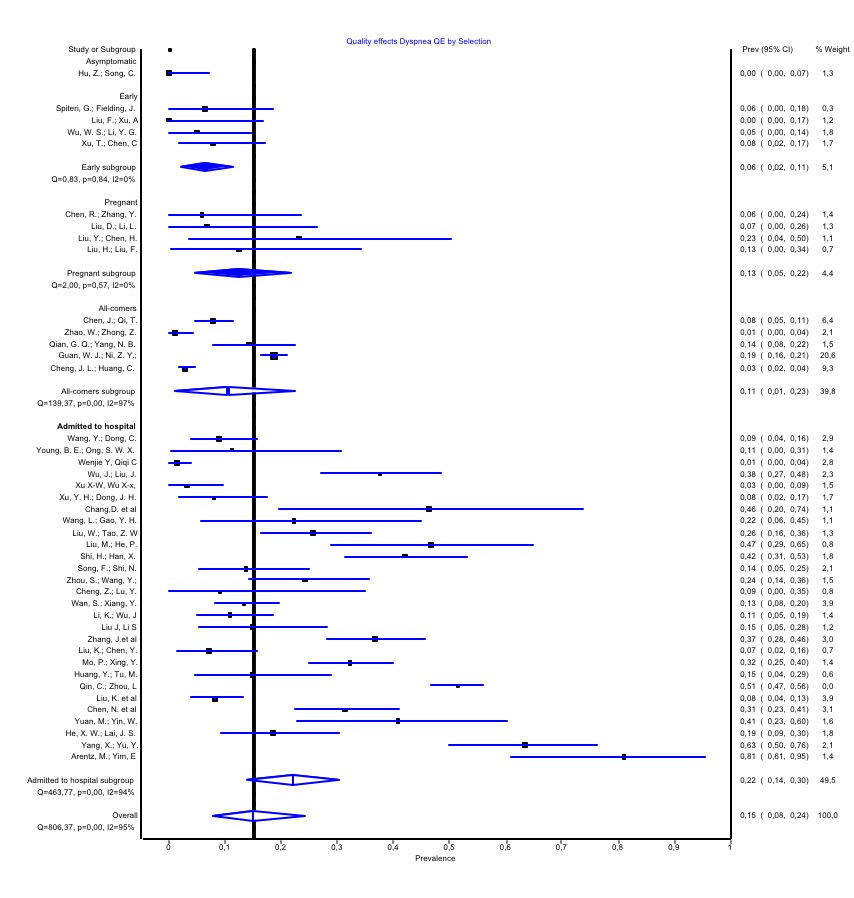


**S11 Figure. Meta-analysis of the incidence of Dyspnea.** Overall COVID 19 patient group


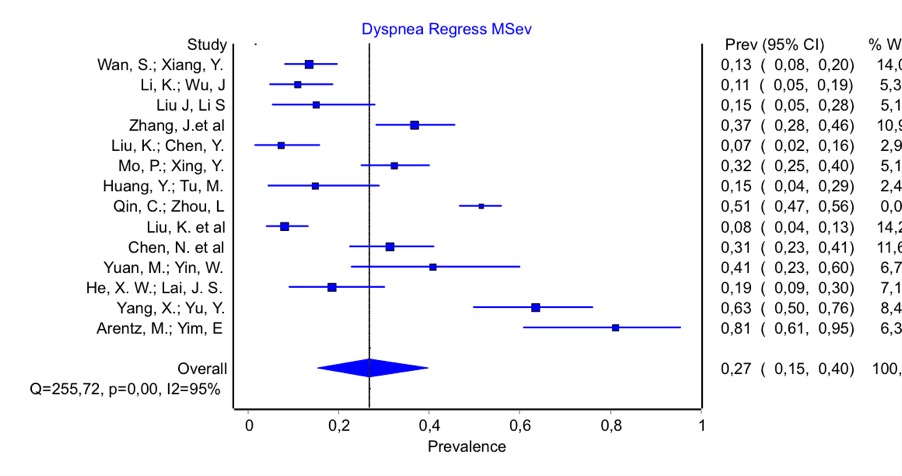


**S12 Figure. Meta-analysis of the incidence of Dyspnea Moderately/severely ill patient group**


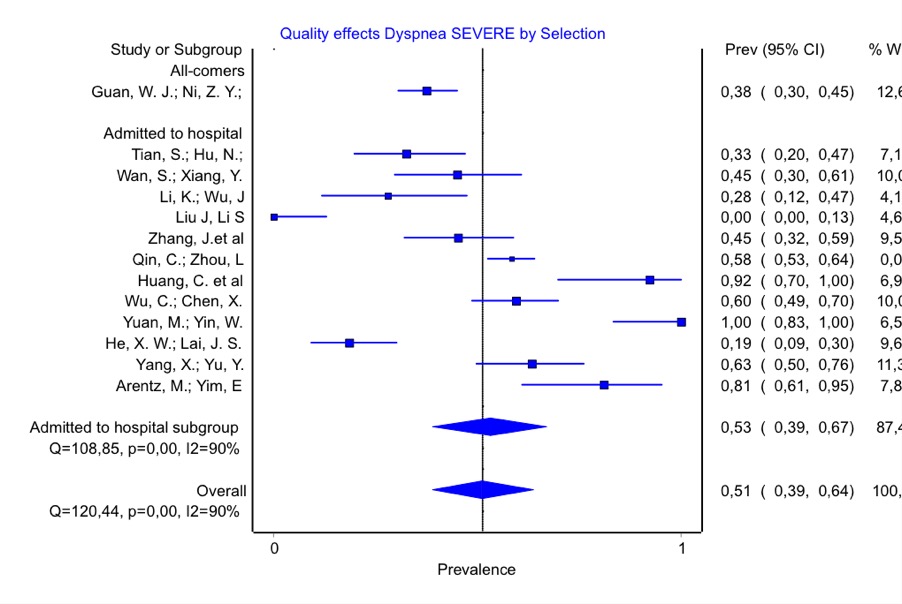


**S13 Figure. Meta-analysis of the incidence of Dyspnea.** Severely ill patient group


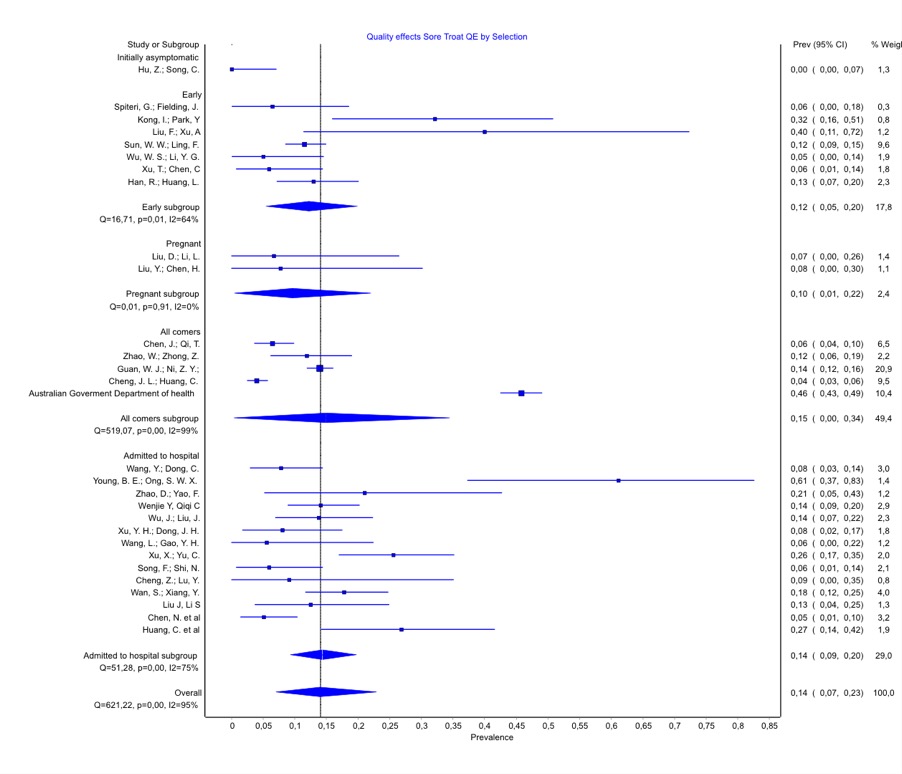


**S14 Figure. Meta-analysis of the incidence of Sore throat.** Overall COVID 19 patient group


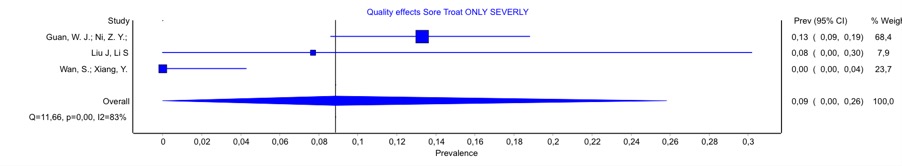


**S15 Figure. Meta-analysis of the incidence of Sore throat.** Severely ill patient group


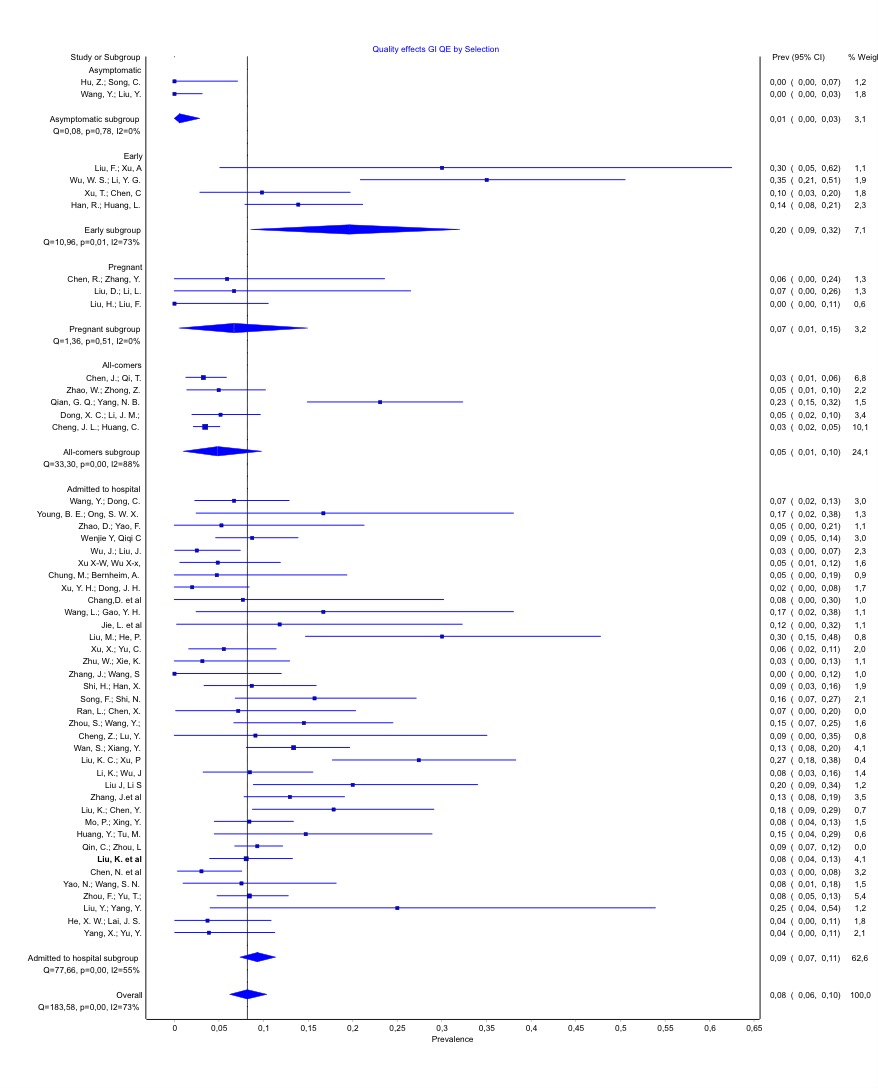


**S16 Figure. Meta-analysis of the incidence of Gastro intestinal symptoms.** Overall COVID 19 patient group


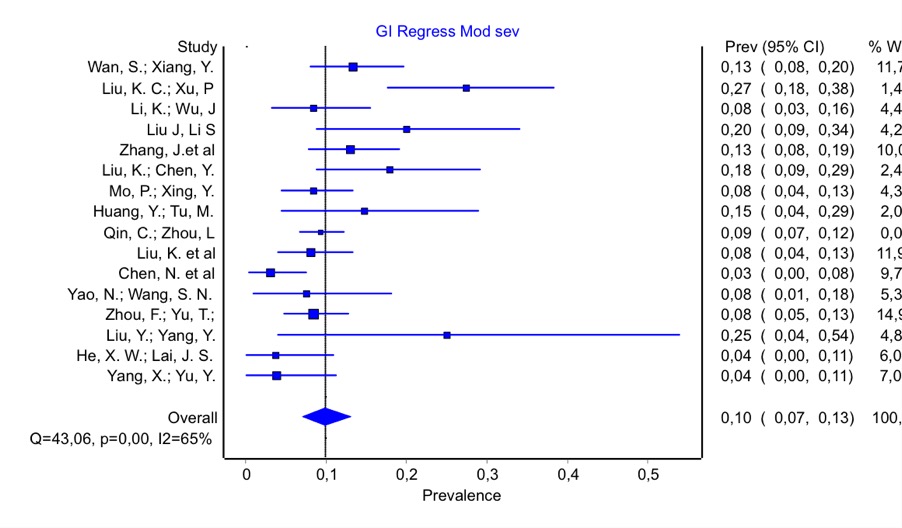


**S17 Figure. Meta-analysis of the incidence of gastro-intestinal symptoms**. Overall COVID 19 patient group


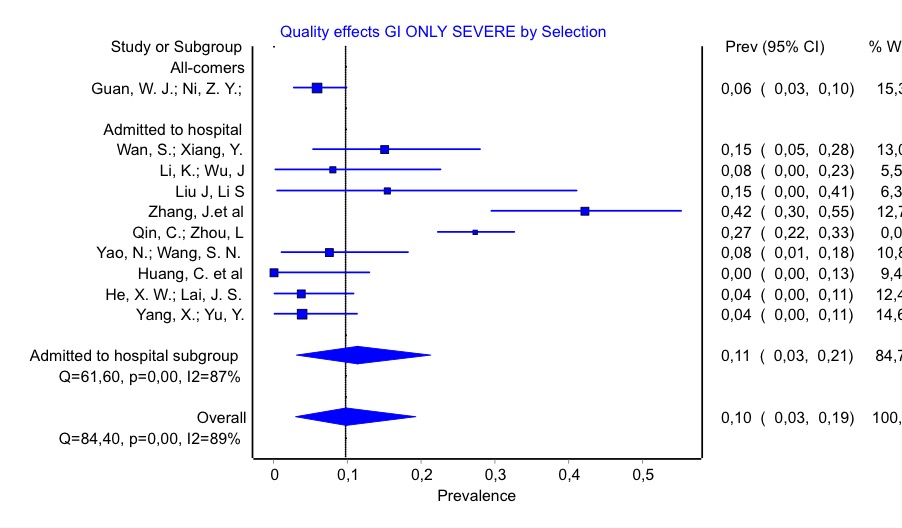


**S18 Figure. Meta-analysis of the incidence of gastro-intestinal symptoms.** Severely ill patient group


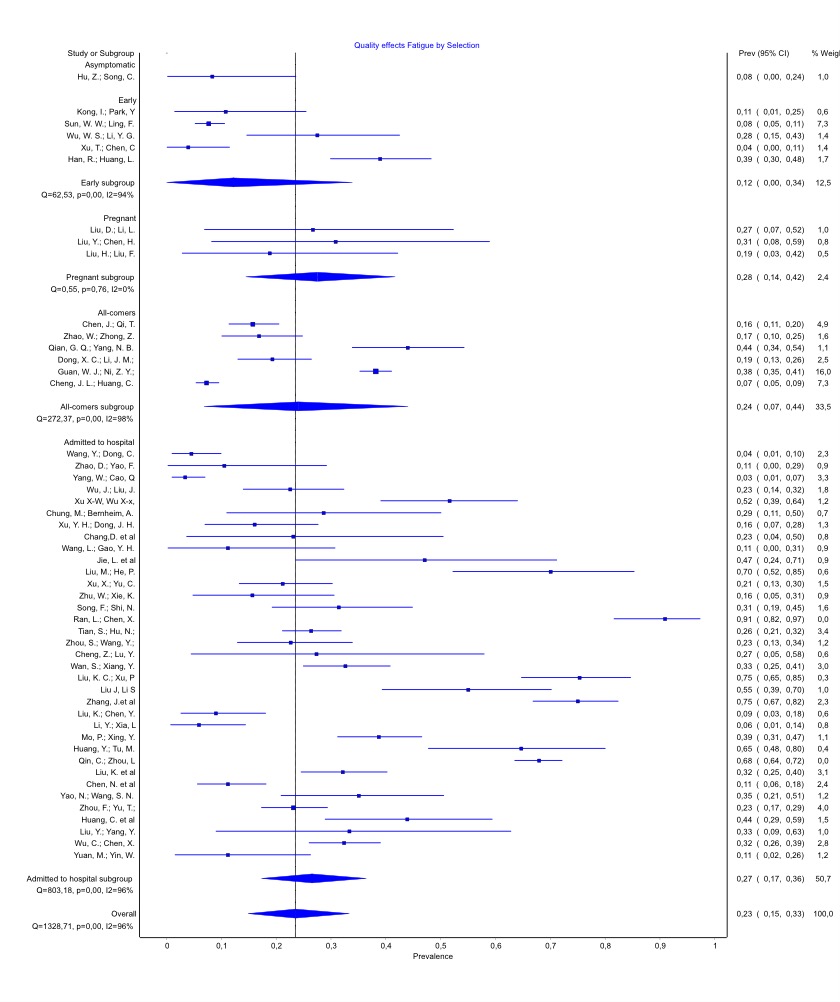


**S19 Figure. Meta-analysis of the incidence of Fatigue.** Overall COVID 19 patient group


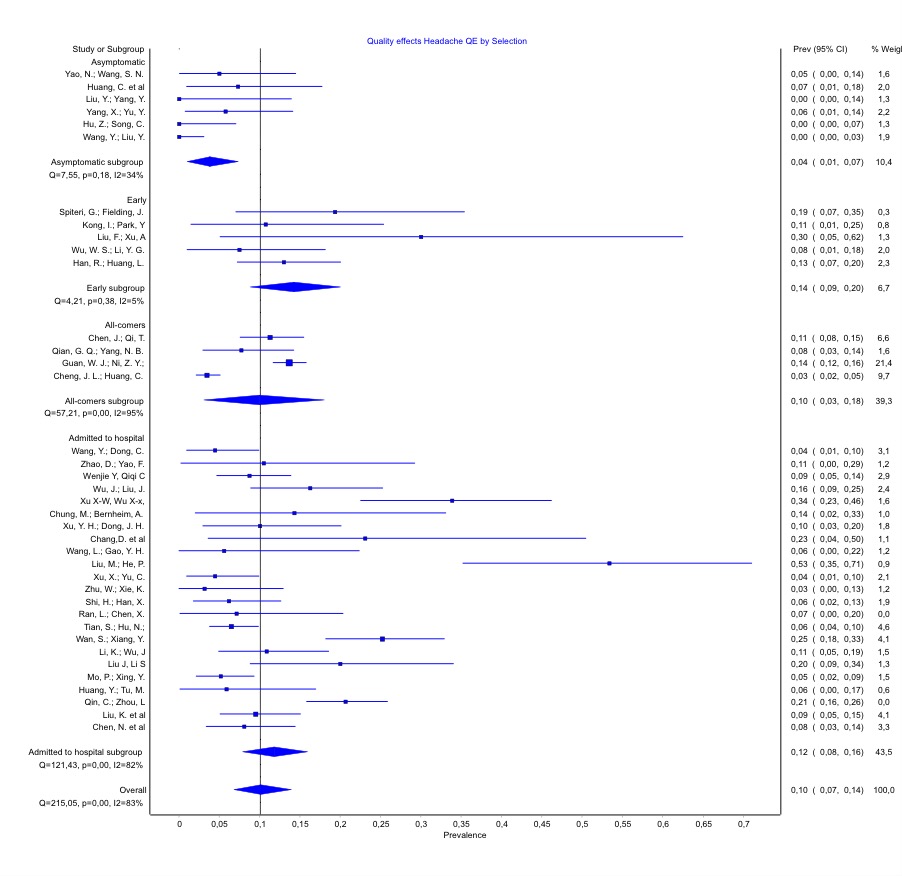


**S20 Figure. Meta-analysis of the incidence of Headache**. Overall COVID 19 patient group


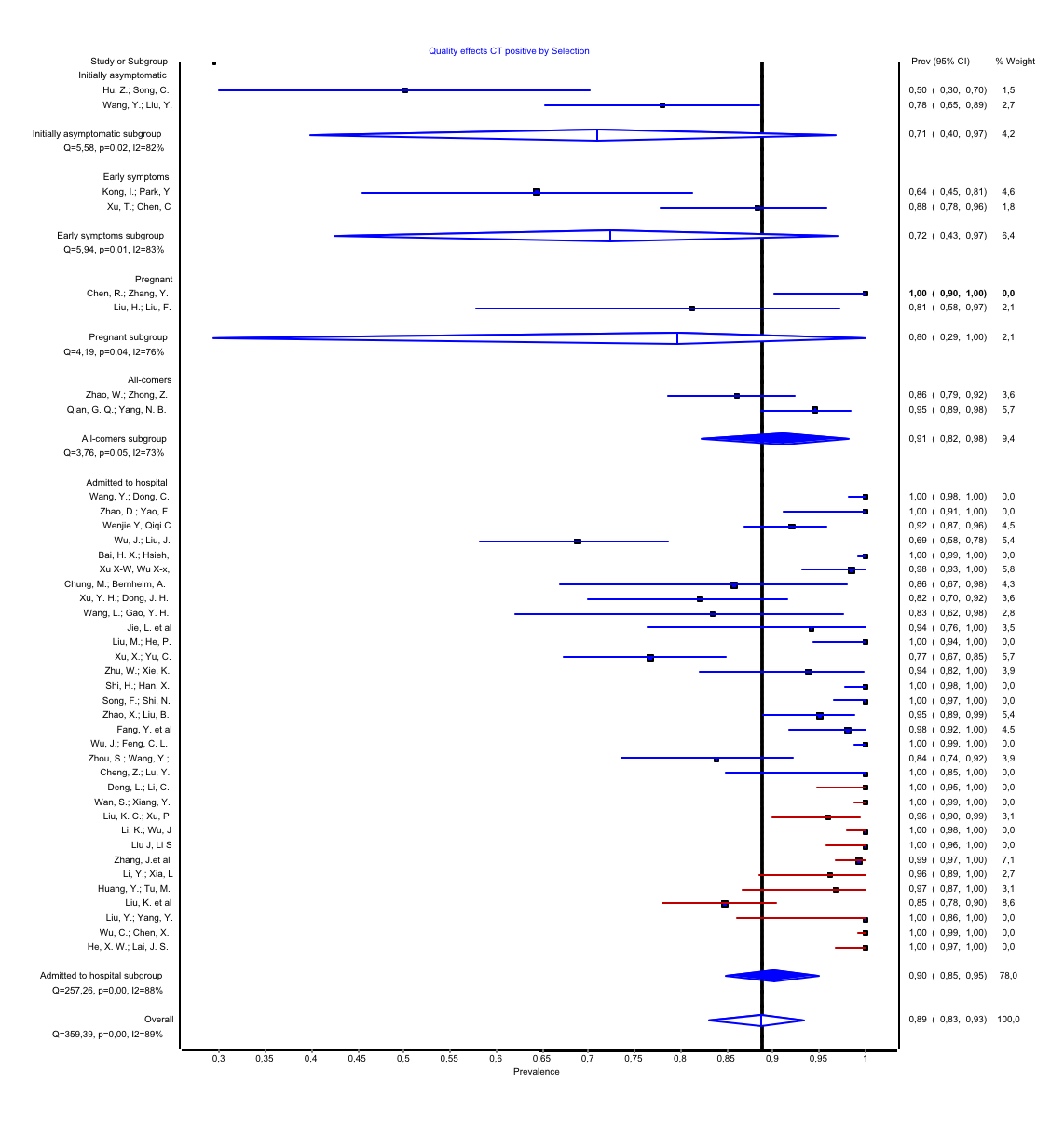


**S21 Figure. Meta-analysis of the incidence of positive CT findings.** Overall COVID 19 patient group


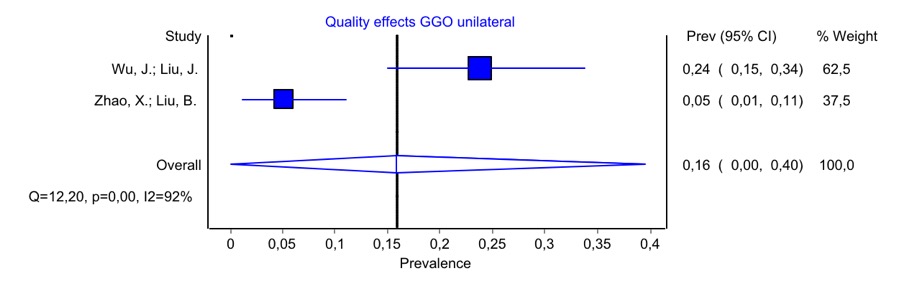


**S22 Figure. Meta-analysis of the incidence of GGO Unilateral patients**


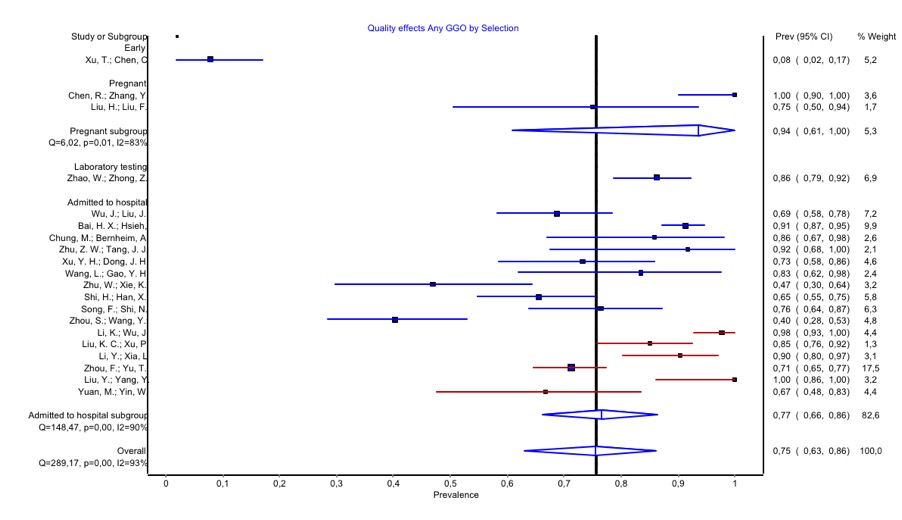


**S23 Figure. Meta-analysis of the incidence of any GGO**. Overall COVID 19 patient group


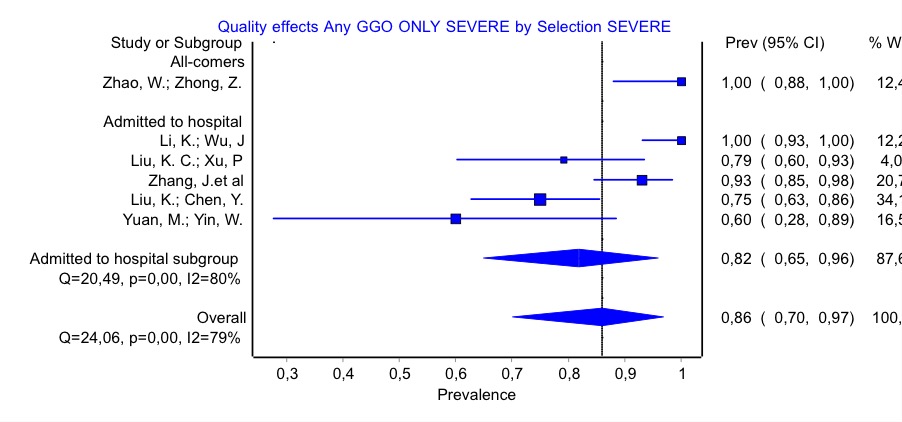


**S24 Figure. Meta-analysis of the incidence of any GGO**. Sseverely ill patient group


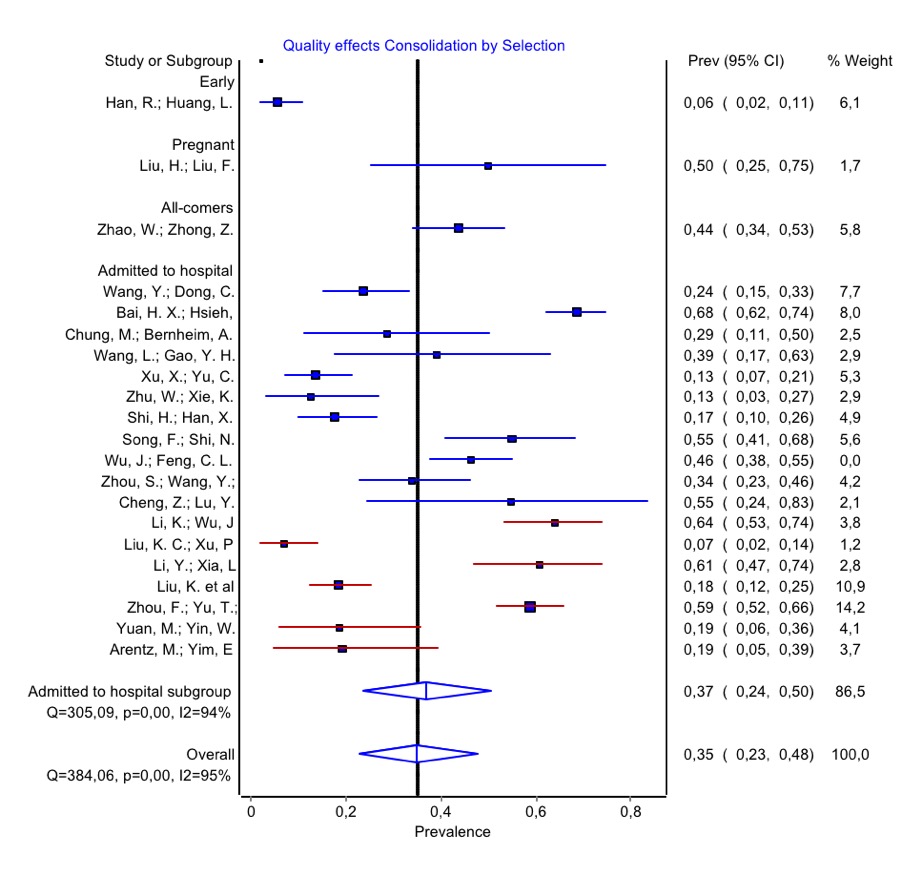


**S25 Figure. Meta-analysis of the incidence of consolidation.** Overall COVID 19 patient group


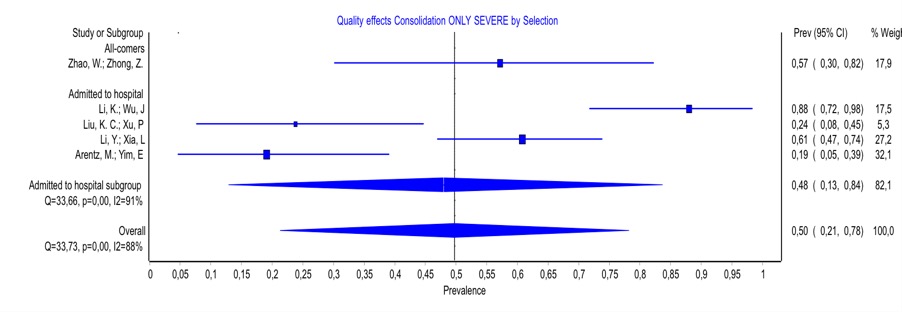


**S26 Figure. Meta-analysis of the incidence of consolidation**. Severely ill patient group


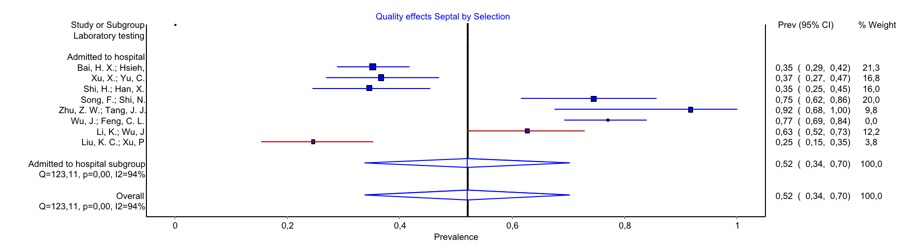


**S27 Figure. Meta-analysis of the incidence of septal thickening.** Overall COVID 19 patient group


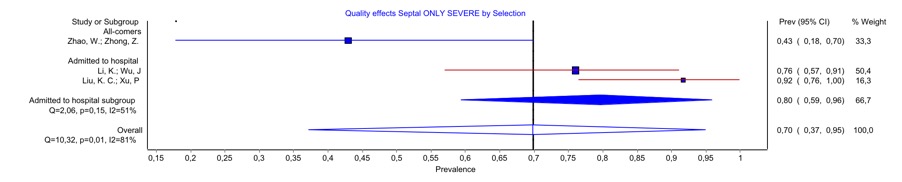


**S28 Figure. Meta-analysis of the incidence of septal thickening**. severely ill patient group


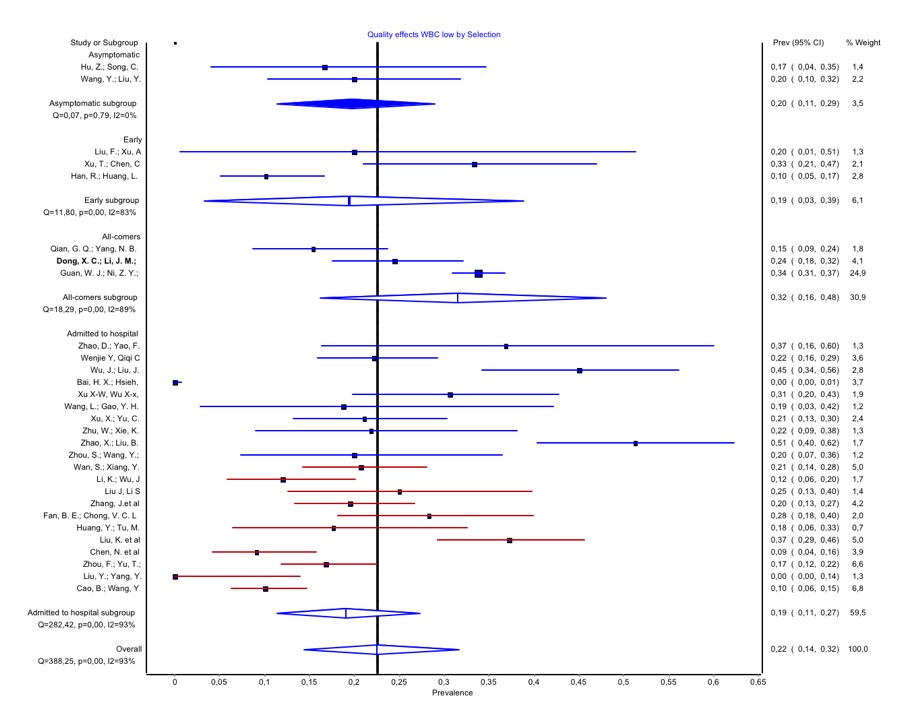


**S29 Figure. Meta-analysis of the incidence of low WBC**. Overall COVID 19 patient group


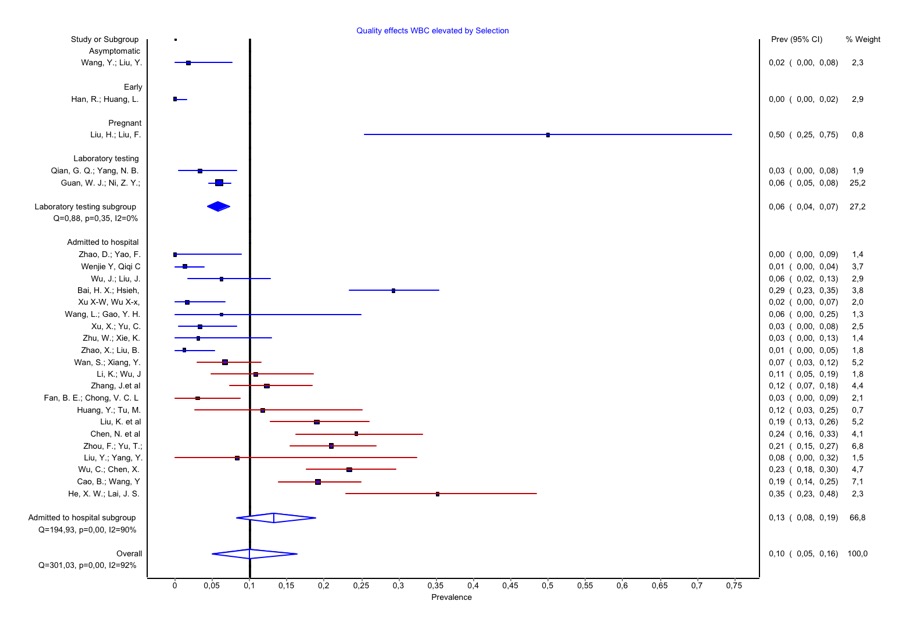


**S30 Figure. Meta-analysis of the incidence of elevated WBC**. Overall COVID 19 patient group


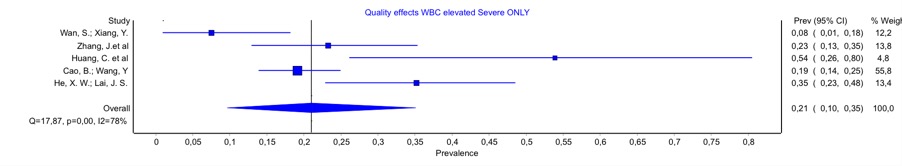


**S31 Figure. Meta-analysis of the incidence of elevated WBC**. Severely ill patient group


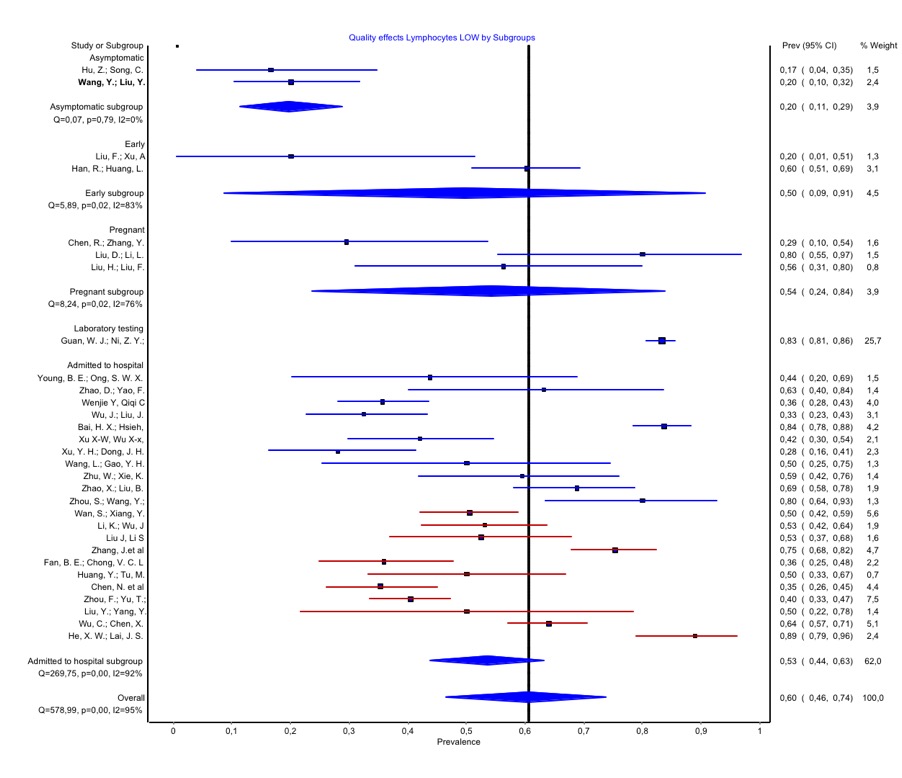


**S32 Figure. Meta-analysis of the incidence of low Lymphocytes**. Overall COVID 19 patient group


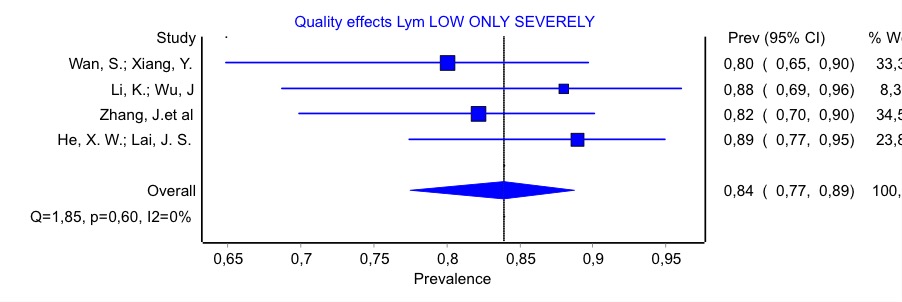


**S33 Figure. Meta-analysis of the incidence of low Lymphocytes**. Severely ill patient group


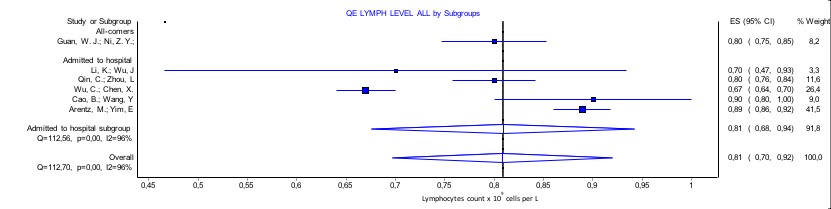


**S34 Figure. Meta-analysis of the incidence of overall Lymphocyteslevels**. Severely ill patient group


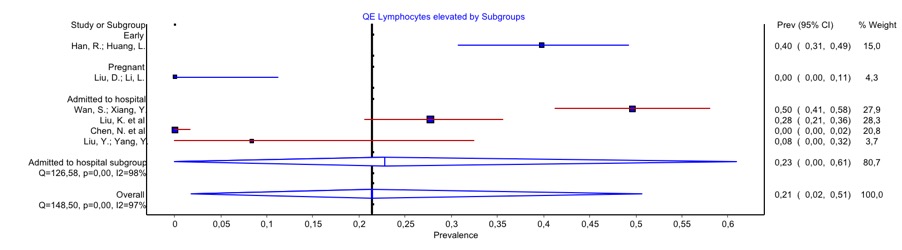


**S35 Figure. Meta-analysis of the incidence of elevated Lymphocytes**. Overall COVID 19 patient group


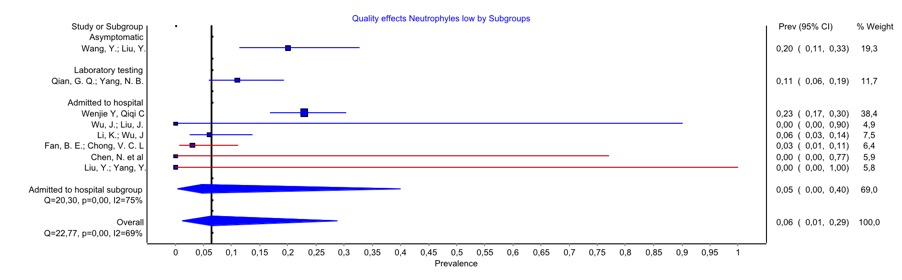


**S36 Figure. Meta-analysis of the incidence of low Neutrophils**. Overall COVID 19 patient group


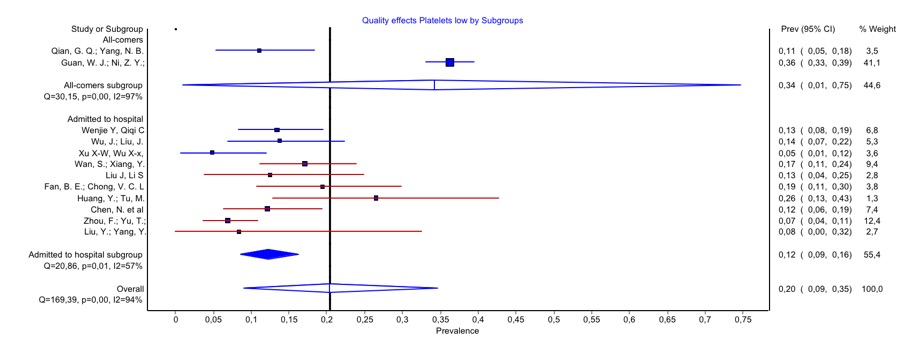


**S37 Figure. Meta-analysis of the incidence of low platelets.** Overall COVID 19 patient group


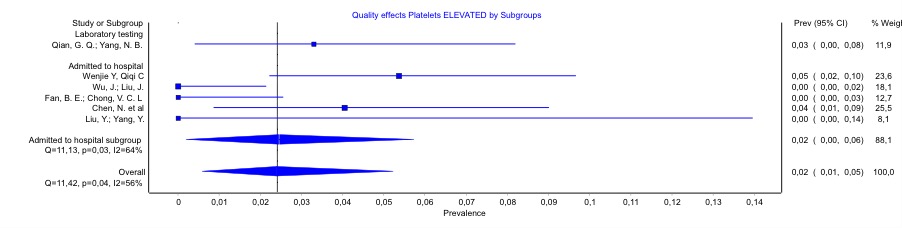


**S38 Figure. Meta-analysis of the incidence of elevated platelets**. Overall COVID 19 patient group


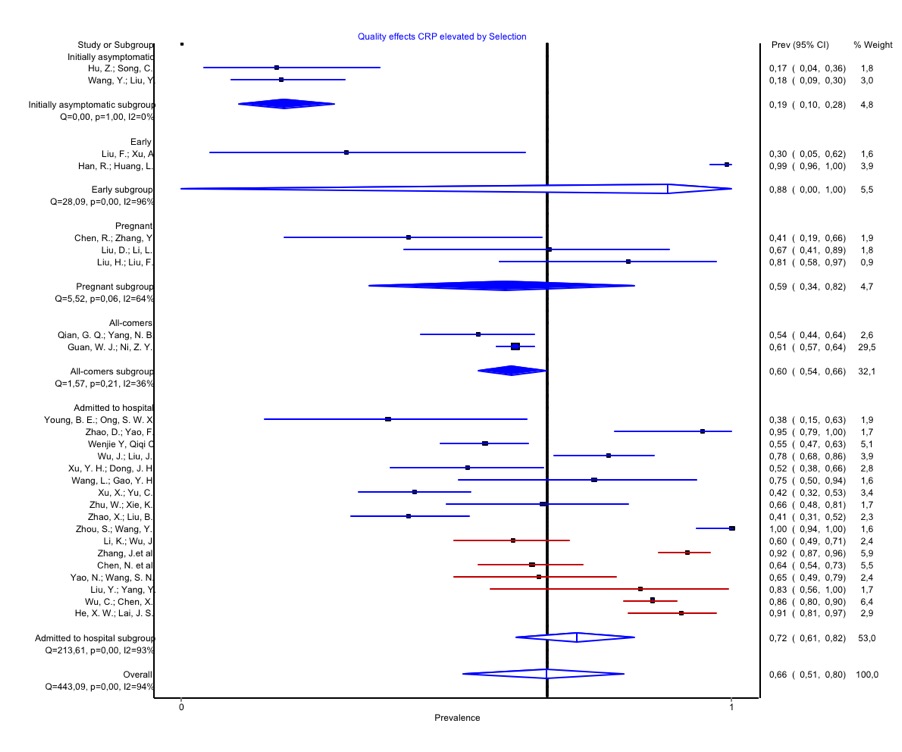


S39 Figure. **Meta-analysis of the incidence of elevated CRP.** Overall COVID 19 patient group


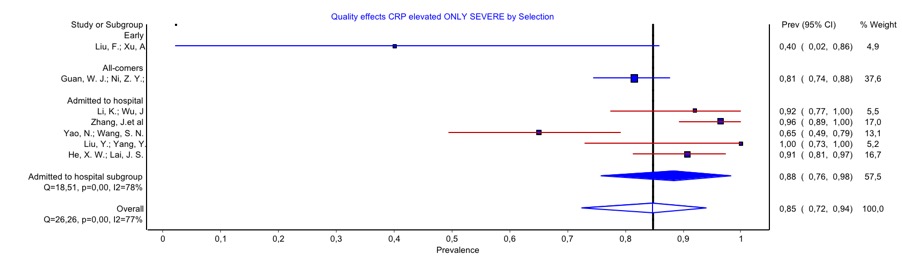


S40 Figure. **Meta-analysis of the incidence of elevated CRP**. Overall COVID 19 patient group


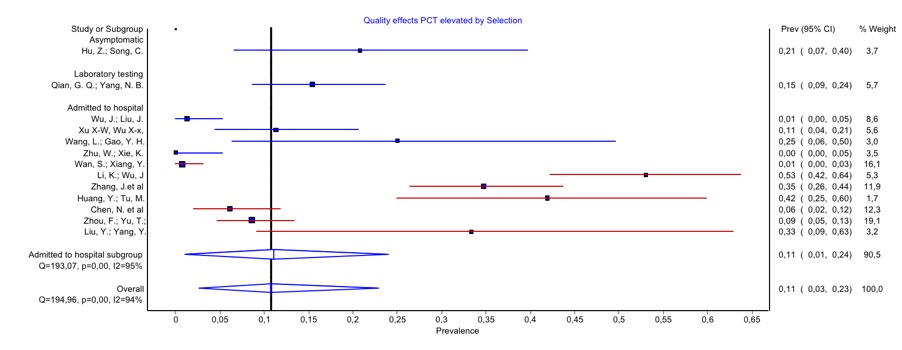


S41 Figure. **Meta-analysis of the incidence of elevated PCT.** Overall COVID 19 patient group


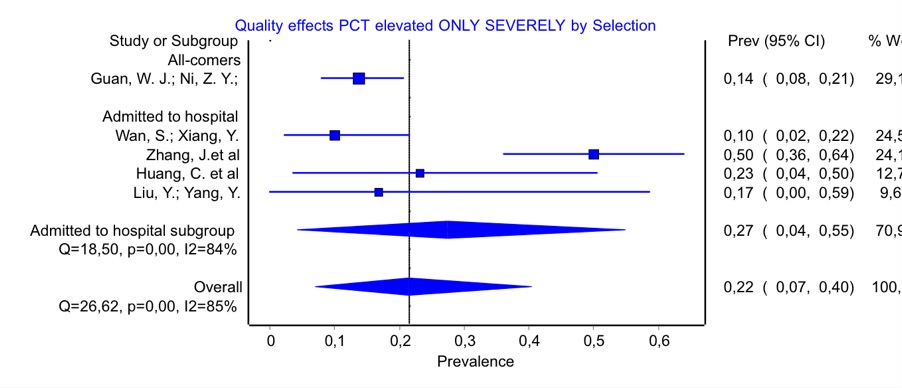


S42 Figure**. Meta-analysis of the incidence of elevated PCT**. Severely ill patient group


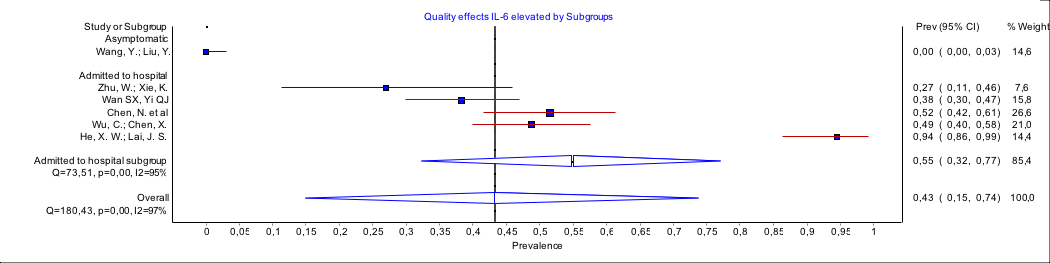


S43 Figure. **Meta-analysis of the incidence of elevated IL6**. Overall COVID 19 patient group


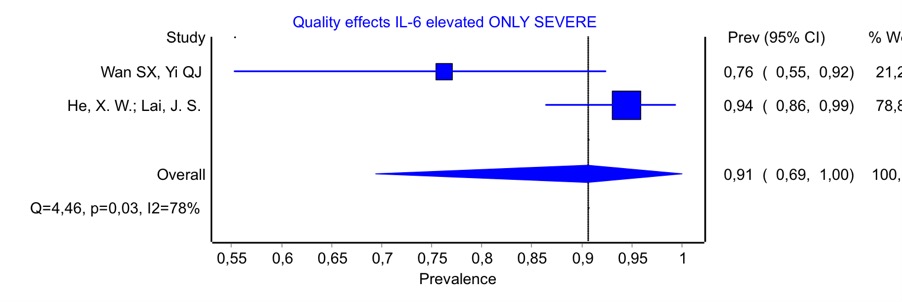


S44 Figure. **Meta-analysis of the incidence of elevated IL**. Severely ill patient group


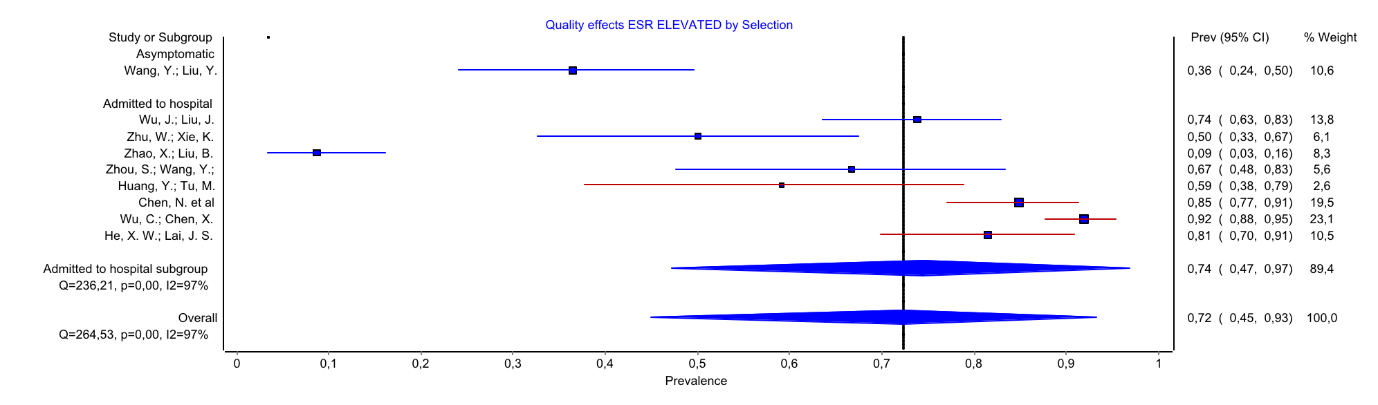


S45 Figure. **Meta-analysis of the incidence of elevated ESR**. Overall COVID 19 patient group


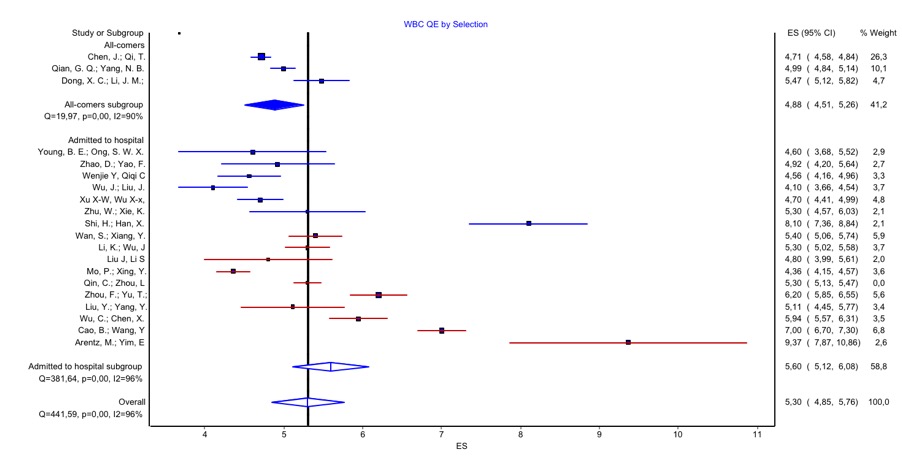


S46 Figure. **Meta-analysis of the incidence of CRP levels**. Overall COVID 19 patient group


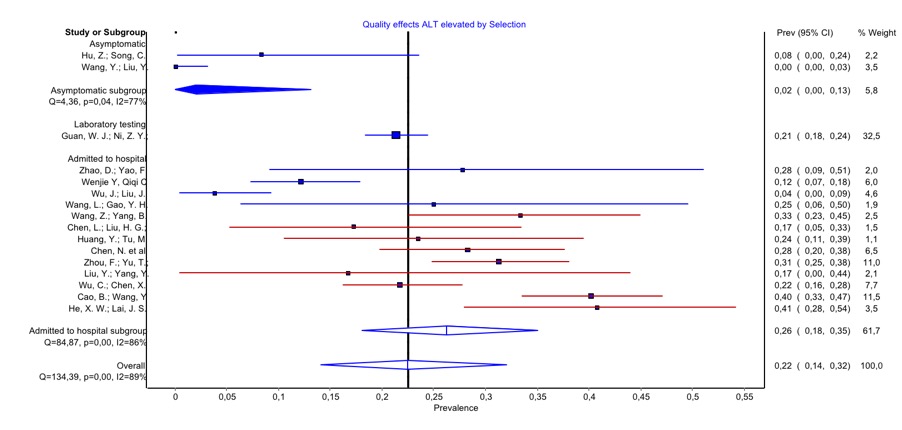


S47 Figure. **Meta-analysis of the incidence of elevated ALT**. Overall COVID 19 patient group


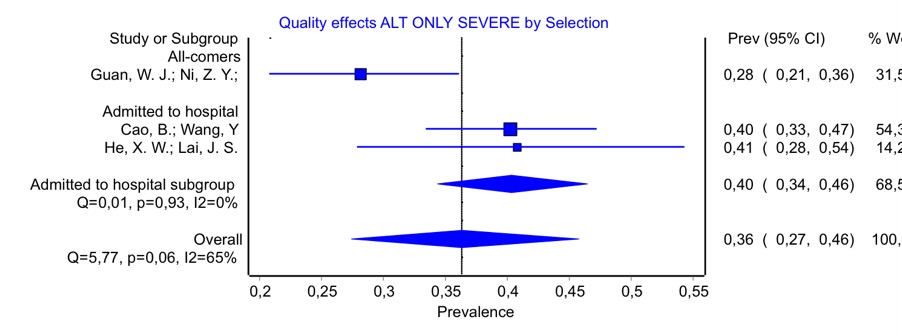


S48 Figure. **Meta-analysis of the incidence of elevated ALT**. Severely ill patient group


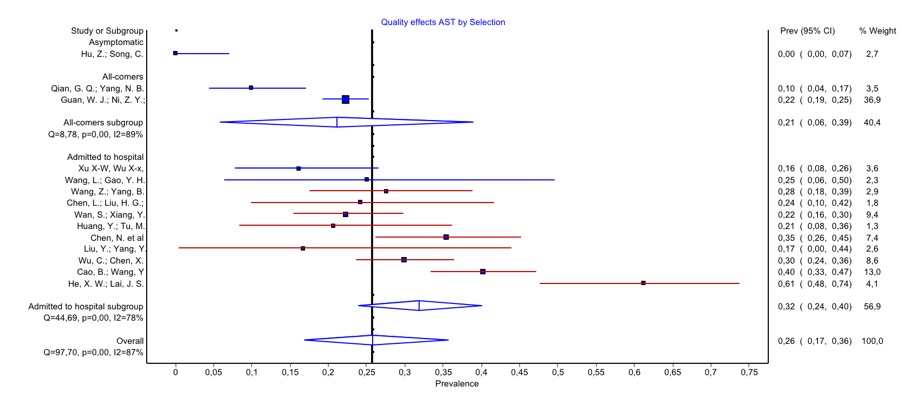


S49 Figure. **Meta-analysis of the incidence of elevated AST.** Overall COVID 19 patient group


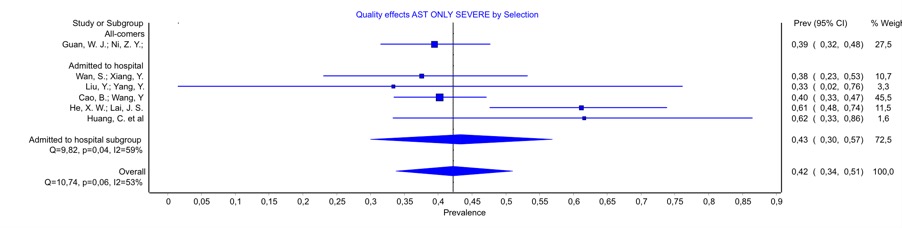


S50 Figure. **Meta-analysis of the incidence of elevated AST**. Severely ill patient group


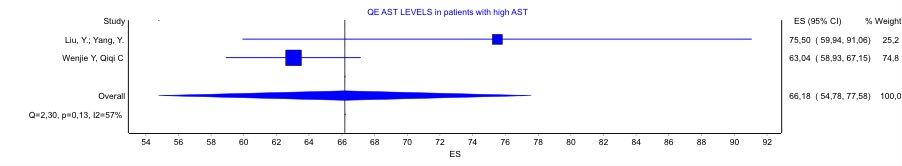


S51 Figure. **Meta-analysis of the incidence of elevated AST levelSin those with high AST**. Overall COVID 19 patient group


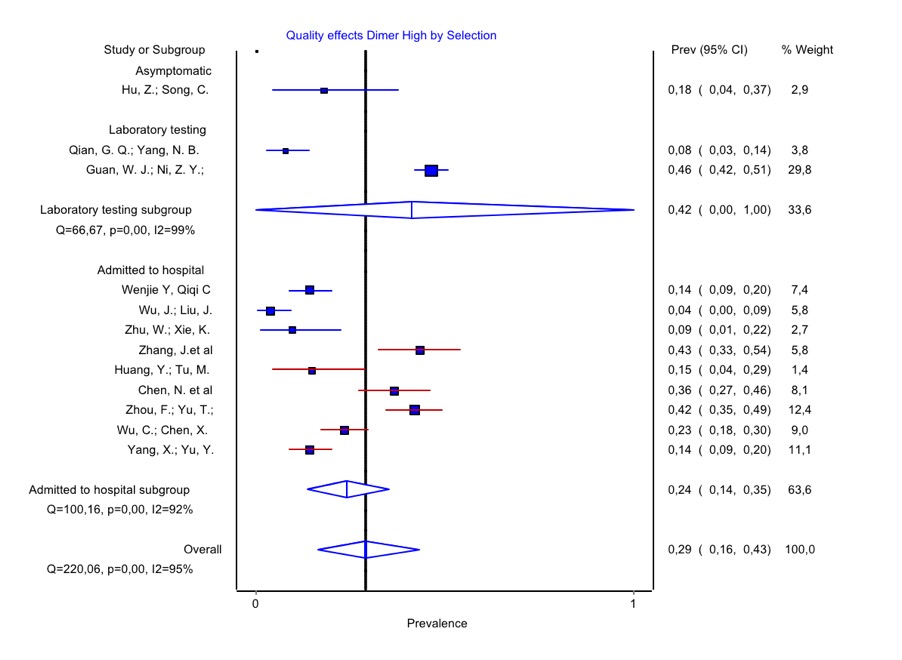


S52 Figure. **Meta-analysis of the incidence of high dimer**. Overall COVID 19 patient group


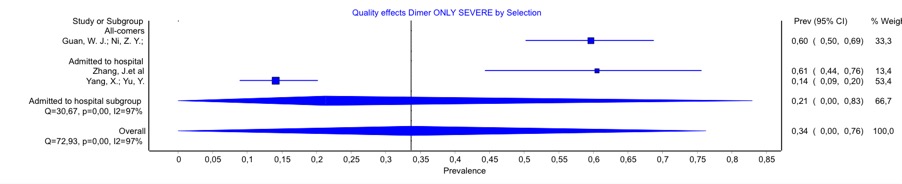


S53 Figure. **Meta-analysis of the incidence of high dimer**. Severely ill patient group


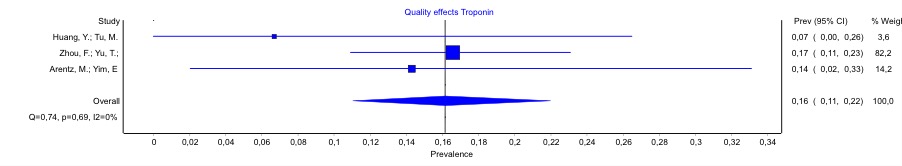


S54 Figure. **Meta-analysis of the incidence of Troponin**. Overall COVID 19 patient group


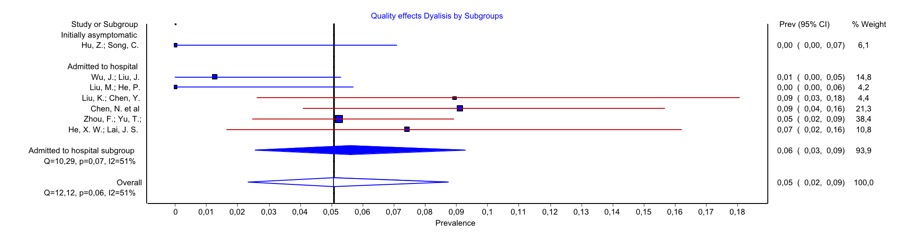


S55 Figure. **Meta-analysis of the incidence of Dialysis.** Overall COVID 19 patient group


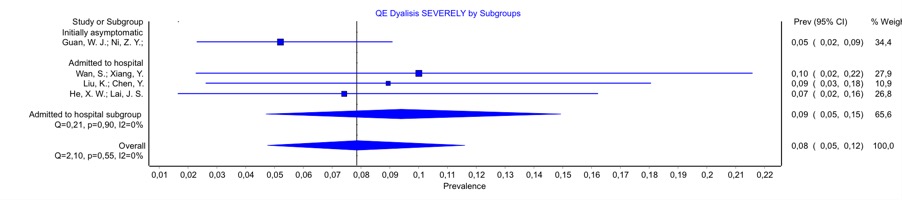


S56 Figure. **Meta-analysis of the incidence of severely ill**. Overall COVID 19 patient group


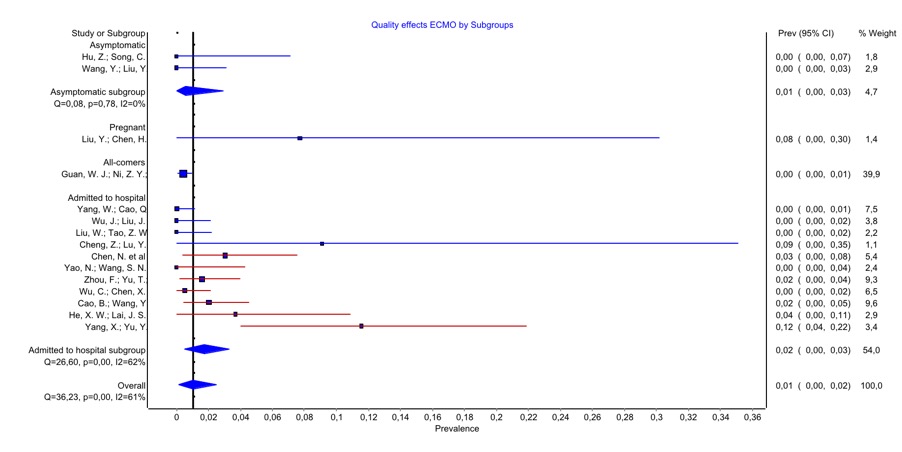


S57 Figure. **Meta-analysis of the incidence of ECMO**. Overall COVID 19 patient group


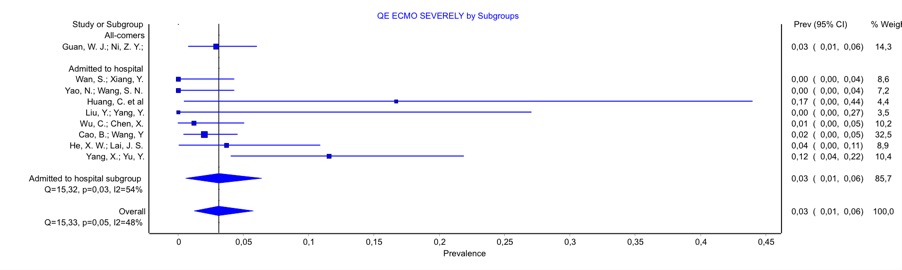


S58 Figure. **Meta-analysis of the incidence of ECMO**. Severely ill patient group


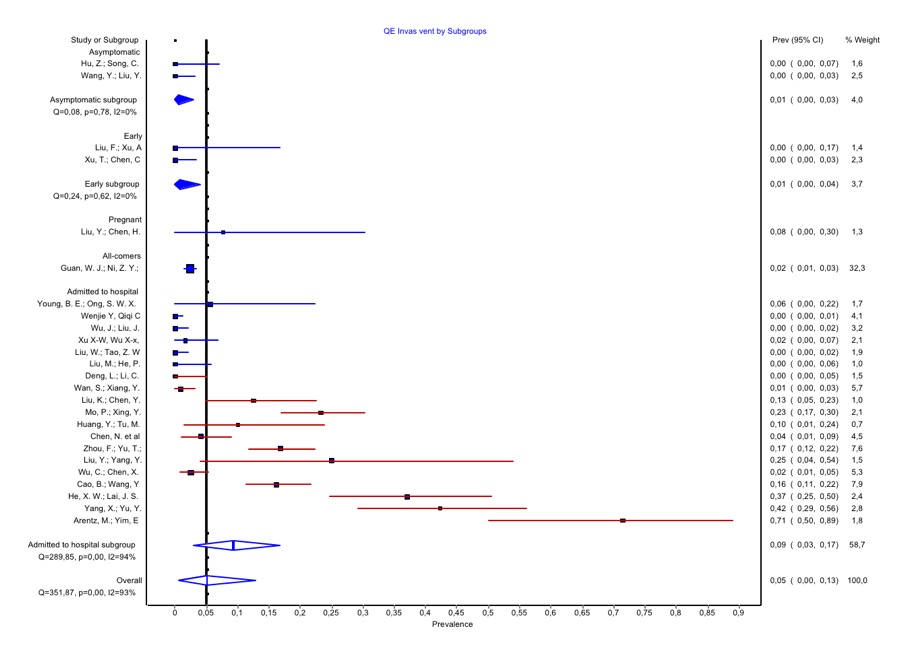


S59 Figure. **Meta-analysis of the incidence of invasive ventilation**. Overall COVID 19 patient group


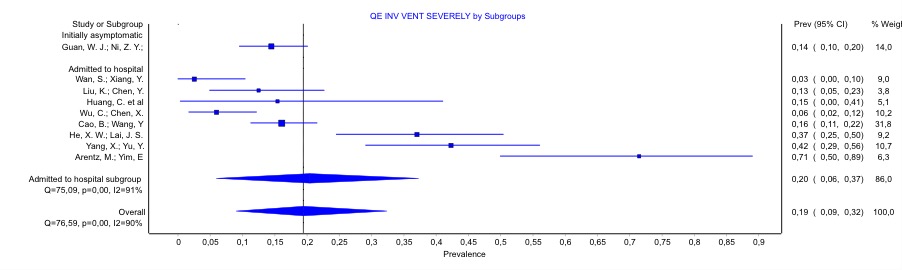


S60 Figure. **Meta-analysis of the incidence of invasive ventilation**. Severely ill patient group


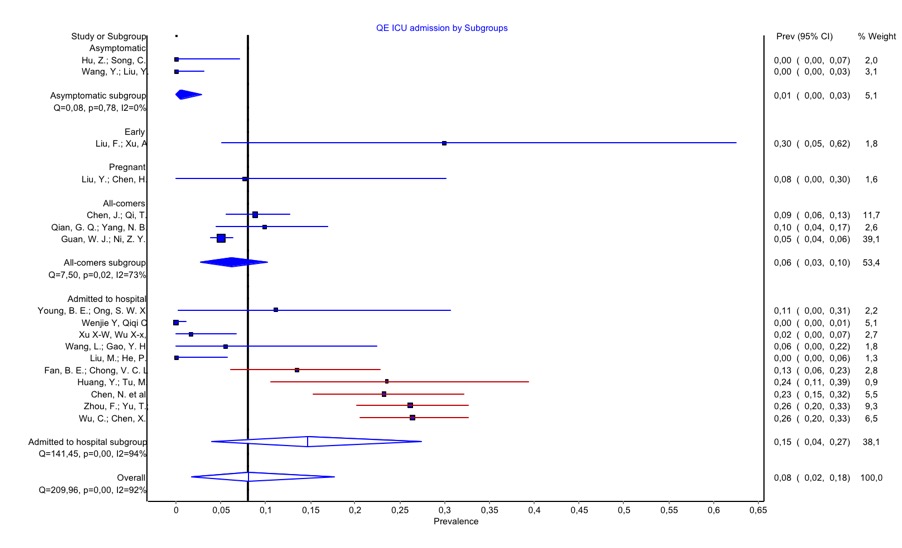


S61 Figure. **Meta-analysis of the incidence of ICU admission**. Overall COVID 19 patient group.


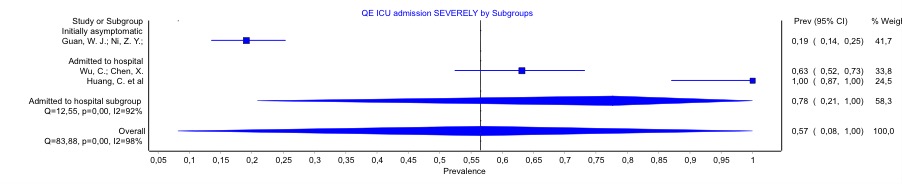


S62 Figure**. Meta-analysis of the incidence of ICU admission**. Severely ill patient group.


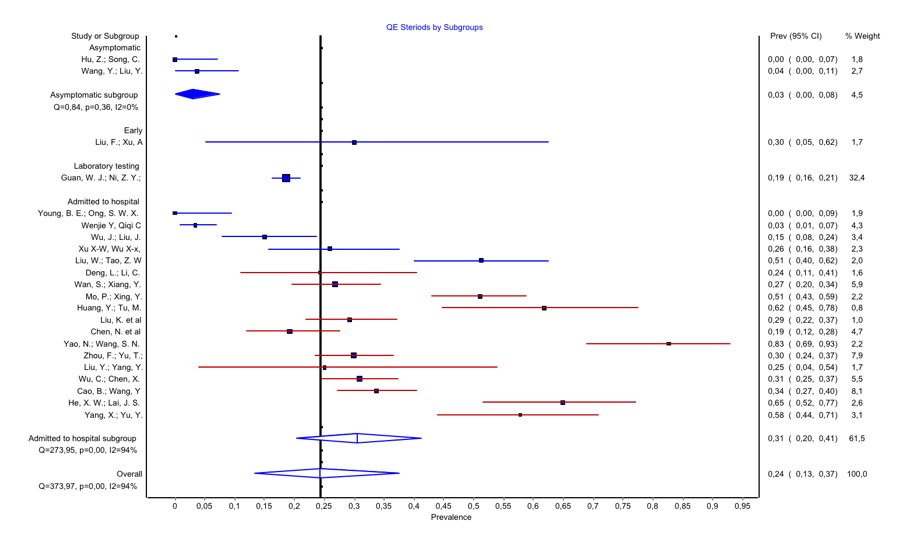


S63 Figure. **Meta-analysis of the incidence of Steroids**. Overall COVID 19 patient group.


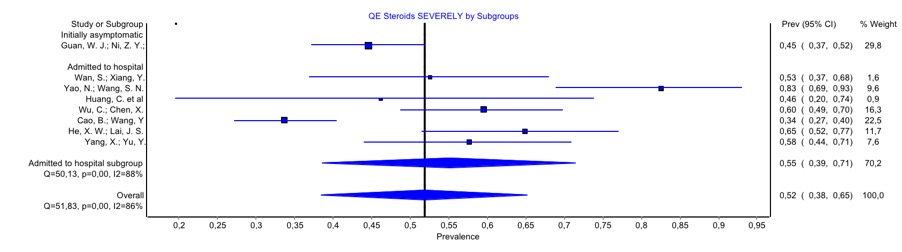


S64 Figure. **Meta-analysis of the incidence of Steroids**. Severely ill patient group.


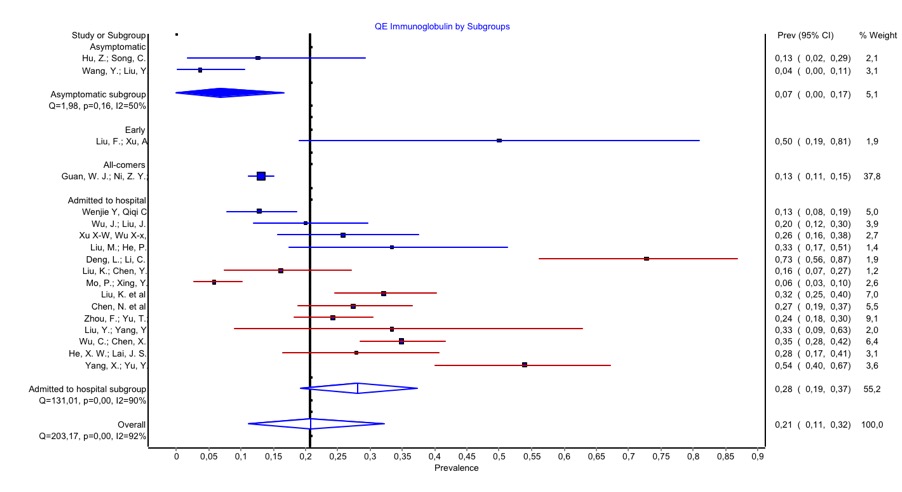


S65 Figure. **Meta-analysis of the incidence of Immunoglobulin**. Overall COVID 19 patient group.


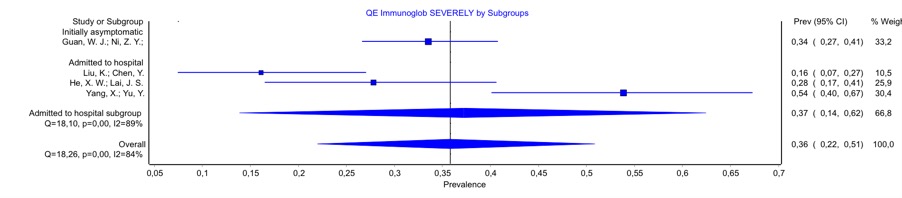


S66 Figure. **Meta-analysis of the incidence of Immunoglobulin**. Severely ill patient group.


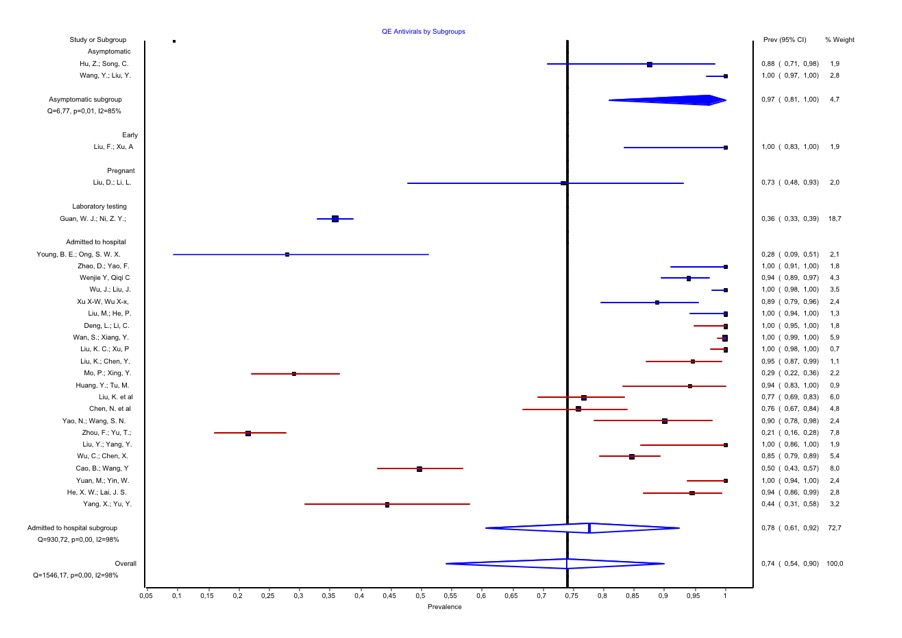


S67 Figure. **Meta-analysis of the incidence of Antivirals**. Overall COVID 19 patient group.


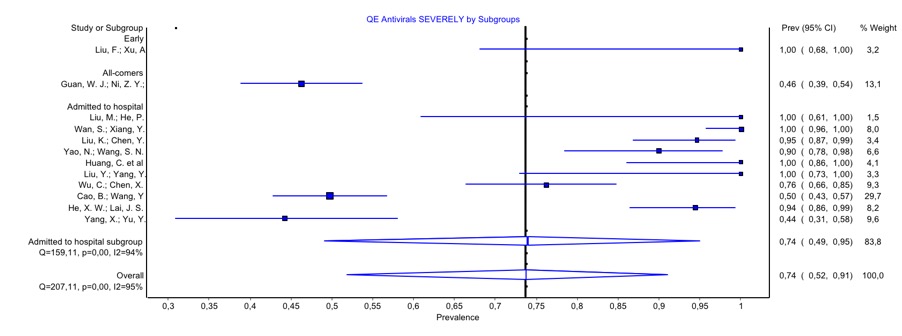


S68 Figure. **Meta-analysis of the incidence of Antiviral**. Severely ill patient group.


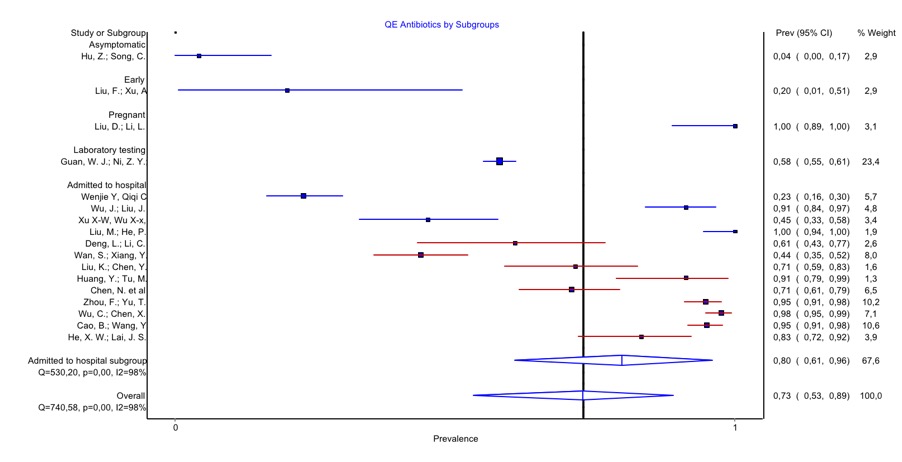


S69 Figure. **Meta-analysis of the incidence of Antibiotic**. Overall COVID 19 patient group.


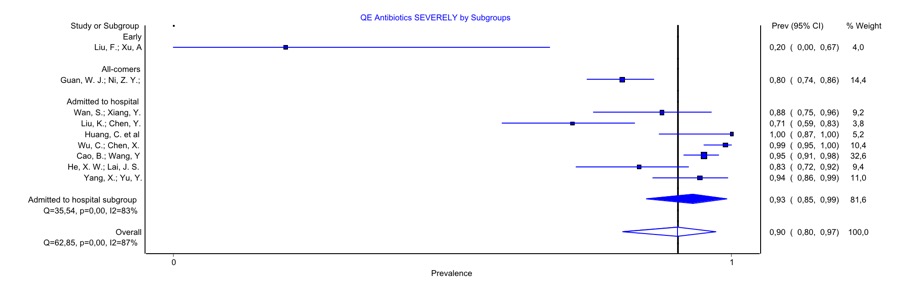


S70 Figure. **Meta-analysis of the incidence of Antibiotic**. Severely ill patient group.


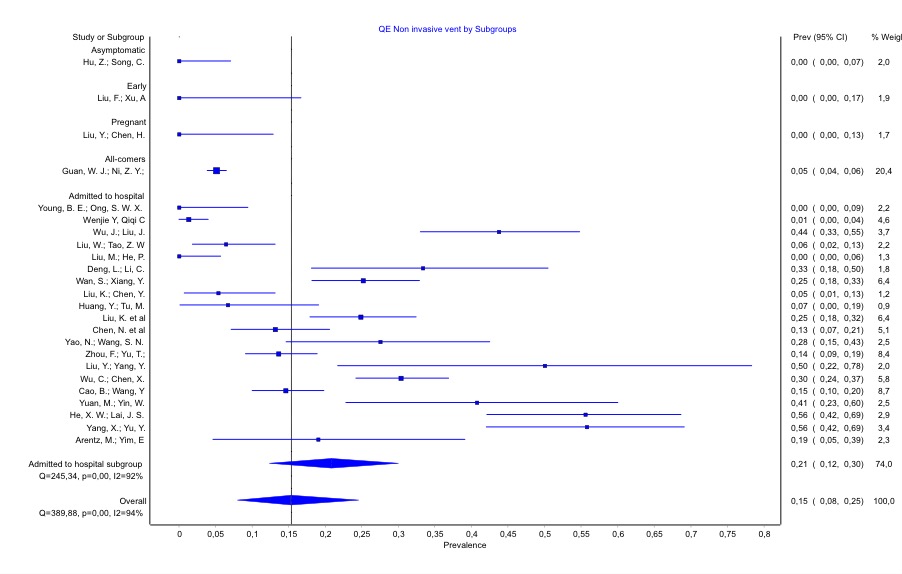


S71 Figure. **Meta-analysis of the incidence of non-invasive ventilation**. Overall COVID 19 patient group.


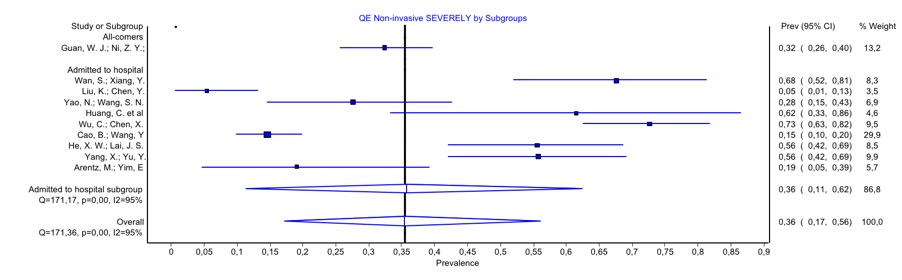


S72 Figure. **Meta-analysis of the incidence of non-invasive ventilation**. Severely ill patient group.


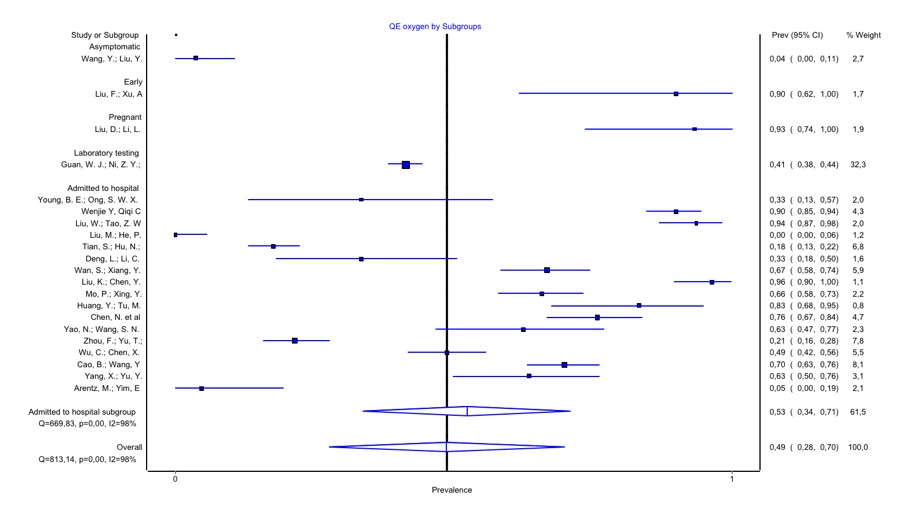


S73 Figure**. Meta-analysis of the incidence of Oxygen therapy**. Overall COVID 19 patient group.


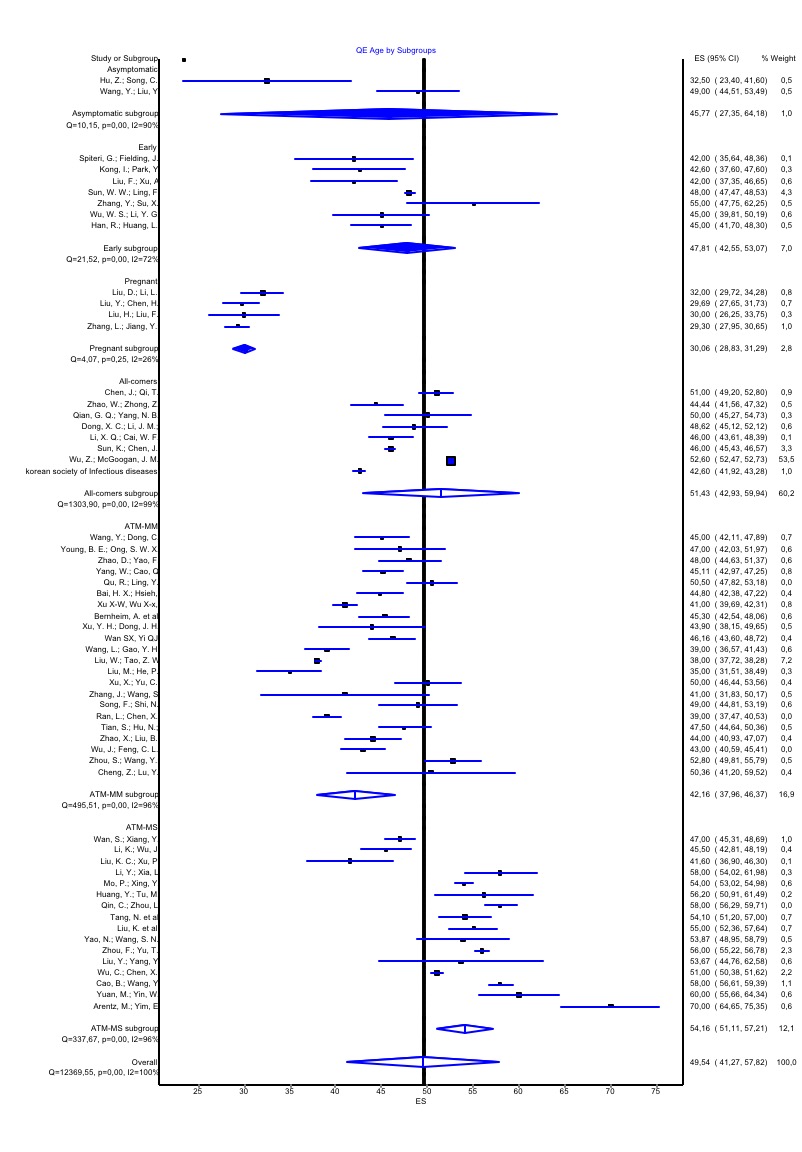


S74 Figure. **Meta-analysis of the incidence of age**. Overall COVID 19 patient group.


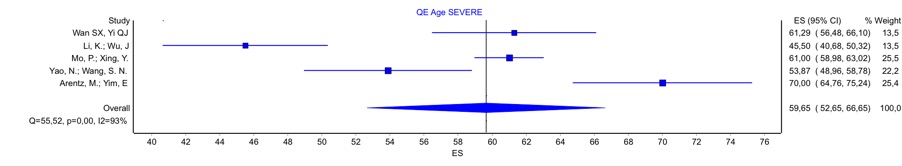


S75 Figure. **Meta-analysis of the incidence of age**. Severely ill patient group.


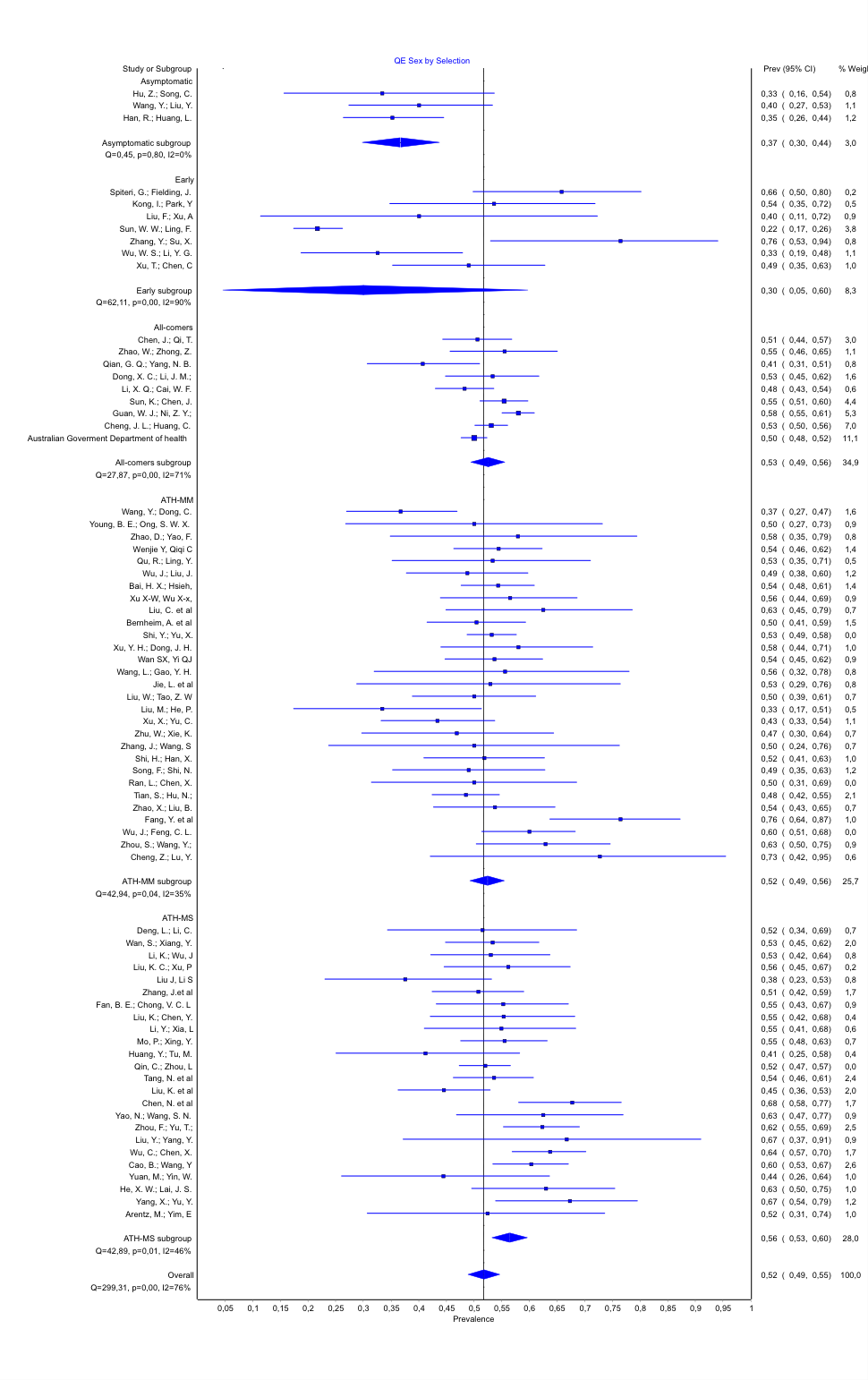


S76 Figure. **Meta-analysis of the incidence of Sex**. Overall COVID 19 patient group.


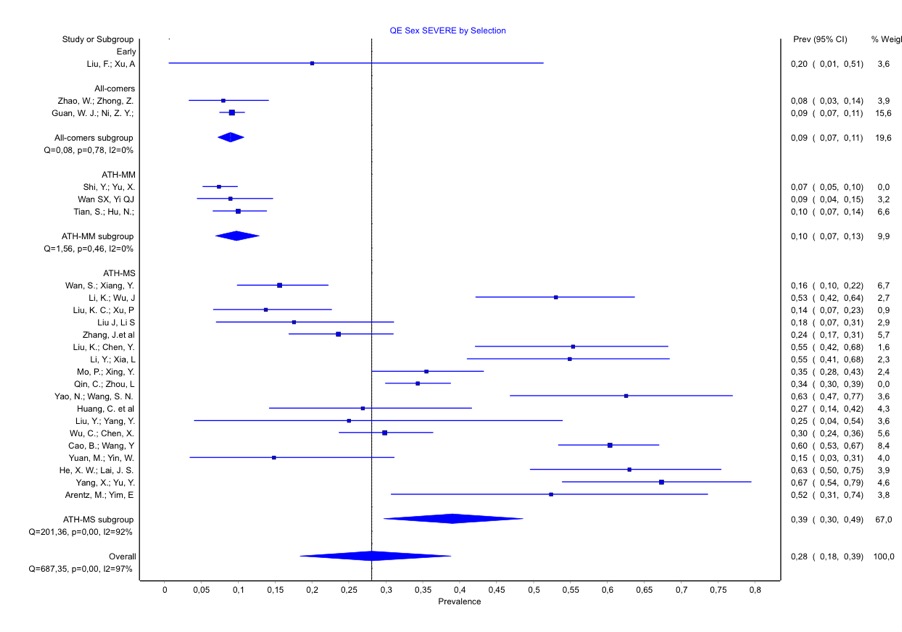


S77 Figure. **Meta-analysis of the incidence of Sex**. Severely ill patient group.


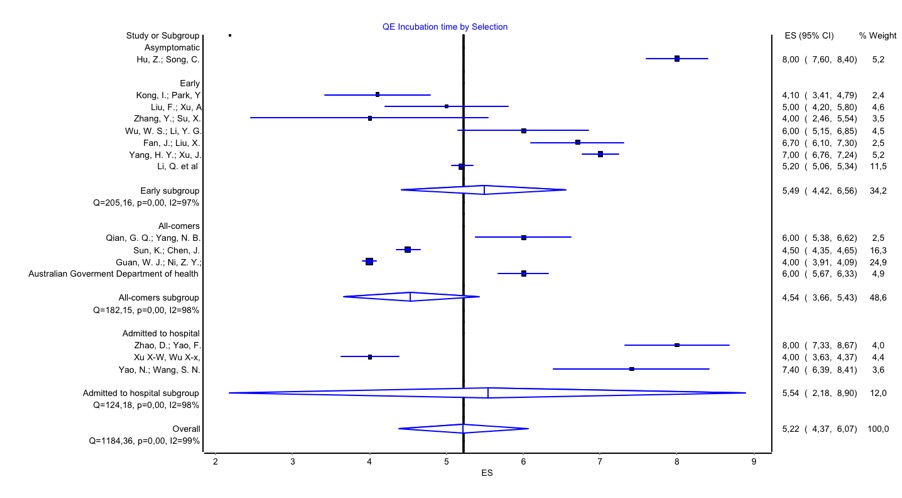


S78 Figure. **Meta-analysis of the incidence of incubation time**. Overall COVID 19 patient group.


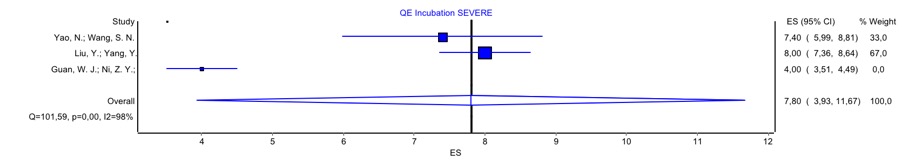


S79 Figure. **Meta-analysis of the incidence of incubation time**. Severely ill patient group.


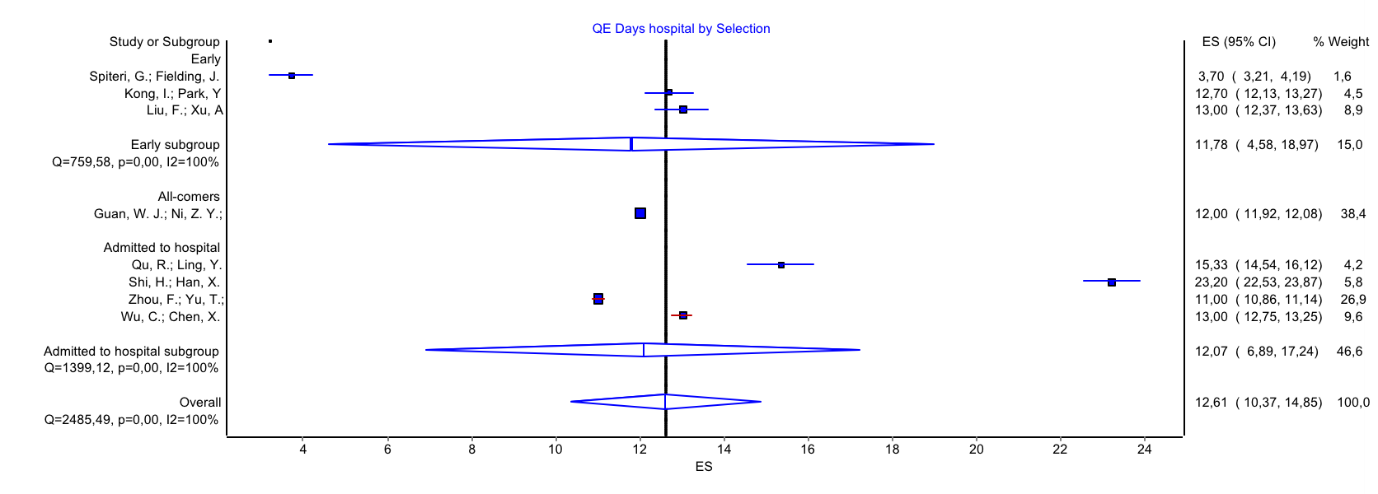


S80 Figure**. Meta-analysis of the incidence of length of hospital stay**. Overall COVID 19 patient group.
