# Supplementary material for: Clinical, laboratory and radiological characteristics and outcomes of novel coronavirus (SARS-CoV-2) infection in humans: A systematic review and series of meta-analyses
Source: PLoS One. 2020 Sep 17;15(9):e0239235. doi: 10.1371/journal.pone.0239235 (PMC7498028; doi:10.1371/journal.pone.0239235)
Supplement: Attachment — Submitted filename: Rebuttal letter_ PLoS One.docx [file pone.0239235.s002.docx]

August 8, 2020

Belo Horizonte, Brazil

Dear Dr Farag,

We thank you and reviewers for the generous and eloquent comments on our manuscript: ‘Clinical, laboratory and radiological characteristics and outcomes of novel coronavirus (SARS-CoV-2) infection in humans: A systematic review and series of meta-analyses’, submitted for publication to PLOS ONE. The manuscript has been revised according to your comments and herewith we are submitting the revised manuscript for your kind review, including the point-by-point responses. Please note that we opted not to proceed with updating the paper beyond our original search dates because this would require repetition of almost the entire study. Please see the responses to Reviewer #2’s comments 2 and 4 for a detailed explanation. We believe that with the suggested revisions we are presenting here solid scientific work, done with rigorous methodology, which shows important associations of various clinical parameters with the severity of COVID-19. We would be happy to provide further details if necessary. We would also be happy to allow communications of the peer-review process for open (published) peer-review.

With kind regards,

Israel Júnior Borges do Nascimento, Thilo Caspar von Groote, Ana Jeroncic

for all authors.

Response to reviewer

1. **Please ensure that your manuscript meets PLOS ONE's style requirements, including those for file naming. The PLOS ONE style templates can be found at https://journals.plos.org/plosone/s/file?id=wjVg/PLOSOne_formatting_sample_main_body.pdf and https://journals.plos.org/plosone/s/file?id=ba62/PLOSOne_formatting_sample_title_authors_affiliations.pdf**

Thank you. We have edited the manuscript according to the formal requirements for publication in PLOS One.

2. **We note that one or more of the authors are employed by a commercial company: Swanscoe Communications Ltd. 2.1. Please provide an amended Funding Statement declaring this commercial affiliation, as well as a statement regarding the Role of Funders in your study.**

Thank you for the comment. Swanscoe Communications Ltd. had no role in designing, conducting or analyzing data in our study. CH supported the writing of the manuscript *pro bono* and this research work is unrelated to her work at Swanscoe Communications.

We have now amended the funding statement:

“We have read the journal's policy and the authors of this manuscript have the following potential competing interests: CH is employed by Swanscoe Communications Ltd, a medical writing company, and has previously provided medical writing support formedical and pharmaceutical projects, unrelated to the current work. Medical writing for the current work was provided by CH pro bono and was not funded by any pharmaceutical or device company. This does not alter our adherence to PLOS ONE policies on conflicts of interest. All other authors declare that no competing interests exist.”

3. **One of the noted authors is a group or consortium [International Task Force Network of Coronavirus Disease 2019 (InterNetCOVID-19)]. In addition to naming the author group, please list the individual authors and affiliations within this group in the acknowledgments section of your manuscript. Please also indicate clearly a lead author for this group along with a contact email address.**

We thank the reviewer for highlighting this. We have now re-structured the list of participants along with their respective affiliations.

4. **Please include captions for your Supporting Information files at the end of your manuscript, and update any in-text citations to match accordingly. Please see our Supporting Information guidelines for more information:** [**http://journals.plos.org/plosone/s/supporting-information**](http://journals.plos.org/plosone/s/supporting-information)**.**

Thank you for the comment. We now have included the captions and also updated the citations as required.

**Reviewer #1**:

5. **Thank you for the opportunity to review this work. I have several comments on this meta-analysis. In general, I agree with the idea of the analysis in that there is a need to identify and establish clarity on a lot of key outcome issues with the COVID 19 pandemic. In general, this is good work, and a very comprehensive analysis that has been done using appropriate methodology for a meta analysis. The linkage of disease severity with age, sex, radiology and labs is important and is still being used to guide prognostication and therapy.**

**Some issues that I would like the authors to answer:**

**1. Date range chosen 01-01-2019 and 03-22-2020. Considering 01-01-2019 ——> was there any coronavirus cases anywhere in the world at that time? To my best knowledge, the earliest diagnosed cases where November, 2019 in Wuhan, China. What is the objective of going back all the way to Jan,2019? Can the authors actually show us if they identified any published work from before November, 2019-Jan 2019?**

Thank you for noticing this. Indeed, the date ranged from 01-01-2019 to 03-22-2020. We decided to be more conservative regarding time of search because we did not want to miss any possible eligible studies. Therefore, our library specialist advised us to use a broader date range in order to obtain any coronavirus-related article, even before the actual starting date of the novel coronavirus infection period. Even though we have defined the search before november 2019, we did not find any eligible study before January, 2020.

**2. I also remain concerned about the very inclusive “all types of research’ accepted in inclusion criteria. One problem with COVID19 research and or data is the lack of value from sample size datasets. We have seen along the evolution of science associated with the disease that small datasets have fueled speculation and added nothing to actual evidence and sometimes when prospective observational n=10 studies have shown ‘encouraging’ outcomes, these have not been validated by larger studies especially the larger studies with adequate controls. Did the authors consider breaking down their results by types of studies? I dare say that there were not many RCTs in the picture in March 2020 when this work was done. Therefore a lot of the included studies here are case series. While I agree that the initial impetus from Wuhan was to publish a lot of case series, now things are very different**.

We thank the reviewer for this comment. We agree that in a single small study probabilities of type II, as well as type I error are increased. The effect of studies with small sample size is, however, mitigated in meta-analyses. Namely, by combining multiple studies and taking into account precision of their estimates, the overall power and precision of the pooled estimate in meta-analyses increase. For that reason, the Cochrane Collaboration doesn't consider sample size of individual studies as a source of bias. In addition, by excluding studies with a sample size of at least 10 patients (compared to our previously published review), we have included only 23 (28%) of studies with a relatively small sample size of N≤30 in our analyses (8 or 10% with N≤15). Given that not all studies reported all analyzed characteristics, only a very small subset of small studies was included in an individual meta-analysis. Having said that we consider that the effect of small studies on our results is mitigated. To give an illustration on the number of patients and number of studies included in each meta-analysis we have updated Tables 2-6 with these data.

We have considered the sample size of individual studies an issue only when it affected estimated effect size – more specifically in meta-analysis for the outcome of ‘mortality’. Namely, the minimal fatality rate that could be detected in studies with small sample size ranged between 3% (1 dead out of 30) and 10% (one dead out of 10). Therefore, if a real effect was lower than this minimum, it is likely that no death would have been observed in a study and 0% of fatality rate would be reported, therefore deflating real fatality rate. This issue was highlighted in the manuscript under the section on mortality Pg 13 „*However, study sample sizes and follow-up time were usually inadequate to be able to observe those events.*“.

As for the types of research included, given the limited research work on COVID-19 conducted during the time of the search, we included all the types of study designs in order to provide a comprehensive overview of the scientific evidence. We included 45 consecutive retrospective case series, 2 consecutive prospective case series, and 29 case series that were also likely consecutive (included all patients admitted within a period) but did not report sampling in sufficient detail. Additionally, we included one RCT, and 9 epidemiological reports which only provided data on age, gender, incubation time, and comorbidities. Therefore, in terms of type of research our data were quite homogeneous with the studies predominantly being consecutive case series. As our primary aim was to investigate association of various clinical characteristics with severity of COVID-19 status in the COVID-19+ patient population, the RCT design would not be optimal given its purpose in investigating causality in experimental studies. Instead, (prospective) observational studies with consecutive sampling are more appropriate. Due to the nature of the COVID-19 disease, a true cohort study in which COVID-19+ patients are aligned in time (i.e. assessed at same time after the infection) and observed for the same period of time is not feasible. Consequently, consecutive case studies remain the design that prevails as the major source of information for COVID-19 research questions on prediction/diagnosis.

**3. I would strongly encourage the authors to make their work more concise and crisp. I know that this is a series of several meta-analysis put together, but it is exhausting for an average reader to get through this paper and take any clinical benefit from it, since the messages are lost in a lot of textual information.**

Thank you for your comment which we agree with. We reviewed the paper completely with an aim to make it more concise and crisp, thus removing hundreds of words. Most of the results on heterogeneity and meta-regressions are moved to Tables 2-6 and only the findings are discussed in text. We hope that the reviewer will agree that this has made the manuscript as concise as possible while reviewing such a large body of scientific evidence.

**4. Finally, the evidence of science and related outcomes is constantly progressing in COVID19. Would the authors want to add relevant information for the reader to bring this work up to the mark with what is happening now? Would the addition of new studies that have been published from March till date make a big difference in their outcomes?**

Thank you for your suggestion. As the reviewer pointed out the number of published COVID-19 papers has been increasing at a very fast rate. While additional studies would definitively improve inference for the meta-analyses in our manuscript that lack an adequate number of studies/patients, in most meta-analyses the number of patients and number of studies was not an issue. Some of the data were redundant even by the end of March 2020 (e.g. data on the effect of age, gender, or symptoms like fever). Instead, it was the considerable heterogeneity of studies that hindered more clear answers for some questions. The likely cause of this heterogeneity was variability in timing of patient's enrollment from the infection and potentially heterogeneity in clinical, laboratory and radiological manifestations of COVID-19. As the incubation period of COVID-19 is quite wide, symptoms may occur for different patients at different times and people are assessed for the first time at different stages of a disease. This heterogeneity stemming from the nature of the disease is not expected to be mitigated in new studies either. Having said that, this manuscript may also be viewed as a reference for characteristics of COVID-19 described in the early studies.

It should also be noted that the work presented in this manuscript is comprehensive, not just in terms of data extraction but also in terms of synthesis of the evidence. More than 50 variables, including patients' characteristics and descriptive data on hospital admissions or treatments received, were meta-analysed in the study. By taking into account additional sub-analyses that were performed to replicate the finding on a particular variable in a population of severely ill patients or to investigate the sources of heterogeneity with meta-regression, around 130 analyses were performed. The inclusion of the new data would imply that all of these meta-analyses should be rerun. In addition, due to an increase in the number of publications, the search strategy would likely need to be rebuilt to include more specific filters, and data extraction and all analyses for the new studies would need to be performed again. This would amount to a completely new study. We therefore request that the journal reconsider this manuscript with its revisions as suggested by the helpful comments from the reviewers.
